# A fresh look at the role of spiramycin in preventing a neglected disease: meta-analyses of observational studies

**DOI:** 10.1186/s40001-021-00606-7

**Published:** 2021-12-11

**Authors:** Jose G. Montoya, Katherine Laessig, Mir Sohail Fazeli, Gaye Siliman, Sophie S. Yoon, Elizabeth Drake-Shanahan, Chengyue Zhu, Akbar Akbary, Rima McLeod

**Affiliations:** 1 Jack S. Remington Laboratory for Specialty Diagnostics, Palo Alto, CA USA; 2 Antios Therapeutics, Mendham, NJ USA; 3 Evidinno Outcomes Research Inc, Vancouver, Canada; 4 Doctor Evidence LLC, Santa Monica, CA USA; 5 Department of General Medicines, Sanofi S.A, Bridgewater, NJ USA; 6 Division of Biologic Sciences, Departments of Pediatrics (Infectious Diseases) and Ophthalmology and Visual Sciences, University of Chicago, Chicago, IL USA

**Keywords:** Spiramycin, Toxoplasmosis, Systemic protozoa, Sequelae, Mortality, Transmission

## Abstract

**Purpose:**

We aimed to investigate the effect of antepartum treatment with spiramycin with or without subsequent pyrimethamine–sulfonamide–folinic acid, compared to no treatment, on the rate of mother-to-child transmission (MTCT) of *Toxoplasma gondii* (*T. gondii*) and incidence/severity of sequelae in the offspring.

**Methods:**

Embase and PubMed were searched for literature on spiramycin in pregnant women suspected/diagnosed with *T. gondii* infection. Meta-analyses were performed using random-effects model.

**Results:**

Thirty-three studies (32 cohorts and 1 cross-sectional study), with a total of 15,406 mothers and 15,250 offspring, were pooled for analyses. The MTCT rate for all treated patients was significantly lower than the untreated [19.5% (95% CI 14–25.5%) versus 50.7% (95% CI 31.2–70%), *p* < 0.001]. The transmission rate in patients on spiramycin monotherapy was also significantly lower than untreated [17.6% (95% CI 9.9–26.8%) versus 50.7% (95% CI 31.2–70%), *p* < 0.001].

**Conclusion:**

Results indicate significant reduction in MTCT rates following spiramycin treatment of suspected/diagnosed maternal *T. gondii* infection.

**Supplementary Information:**

The online version contains supplementary material available at 10.1186/s40001-021-00606-7.

## Introduction

Toxoplasmosis is a parasitic disease caused by *Toxoplasma gondii* (*T. gondii*). It is a neglected disease in some regions of the globe with emerging knowledge and new approaches arising for its diagnosis and treatment. Acute *T. gondii* infection acquired during pregnancy presents a serious risk for potential mother-to-child transmission (MTCT). Fetal infection with *T. gondii* can lead to spontaneous abortion, stillbirth, or serious/severe postnatal sequelae [1–3].

After diagnosis, treatment is often recommended to prevent MTCT [4, 5]. Spiramycin (a macrolide antibiotic) is prescribed when primary maternal infection is diagnosed during gestation in an attempt to prevent MTCT [6, 7]. Spiramycin is not approved in the United States (U.S.), but is accessible through a compassionate use program for toxoplasmosis during pregnancy [8]. The burden of congenital toxoplasmosis may be disproportionately borne by patients of lower socioeconomic strata, and those who lack access to antepartum screening and prevention. However, in the U.S. infection acquired in gestation that places the fetus at risk for congenital infection affects persons of all demographics.

Timing of administration of spiramycin as the initial treatment when primary infection with *T. gondii* occurs during gestation varies by center, depending on when the diagnosis is made between 16 to 32 weeks of gestation (WG) [9–11]. After 14–18 WG and before any fetal infection is documented/diagnosed, additional treatment options include: (a) continuation of spiramycin monotherapy; (b) switching to combination therapy with pyrimethamine–sulfonamide–folinic acid (PSF); (c) PSF alternating or in combination with spiramycin, and/or (d) other experimental treatments that are not standard of care [12]. However, if fetal infection is suspected (based on fetal ultrasound findings suggestive of congenital toxoplasmosis [CT]) or confirmed (with presence of *Toxoplasma* DNA in amniotic fluid by polymerase chain reaction testing), PSF is instituted or continued throughout gestation and the infant’s first year of life [12].

Although the reported effectiveness of spiramycin as antepartum treatment is currently generally accepted, no recent meta-analysis has assessed the efficacy of spiramycin use. Furthermore, reports published between 1999 and 2006 [13–18] cast doubt on the effectiveness of spiramycin, leading a group of investigators to call for randomized placebo-controlled clinical trials [13, 18] and others to advocate for the discontinuation of systematic antepartum screening and treatment programs [19, 20]. During this period of time, diagnosing or treating congenital toxoplasmosis at all was controversial in some settings. Thus, especially in light of the recent global initiatives to prevent this infection it is critical to evaluate the evidence for the efficacy and role of spiramycin.

To address differences in approach concerning prenatal screening, treatment, and the use of spiramycin, we conducted meta-analyses of literature published as of August 2017 to investigate the effect of antepartum treatment with spiramycin monotherapy or spiramycin combined/alternated with PSF compared to no treatment on the rate of MTCT and the incidence/severity of sequelae in the offspring.

## Materials and methods

### Literature search and study selection

A focused search on the treatment of *T. gondii* infection during pregnancy was conducted on Embase and PubMed using keywords: “Spiramycin AND Pregnancy” and “Spiramycin AND Toxoplasmosis”. The search results and citation lists of eligible publications were reviewed for inclusion. Studies that were not published in English; did not report on MTCT rate or sequelae; compared various diagnostic methods; or focused on etiology, pathology, pathogenesis, or epidemiology of toxoplasmosis were excluded. Results were reported according to the Preferred Reporting Items for Systematic Reviews and Meta-Analyses (PRISMA) guidelines [21].

### Data configuration

Data were extracted by investigators and entered into Doctor Evidence LLC’s DOC Data version 2.0 software platform. The primary outcome was MTCT rate, and secondary outcomes were incidence/severity of sequelae in infected fetuses/infants. Sequelae were defined based on previous studies [22, 23]. Definition of outcomes and denominators are provided in Table 1 and Additional file 1: Tables S1, S2. The Newcastle–Ottawa Scale [24] was used to assess the quality of the included observational studies (detailed in Additional file 1: Table S4).

**Table 1:** Outcome definitions

Outcome	Definition	Denominators
Mother-to-child transmission (MTCT)	Transmission of *Toxoplasma gondii* from mother-to-child	All infected mothers
Mortality (pre- and post-natal) excluding elective terminations^a^	Includes toxoplasmosis-associated spontaneous abortions, stillbirths, and postnatal infant deaths	All infected fetuses/children
All serious/severe sequelae and all mortality (pre- and post-natal)	Includes serious/severe sequelae and all mortality (all toxoplasmosis-associated terminations, miscarriages, stillbirths, and infant deaths within the first year of life)	All infected mothers All infected fetuses/children
All mild/moderate/severe sequelae and infant mortality (postnatal)	Includes postnatal mild/moderate/severe sequelae and toxoplasmosis-associated infant mortality; excludes terminations and stillbirths	All infected live-born children

^a^Elective terminations are terminations of pregnancy that have been decided upon after suspected or confirmed diagnosis of toxoplasmosis

### Statistical analyses

#### Meta-analyses

Meta-analyses were conducted to determine pooled estimates. Outcomes were pooled based on availability across the included studies. Analyses were performed for the following treatment groups: untreated, spiramycin monotherapy, and spiramycin monotherapy or spiramycin monotherapy followed by spiramycin in combination or alternating with PSF, heretofore referred to as “spiramycin and/or PSF”.

A random-effects model by DerSimonian and Laird 1986 [25] was used to derive composite odds ratio of outcomes for each group of interest using the Freeman–Tukey double arcsine method [26, 27]. Random-effects models took into account both within-study and between-study variability. The alpha value of 0.05 was considered statistically significant. I^2^ was used to assess heterogeneity. All analyses were performed using R version 3.0.3 (http://www.r-project.org/) by using R package ‘metafor’.

#### Subgroup/sensitivity analyses

Subgroup analyses were conducted for each analysis set by trimester, publication date (< 1999, 1999–2006, > 2006), and follow-up timepoint. All comparisons were naïve. Findings are presented in Additional file 1: Figs. S13–S76.

Sensitivity analyses were performed on studies that met all of the following criteria: (1) regular screening (e.g., monthly, bimonthly, by trimester, or quarterly); (2) spiramycin dosage between 2–3 g/day used at any time during the pregnancy; (3) availability of information on transmission rate and/or infant sequelae and its severity; (4) availability of postnatal follow-up data, and (5) studies with > 100 patients [28]. Several studies were excluded from sensitivity analyses due to the following [29]: in many studies conducted during a certain period (1999–2006) [13, 14, 16], a significant effect of prenatal treatment on the risk of vertical transmission and clinical signs of CT was not detected because very few untreated women were included in their analyses, most untreated women were infected during the third trimester, and severe cases were excluded [30, 31]. Findings are presented in Additional file 1: Figs. S77–S108.

## Results

**Fig. 1:**
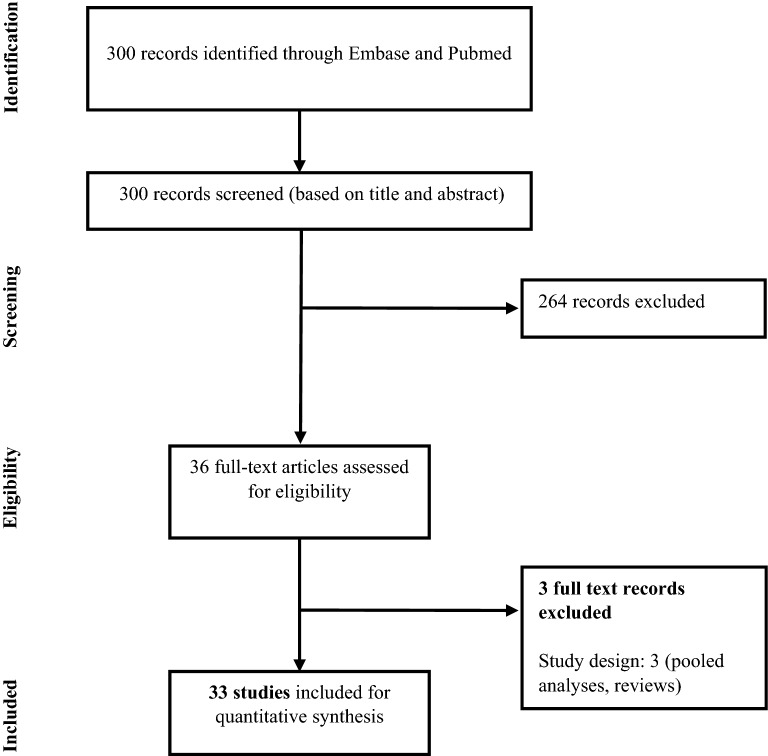
PRISMA study diagram

Out of 300 publications reviewed, 33 studies (32 cohorts and 1 cross-sectional study) comprising a total of 15,406 mothers and 15,250 offspring were eligible for inclusion (Fig. 1). Two studies [23, 32] did not report the number of mothers, and the number of offspring was not reported in two studies [33, 34]. Fifteen studies met the criteria for sensitivity analyses (Additional file 1: Table S3). Additional details on study and patient characteristics and the quality assessments are provided in the Additional file 1: Tables S3, S4.

The main analysis was performed by pooling all 33 studies, despite differences across the selected studies with respect to the patient population (country/region, timing of primary infection in gestational stage at which primoinfection incurred), screening/diagnostic methods (with or without regular prenatal screening and polymerase chain reaction [PCR] on amniotic fluid), prenatal treatments (dosage and timing of spiramycin as first treatment, follow-up treatment regimen involving PSF or other), and year of study (1974–2016). Studies were heterogeneous due to the differences in outcome definition, published period, methods of treatment, and medical practices in different countries. Additional file 1: Tables S5, S6 summarize all the results of the meta-analyses.

### Mother-to-child transmission

**Fig. 2:**
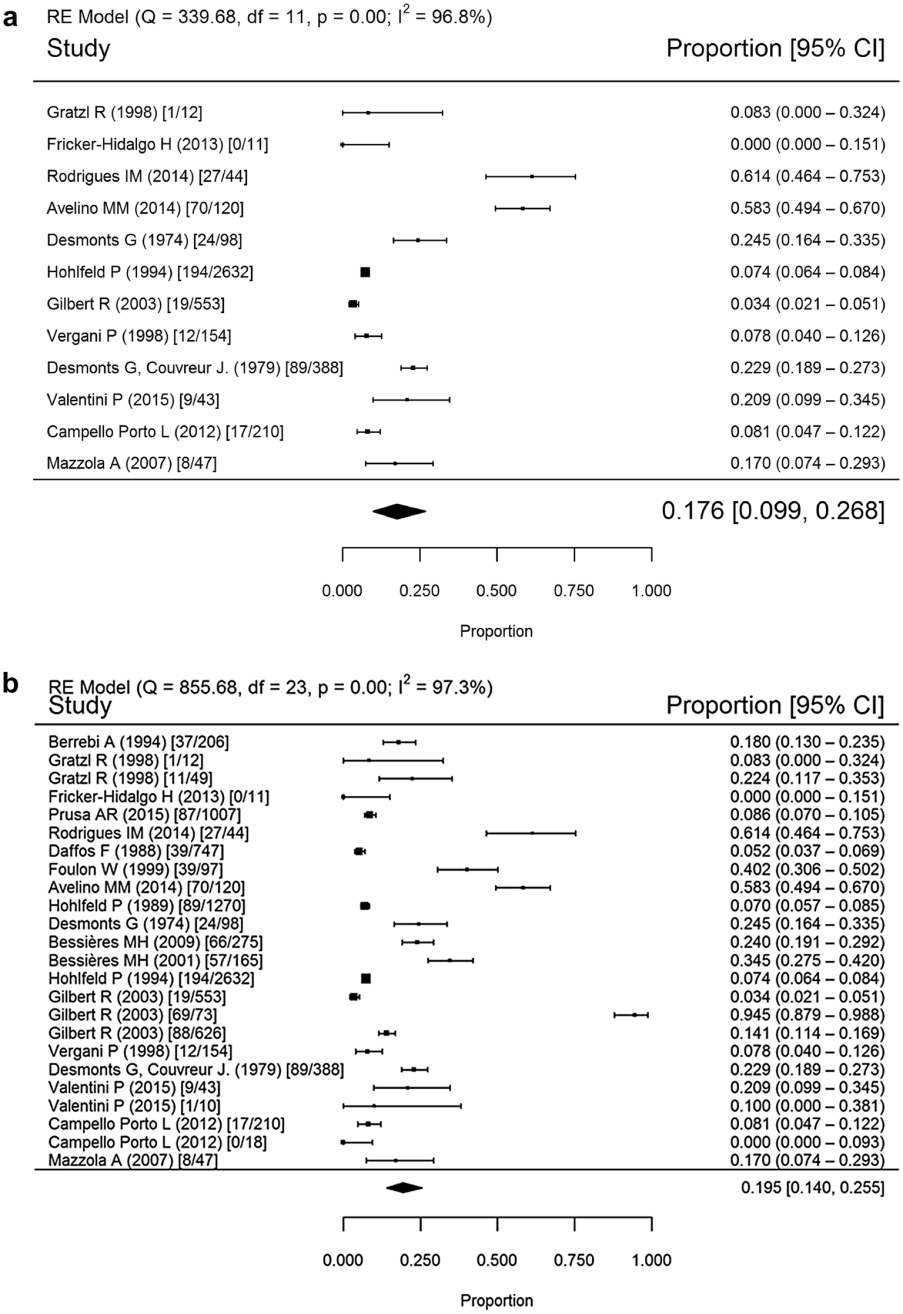

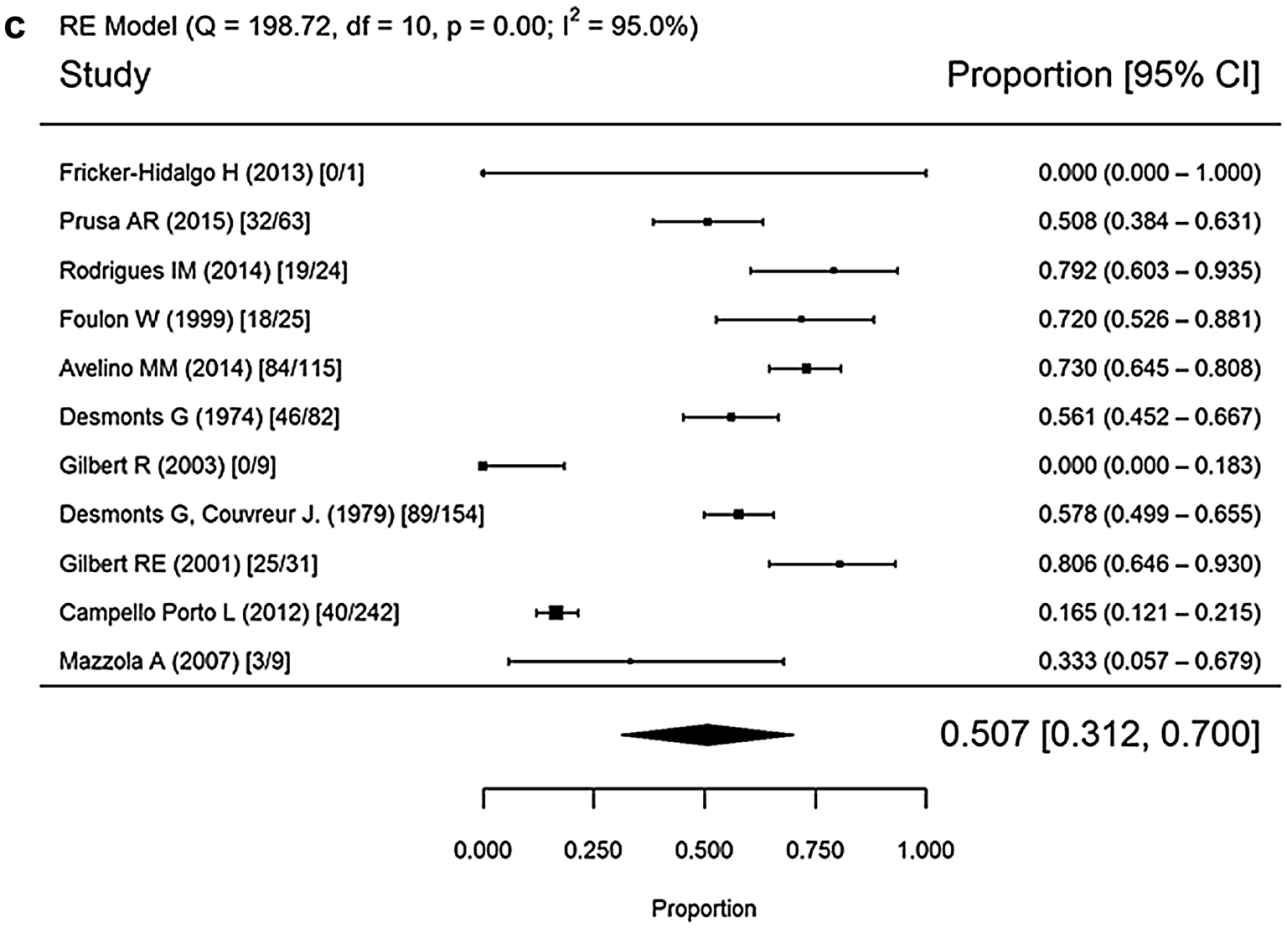
Forest plot of maternal to child transmission rate by treatment, 1974–2016. **a** Treatment with spiramycin monotherapy; **b** treatment with spiramycin and/or PSF treatment; **c** untreated. Random effects (RE), heterogeneity test (I^2^), degrees of freedom (df), confidence interval (CI)

Twenty studies conducted in 1974–2016 were used to calculate MTCT rates (Fig. 2). The mean rates were 17.6% (95% confidence interval [CI] 9.9–26.8%) for the spiramycin monotherapy group, 19.5% (95% CI 14–25.5%) for the spiramycin and/or PSF group, and 50.7% (95% CI 31.2–70%) for the untreated group (*p* < 0.001 for both treated versus untreated comparisons).

### Post-transmission outcomes

Nine studies with up to 1-year follow-up conducted in 1974–2016 were used to calculate rates of infected offspring mortality due to CT but excluding elective terminations of pregnancy due to fetal infection (Additional file 1: Figs. S1, S2). The mean rates were similar in all three groups.

Nine studies with up to 1-year follow-up conducted in 1974–2016 were used to calculate serious/severe sequelae and all offspring mortality rates using the infected mothers as the denominator (Additional file 1: Figs. S3, S4). The mean rates were 1.0% (95% CI 0–3.5%) for the monotherapy group, 1.2% (95% CI 0–3.7%) for spiramycin and/or PSF group, and 21.4% (95% CI 2.4–49.7%) for the untreated group (*p* < 0.001 for both treated versus untreated comparisons).

Ten studies with up to 1-year follow-up conducted in 1974–2016 were used to calculate serious/severe sequelae and all offspring mortality rates using the infected fetuses as the denominator (Additional file 1: Figs. S5, S6). The mean rates were 4.7% (95% CI 0–22.9%) for the spiramycin monotherapy group, 7.5% (95% CI 1.2–17.5%) for the spiramycin and/or PSF group, and 36.5% (95% CI 5.1–75.2%) for the untreated group (*p* = 0.040 spiramycin monotherapy versus untreated, *p* = 0.004 spiramycin and/or PSF versus untreated).

Twelve studies with up to 1-year follow-up conducted in 1974–2016 were used to calculate mild/moderate/severe sequelae and postnatal infant mortality rates (Additional file 1: Figs. S7, S8). The mean rates were 32.6% (95% CI 16.4–51.1%) for the monotherapy group, 21.6% (95% CI 10–35.6%) for the spiramycin and/or PSF group, and 65.8% (95% CI 27.6–95.9%) for the untreated group (*p* < 0.001 for both treated versus untreated comparisons).

Details on subclinical disease and chorioretinitis are provided in Additional file 1: Figs. S9–S12. From studies published in 1974–2016 with up to 1-year follow-up, the mean rates of subclinical disease in the monotherapy group (63.4% [95% CI 43.3–81.3%], *p* = 0.003) and in the group treated with spiramycin and/or PSF (67.5% [95% CI 52.9–80.6%], *p* < 0.001) were significantly higher than in the untreated group (43.4% [95% CI 9.4–81.3%]). The average rate of chorioretinitis between the untreated group and the group treated only with spiramycin or the group treated with spiramycin and/or PSF were similar.

### Subgroup analyses

MTCT was lower in the spiramycin monotherapy and the spiramycin and/or PSF groups compared to the untreated groups across all trimesters. The rates of all serious/severe sequelae plus mortality tended to be significantly lower in all treated versus untreated groups in both infected pregnant women and in offspring across all publication date periods.

## Discussion

The results of these meta-analyses corroborate previous findings suggesting the benefit of antepartum spiramycin monotherapy or spiramycin and/or PSF in decreasing the rate of MTCT compared to no treatment. A previous meta-analysis using nine studies also found lower rates of MTCT for spiramycin monotherapy or spiramycin and/or PSF [35]. We present results from a larger evidence base of 33 studies, and report on more clinical post-transmission outcomes such as mortality due to congenital toxoplasmosis, mild/moderate or severe/serious sequelae, and all-cause fetal mortality.

During preliminary searches, we found a meta-analysis published in 2007 that further supported the need for prompt detection and treatment. The systematic review on congenital toxoplasmosis (SYROCOT) compared the effect of earlier versus later prenatal treatment (adjusted for gestational age at seroconversion) and found evidence indicating an increased risk of MTCT when prenatal treatment initiation was delayed after maternal seroconversion [18].

Although not included in this review as it was published after the search date, a randomized controlled open-label trial conducted in France (TOXOGEST) investigated spiramycin therapy until amniocentesis at 18 weeks, versus initiating pyrimethamine–sulfadiazine–folinic acid as early as 14 weeks to prevent MTCT prior to amniocentesis [36]. Despite the small differences between treatment groups, the results suggest that in the window between 14 and 17 WG, there is an opportunity to reduce the incidence of severe disease in the fetus with use of pyrimethamine–sulfadiazine–folinic acid; however, it is accompanied by a slight increase in risk of severe maternal hypersensitivity. Additional studies might help discriminate the pregnancies that would benefit from earlier diagnosis and initiation of pyrimethamine–sulfadiazine–folinic acid.

In France, TOXOGEST has begun to shift the treatment paradigm towards offering pyrimethamine and sulfadiazine as early as 14 WG. Because of diminished severe neurologic effects in a small number of fetuses treated from 14 to 18 WG, this approach has been used for pregnant women who seroconvert in the first trimester and thereafter [36, 37]. Although the approach for treatment after 14 WG is changing, spiramycin remains the standard of care for infections prior to 14 weeks. Other practices offer spiramycin upfront after 14 WG until amniocentesis is performed at 17 to 18 weeks gestation. Since pyrimethamine and sulfadiazine can lead to negative PCR results [22], interpretation of amniocentesis and decisions about subsequent medical care may be complicated when a pregnant woman has received a pyrimethamine sulfadiazine regimen beforehand. While developing new medicines [38–40] may have less teratogenicity in the first trimester than pyrimethamine, and vaccination [41] may be another cost-effective preventative approach, at present gestational screening and spiramycin-based treatment for seroconverting women in the first 14 weeks of gestation protect against congenital toxoplasmosis.

Experiences from many countries have shown that the risk of MTCT and consequential serious/severe sequelae can be decreased with regular screening and prompt antepartum treatment. Austria has for many years utilized an approach, as described above, with favorable outcomes, similar to the current new approach in some clinical practices in France [42]. However, in the U.S., these systems are not generally available, and infected children manifest more severe disease and poorer outcomes [43]. A recent effort has begun to address this unmet need through the development of inexpensive point-of-care testing monthly for pregnant women, and prompt treatment of the pregnant woman [44–46].

Cost–benefit analyses have demonstrated that when costs are constrained to reasonable levels, as in Austria [10] or France, screening and treatment can dramatically improve outcomes and quality of life by improving sight, cognition and motor functions [47–49]. Furthermore, the Austrian cost–benefit analysis utilized mathematical modeling to demonstrate that the screening program’s benefits were 14 times its cost; food safety education reduced maternal infection; early treatment reduced MTCT rates from 51 to 11%; education, testing, and early treatment reduced cases of CT from 78 to 1 per 10,000; infected children did not have any profound, lifelong injuries and could enter the workforce; and although screening programs cost €1.9 million per year, the program saved €450 million over 17 years [10]. In the U.S., mathematical modeling indicated that monthly prenatal screening is cost-saving when tests are $12 and congenital infection rates are above 1/10,000 live births [49]. In developing countries with inconsistent prenatal care, the potential spillover benefits of a nationally mandated screening with inexpensive point-of-care tests ($4–8) are extensive as it promotes routine prenatal visits for other health risks [44]. When toxoplasmosis testing is combined with testing for other congenital infections, the potential benefits and cost savings are even larger [50].

In our meta-analyses, no significant difference was found in mortality, excluding elective terminations of pregnancy due to fetal infection. However, rates of MTCT, serious/severe sequelae and mortality combined, and mild/moderate/severe sequelae and infant mortality combined were significantly reduced when mothers received spiramycin monotherapy or spiramycin and/or PSF compared to the untreated group. More studies are required to determine how the trimester at which infection occurred affected the type of adverse outcomes.

### Clinical significance

The findings of this review are an important addition to the current evidence base for antepartum treatment of toxoplasmosis. Some reports published between 1999 and 2006 had raised doubts for some investigators about the effectiveness of spiramycin-containing treatment regimens [13–18]. This led some investigators to advocate for discontinuation of systematic screening and treatment programs for toxoplasmosis [19, 20], and others to call for prospective randomized placebo-controlled clinical trials that proved efficacy of treating this infection [13, 18]. The results of our meta-analyses therefore help address these differences in approach concerning prenatal screening, treatment, and regimens including the use of spiramycin. Additionally, we believe that this review paves the way toward systematic antepartum screening and prompt treatment for patients with *T. gondii* infection acquired during gestation in parts of the world where this does not yet occur. This facilitates treatment of pregnant women to prevent congenital toxoplasmosis and will allow clinicians to better understand all treatment options at this critical time.

The severity of sequelae tend to increase with infection during earlier stages of pregnancy [51]; however, signs may fail to be captured by studies with insufficient follow-up time [52]. Although only women who are first infected during their pregnancy are thought to be at risk of MTCT, current methods using IgM and IgG cannot differentiate between primary and secondary infection if performed late in pregnancy [53]. Additionally, toxoplasmosis may also present as asymptomatic or flu-like and thus delay testing [53]. Early detection is essential to preventing severe sequelae because *T. gondii* may hide within immune cells and later cause systemic disease [54]. Thus, a wide range of clinical manifestations associated with CT could be prevented by prompt treatment [54].

Although not evaluated in this study, environmental factors should also be considered when evaluating the antepartum use of spiramycin. In one South American study, more virulent strains of *T. gondii* caused severe infections in 40% of the infants [55], emphasizing a need for accessible treatment that could be met through spiramycin monotherapy [56]. Cost-effectiveness is particularly important as toxoplasmosis represents a high burden of disease in rural regions, and can be acquired through contaminated soil, water, and meat [57]. Distribution of spiramycin alone, rather than combination therapies, could be more cost-effective where access to medical supplies is limited [56].

This study comes with its limitations. First, it is not as comprehensive as a systematic literature review, and therefore, may not have identified all studies that fulfilled selection criteria. Papers not published in English were excluded, leading to a potential risk of bias and failure to capture spiramycin use in other countries. Studies not yet indexed in databases or published close to the search date were not captured in this paper, which represents a snapshot of an evolving evidence base. In our meta-analyses, we used pooled patients who were untreated throughout pregnancy as historical controls, but the paucity of studies reporting on untreated patients limited sample size. Additionally, not all planned analyses could be performed due to limited data availability, especially for post-transmission outcome endpoints. Prenatal treatment with any drug was preferable to none in reducing MTCT and sequelae. Though treatment with spiramycin was not directly compared to spiramycin and/or PSF via statistical tests, spiramycin monotherapy tended to reduce adverse outcomes at rates similar to the alternative treatment. Significant heterogeneity existed among the studies, due to different *T. gondii* strains, methods of treatment, and potentially the populations’ socioeconomic status, which may have influenced the results of the meta-analyses. Moreover, all analyses were naïve with no adjustment for gestational age of infection and time to start of treatment. An additional limitation of these meta-analyses is the absence of randomized controlled trials. Nonetheless, the difference in treatment outcomes for mortality and sequelae between spiramycin monotherapy or spiramycin and/or PSF compared to no treatment is robust.

### Conclusions

The results of these meta-analyses support the effectiveness of spiramycin monotherapy and spiramycin and PSF treatments during gestation for the prevention of MTCT of *T. gondii* and fetal mild-to-severe sequelae. Placebo-controlled randomized clinical trials for this purpose are lacking, but are deemed unethical. Spiramycin should be instituted as soon as possible to any pregnant woman diagnosed with acute infection during the 1st and early 2nd trimester to reduce MTCT, followed by PSF if fetal infection is suspected or confirmed. Since acute *T. gondii* infection during pregnancy can occur in the absence of symptoms and epidemiologic risk factors, systematic screening is the only approach that would capture all maternal infections and facilitate the early institution of spiramycin and/or PSF during gestation.

## Supplementary Information

**Additional file 1.** Contains definition of outcomes and denominators; additional details on study characteristics, patient characteristics, and quality assessments; post-transmission outcomes; and all subgroup/sensitivity analyses.

## Data Availability

All data generated or analyzed during this study are included in this published article and its supplementary information files.
