# Supplementary material for: A fresh look at the role of spiramycin in preventing a neglected disease: meta-analyses of observational studies
Source: Eur J Med Res. 2021 Dec 11;26:143. doi: 10.1186/s40001-021-00606-7 (PMC8665510; doi:10.1186/s40001-021-00606-7)
Supplement: Supplementary file 1 — Additional file 1. Contains definition of outcomes and denominators; additional details on study characteristics, patient characteristics, and quality assessments; post-transmission outcomes; and all subgroup/sensitivity analyses. [file 40001_2021_606_MOESM1_ESM.docx]

Supplementary Information

**A Fresh Look at the Role of Spiramycin in Preventing a Neglected Disease: Meta-analyses of Observational Studies**

Jose G. Montoya, MD^1^, Katherine Laessig, MD^2^, Mir Sohail Fazeli, MD, PhD^3,^*, Gaye Siliman, MSc^4^; Sophie S. Yoon, MPH^4^, Elizabeth Drake-Shanahan, MSc^5^; Chengyue Zhu, PhD^5^; Akbar Akbary, MD^5^, Rima McLeod, MD^6,^*

1 Jack S. Remington Laboratory for Specialty Diagnostics, Palo Alto, CA, USA

2 Antios Therapeutics, Mendham, NJ, USA

3 Evidinno Outcomes Research Inc., Vancouver, Canada

4 Doctor Evidence LLC, Santa Monica, CA, USA; Evidinno Outcomes Research Inc., Vancouver, Canada

5 Department of General Medicines, Sanofi S.A., Bridgewater, NJ, USA

6 Division of Biologic Sciences, Departments of Pediatrics (Infectious Diseases) and Ophthalmology and Visual Sciences, University of Chicago, Chicago, IL, USA

* Correspondence: mfazeli@evidinno.com (S.F.); Tel.: 778-772-4030 (S.F.) rmcleod@uchicago.edu (R.M.); Tel.: 773-834-4130

# Figures

# Mortality due to CT Excluding Elective Terminations due to Fetal Infection

1. Mortality Excluding Elective Terminations, G1 vs Untreated, 1974-2016 – Up to 1 year


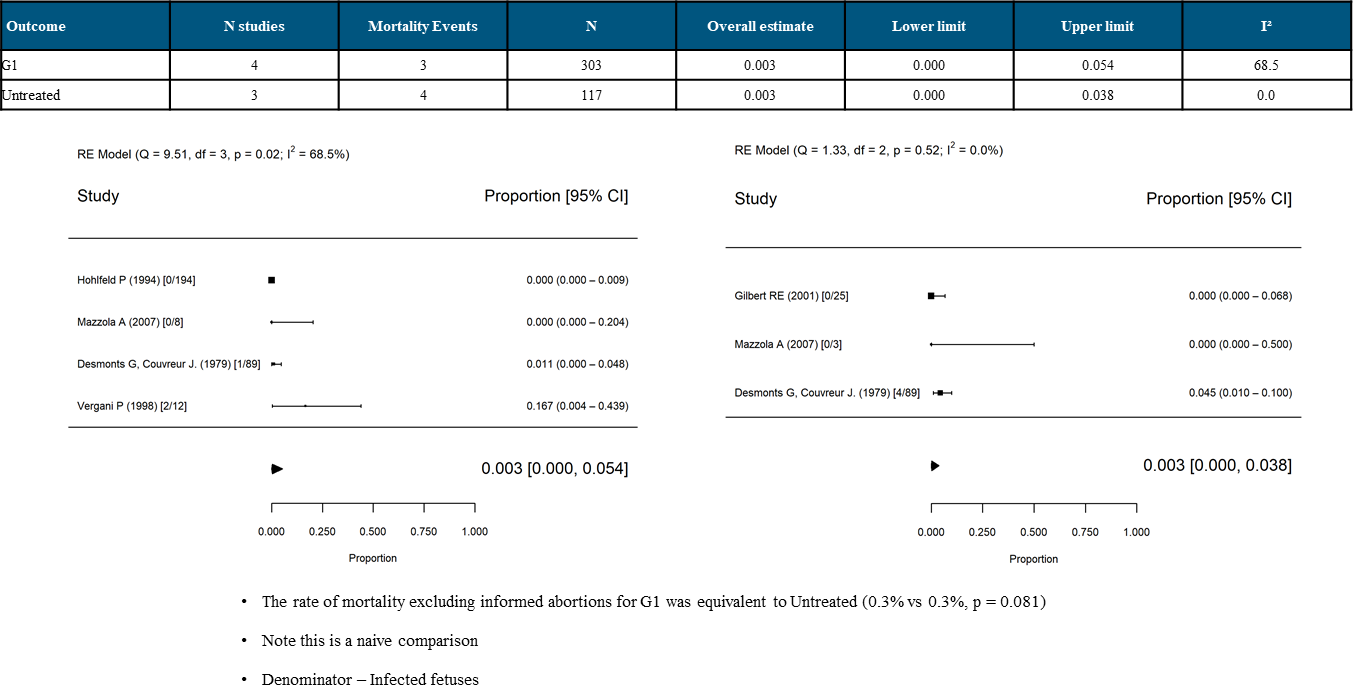


1. Mortality Excluding Elective Terminations, G1+G2 vs Untreated, 1974-2016 – Up to 1 year


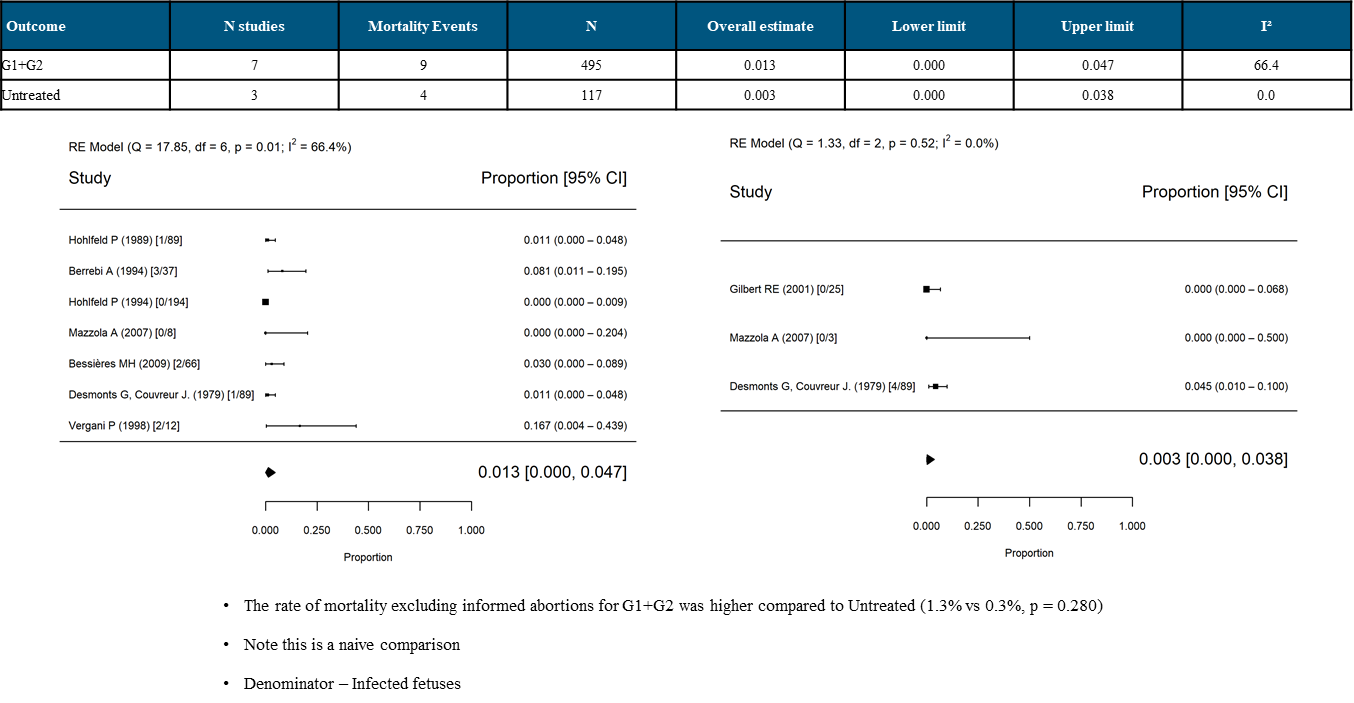


## Serious/Severe Sequelae and All Mortality: Infected Pregnant Women

1. All Serious/Severe Sequelae and All Mortality, G1 vs Untreated, 1974-2016 – Up to 1 year


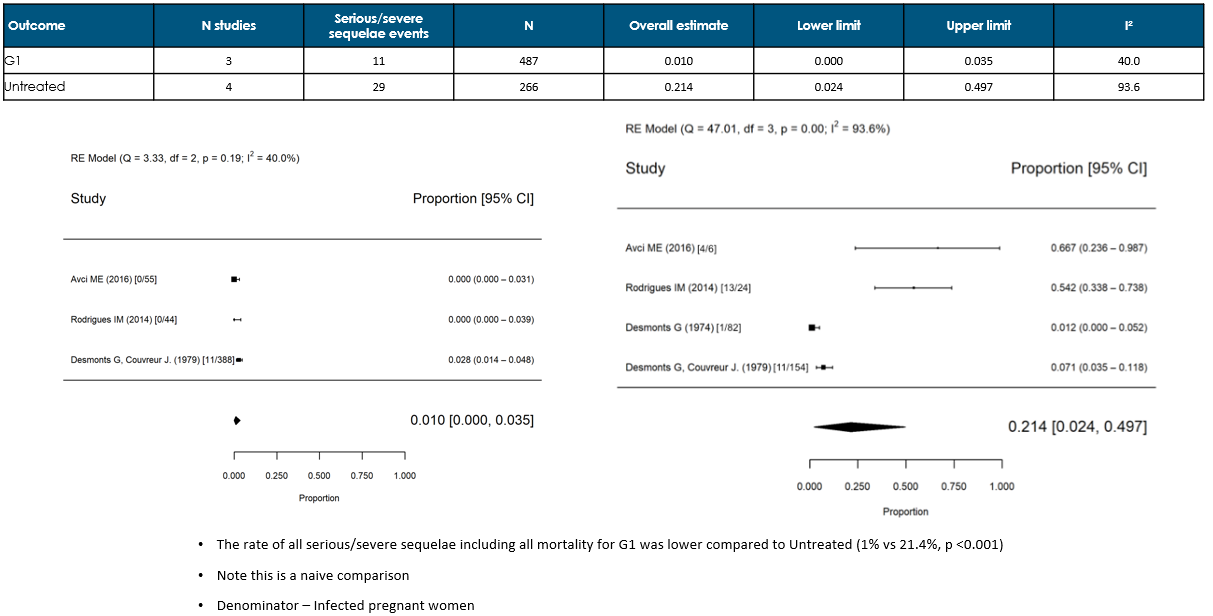


1. All Serious/Severe Sequelae and All Mortality, G1+G2 vs Untreated, 1974-2016 – Up to 1 year


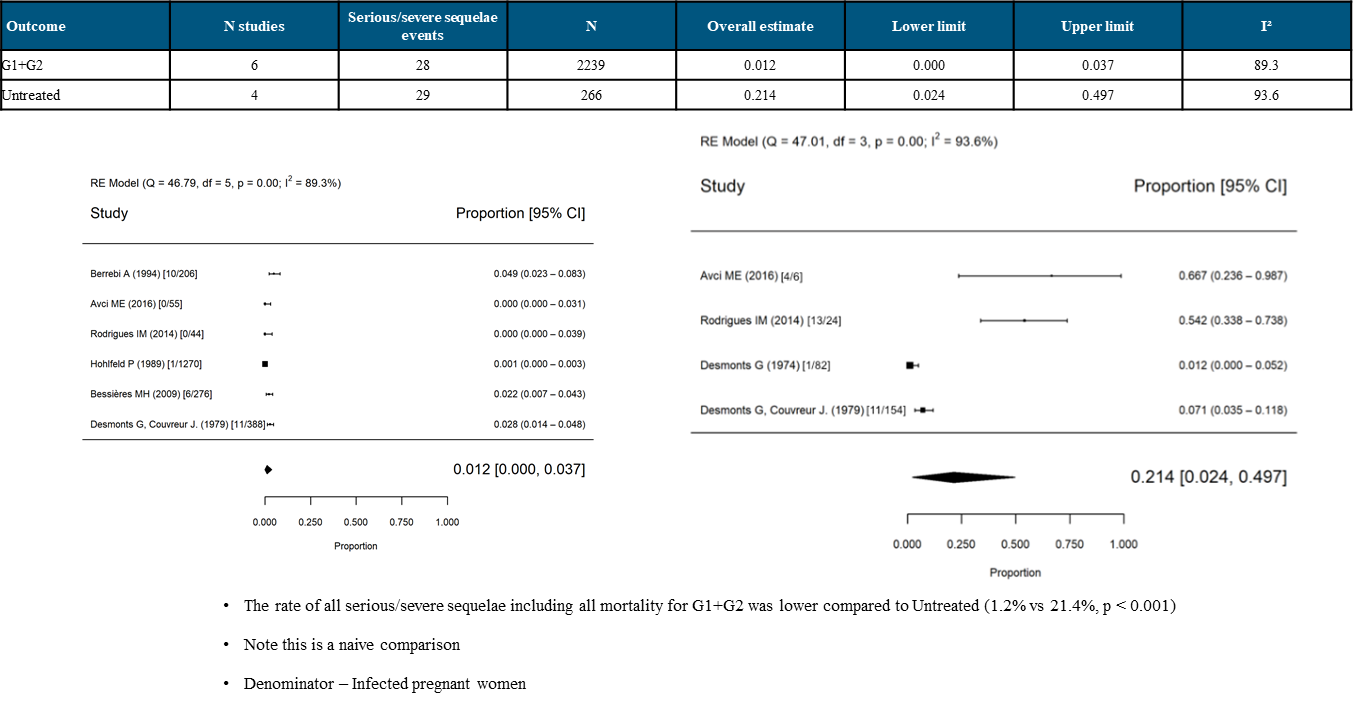


## Serious/Severe Sequelae and All Mortality: Infected Fetuses

1. All Serious/Severe Sequelae and All Mortality, G1 vs Untreated, 1974-2016 – Up to 1 year


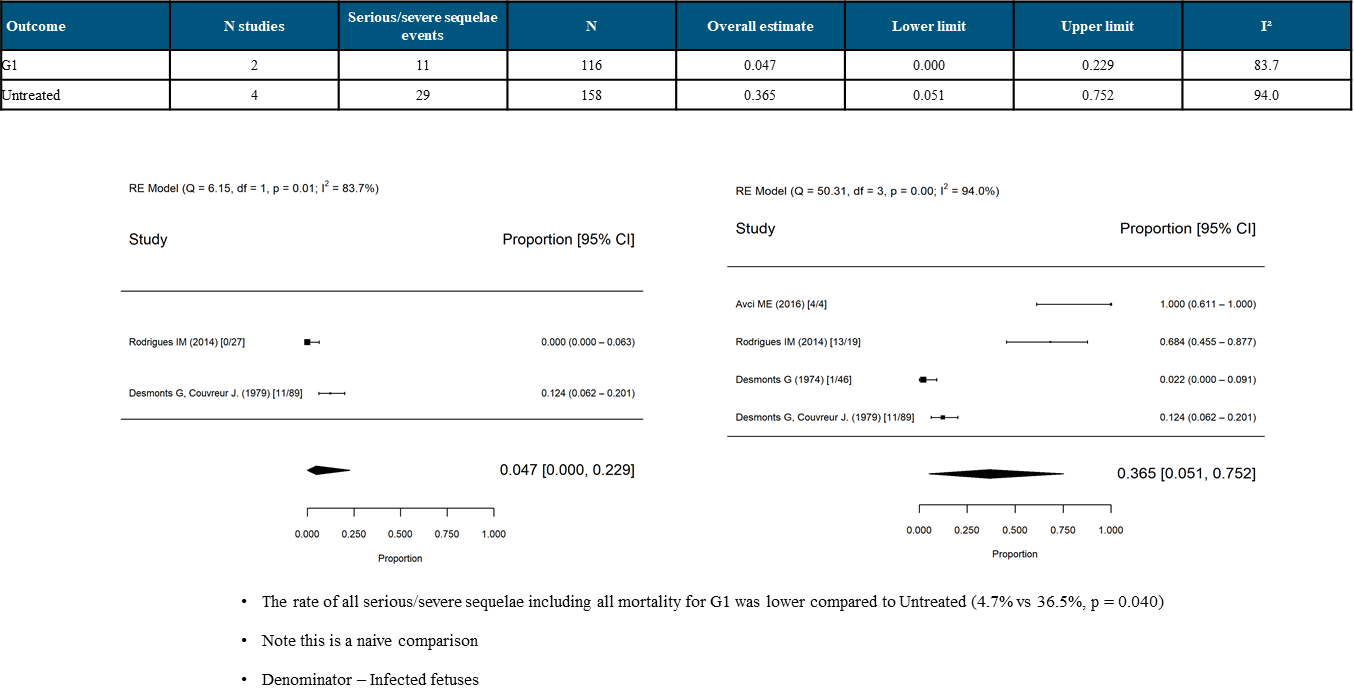


1. All Serious/Severe Sequelae and All Mortality, G1+G2 vs Untreated, 1974-2016 – Up to 1 year


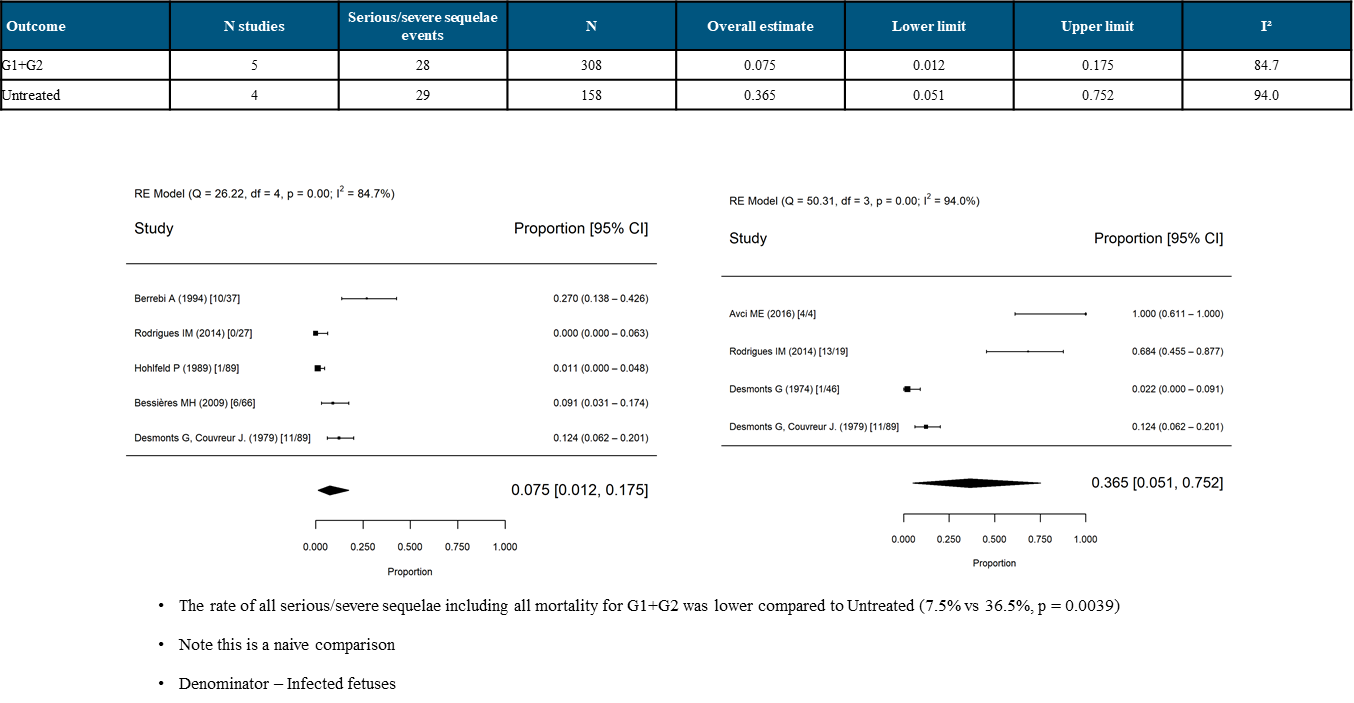


## Mild/Moderate/Severe Sequelae and Infant Mortality

1. All Mild/Moderate/Severe Sequelae and Infant Mortality (Postnatal), G1 vs Untreated, 1974-2016 – Up to 1 year


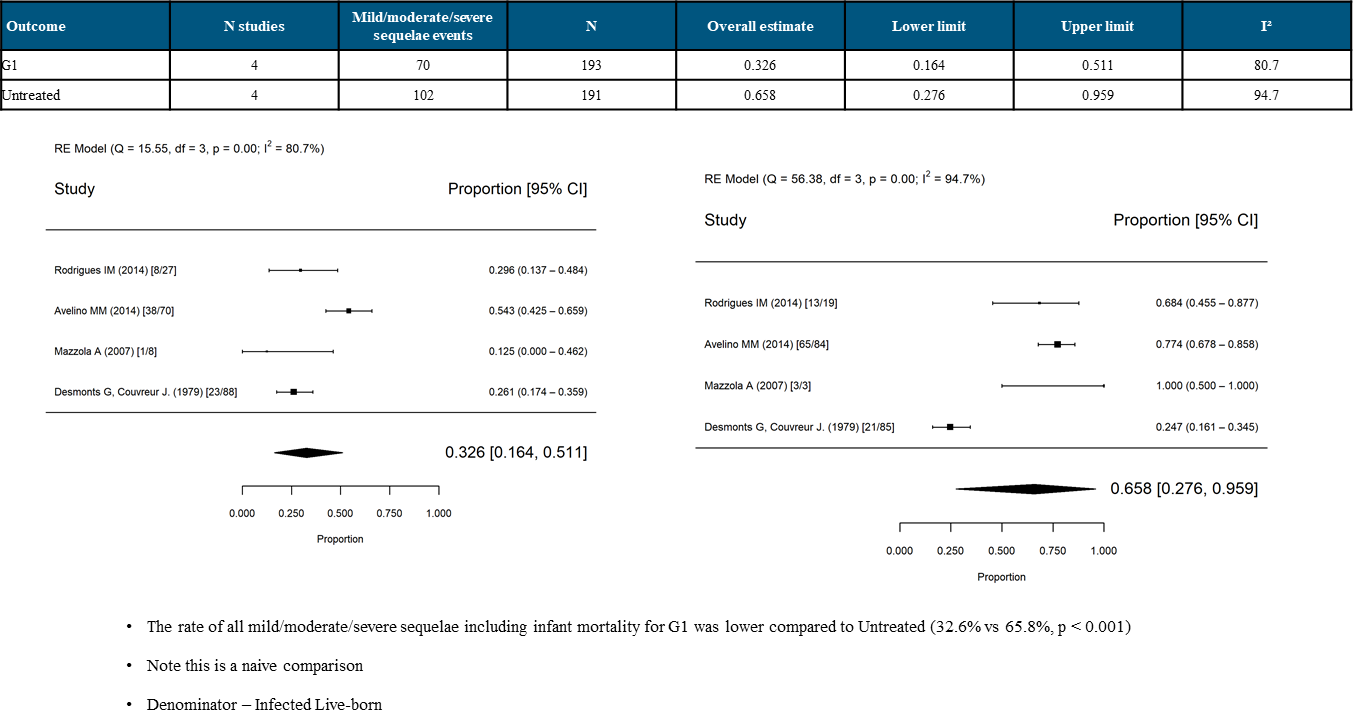


1. All Mild/Moderate/Severe Sequelae and Infant Mortality (Postnatal), G1+G2 vs Untreated, 1974-2016 – Up to 1 year


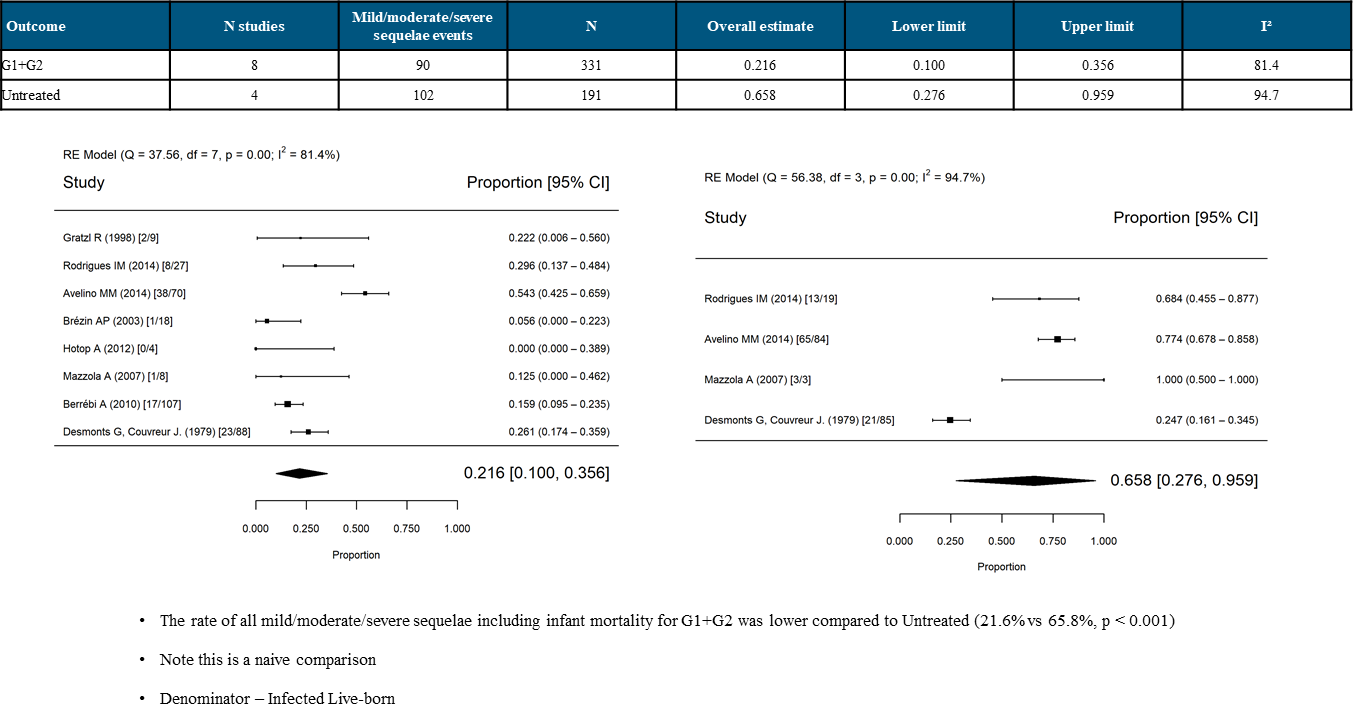


## Subclinical Disease

Five studies with up to one-year follow-up conducted in 1979-2016 were used to calculate subclinical disease rates using the infected live-born children as the denominator (**Figure S9-S10**). The mean rates were 63.4% (95% CI: 43.3-81.3%) for the spiramycin monotherapy group, 67.5% (95% CI: 52.9%-80.6%) for the spiramycin and/or PSF group, and 43.4% (95% CI: 9.4-81.3%) for the untreated group (p=0.003 spiramycin monotherapy versus untreated, p<0.001 spiramycin and/or PSF versus untreated).

1. Subclinical Disease, G1 vs Untreated, 1974-2016 – Up to 1 year


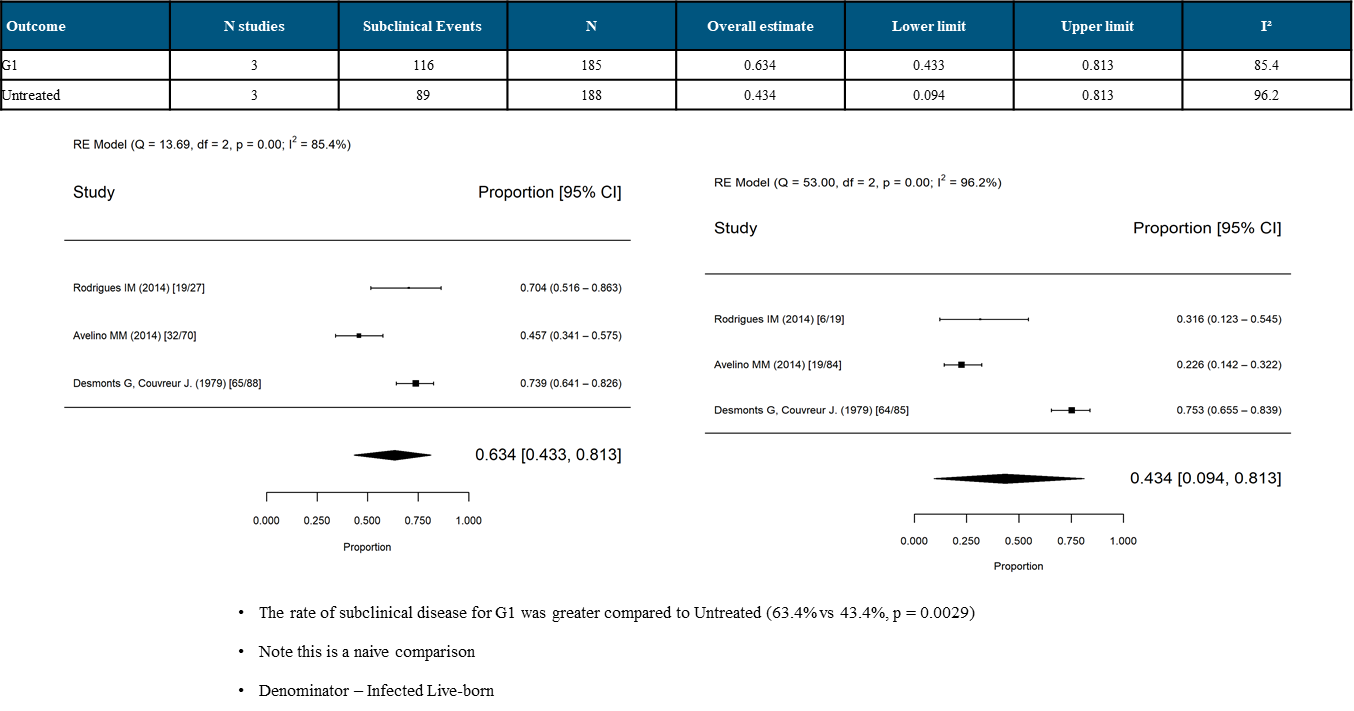


1. Subclinical Disease, G1+G2 vs Untreated, 1974-2016 – Up to 1 year


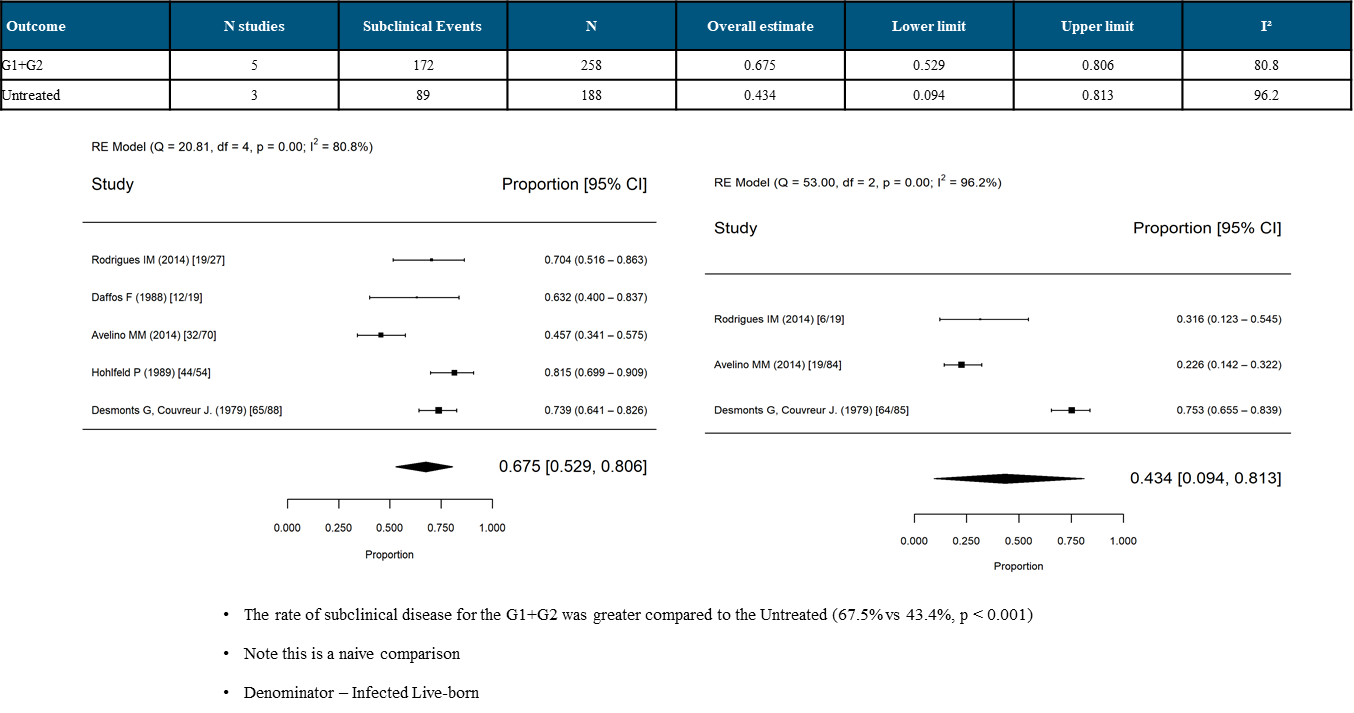


## Chorioretinitis

Two studies with up to one-year follow-up conducted in 1974-2016 were used to calculate chorioretinitis rates using the infected live-born children as the denominator (**Figure S11-S12**). The mean rates were 3.7% (95% CI: 0-15.2%) for the spiramycin monotherapy group, 10.3% (95% CI: 1.7-23.9%) for the spiramycin and/or PSF group, and 15.8% (95% CI: 2.3-36.2%) for the untreated group (p-values were not statistically significant for both treated versus untreated comparisons).

1. Chorioretinitis, G1 vs Untreated, 1974-2016 – Up to 1 year


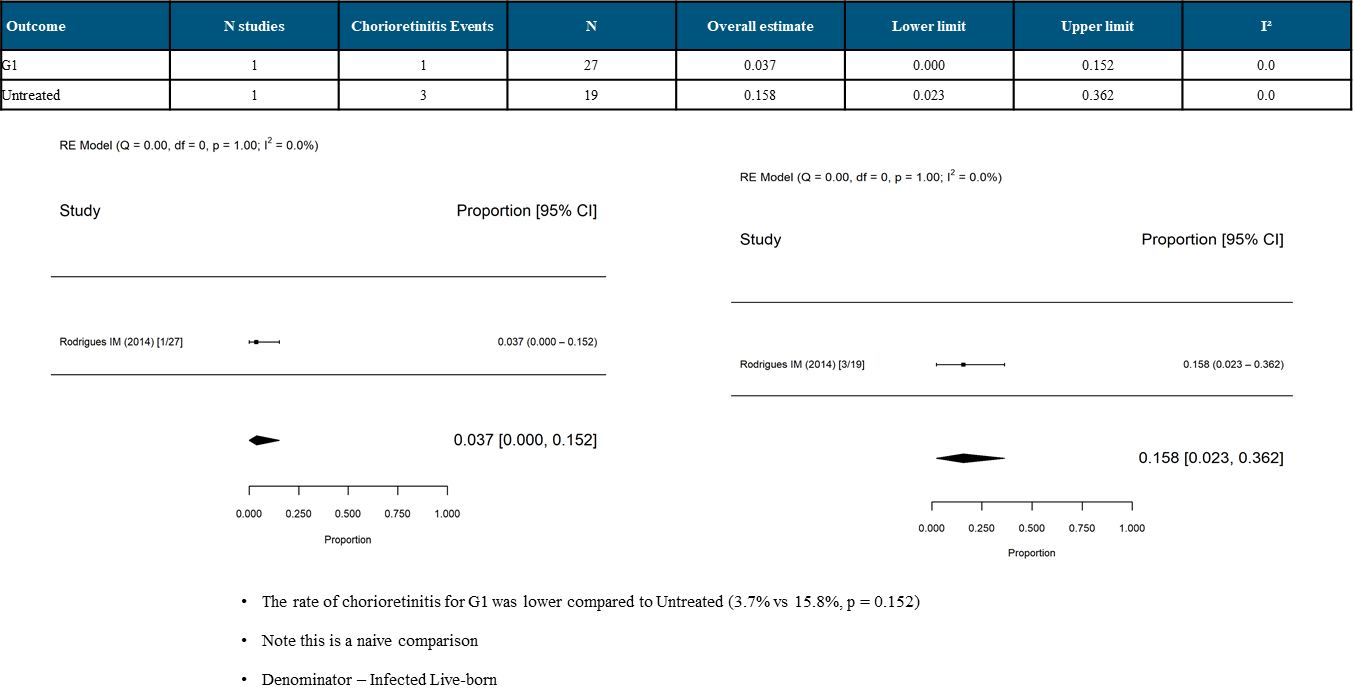


1. Chorioretinitis, G1+G2 vs Untreated, 1974-2016 – Up to 1 year


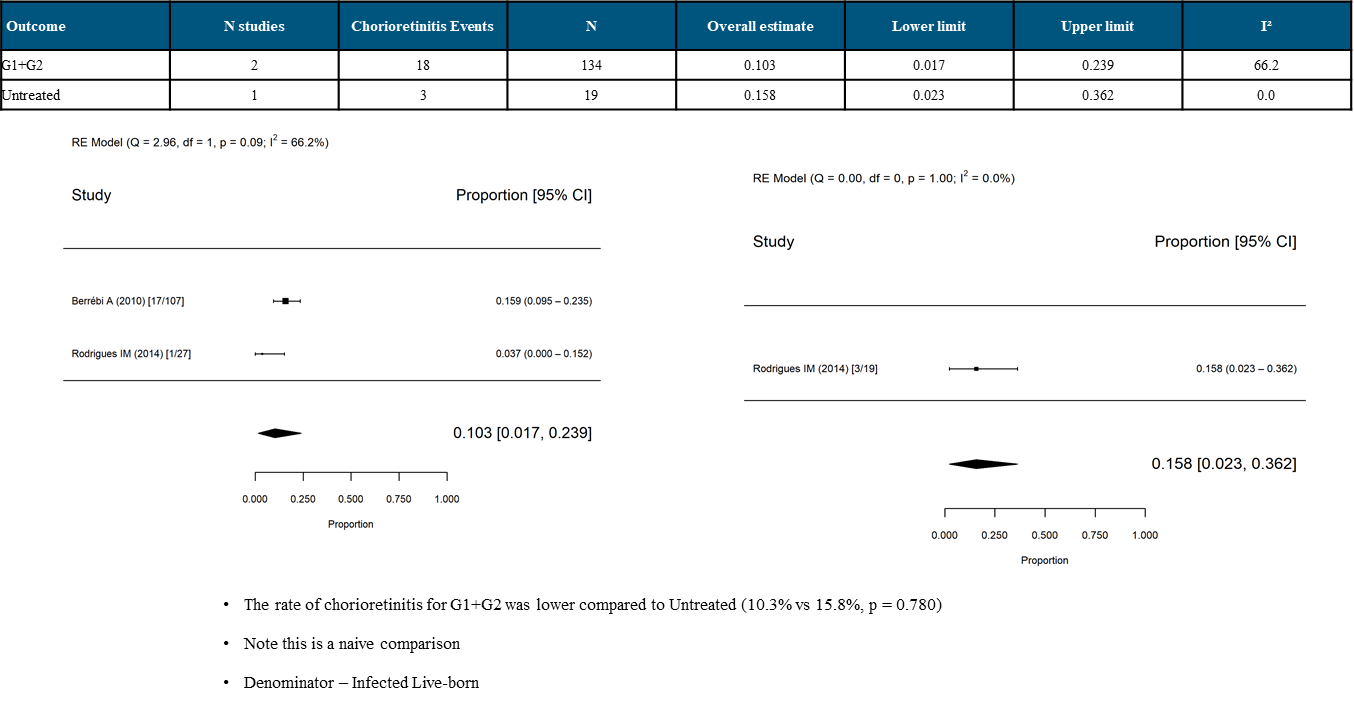


## Subgroup Analyses

### MTCT - Subgroup Analyses

1. MTCT, Spiramycin Monotherapy (G1) vs Untreated, Before 1999


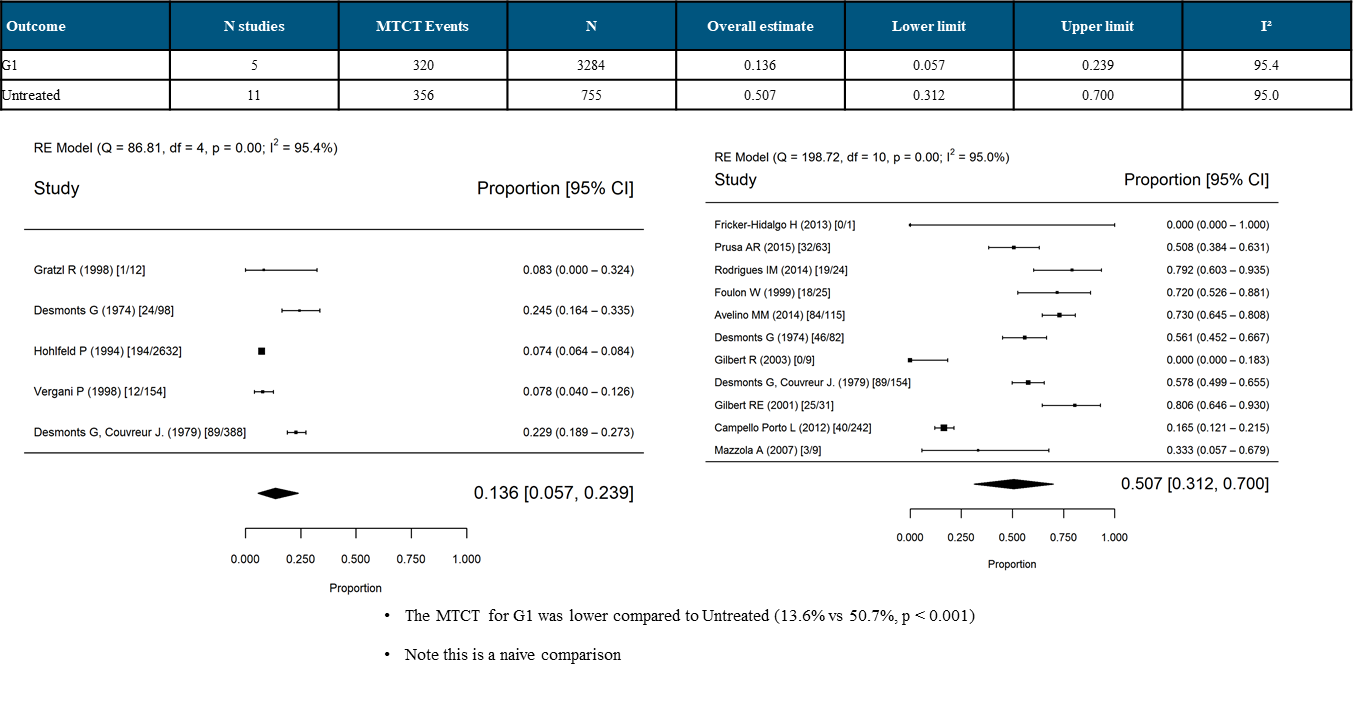


1. MTCT, Spiramycin and/or PSF (G1+G2) vs Untreated, Before 1999


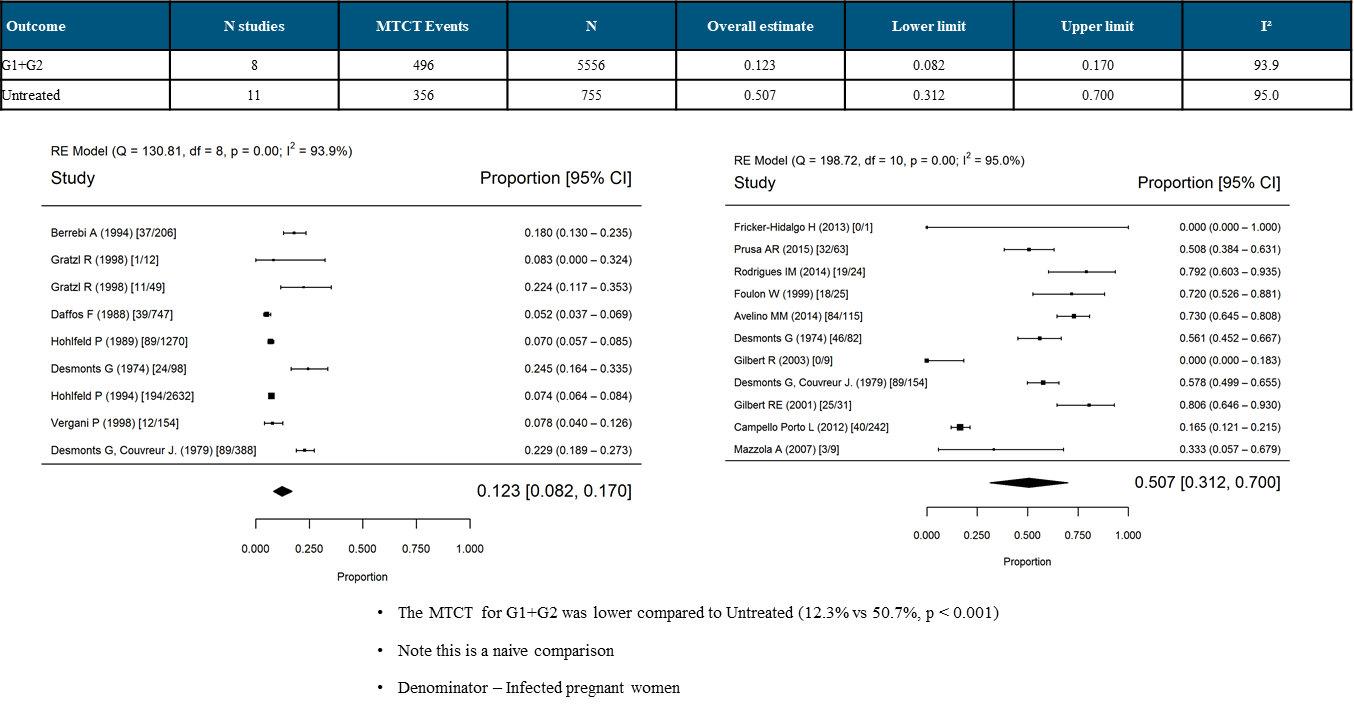


1. MTCT, G1 vs Untreated, 1999-2006


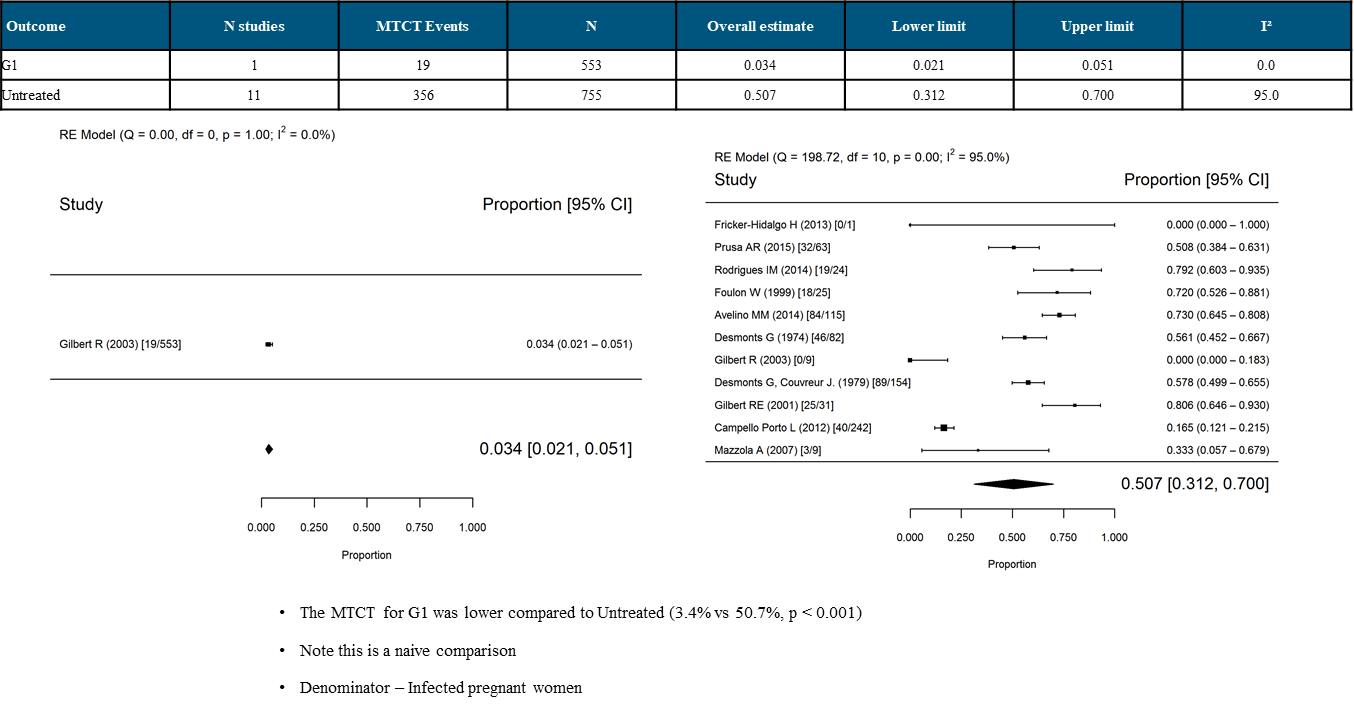


1. MTCT, G1+G2 vs Untreated, 1999-2006


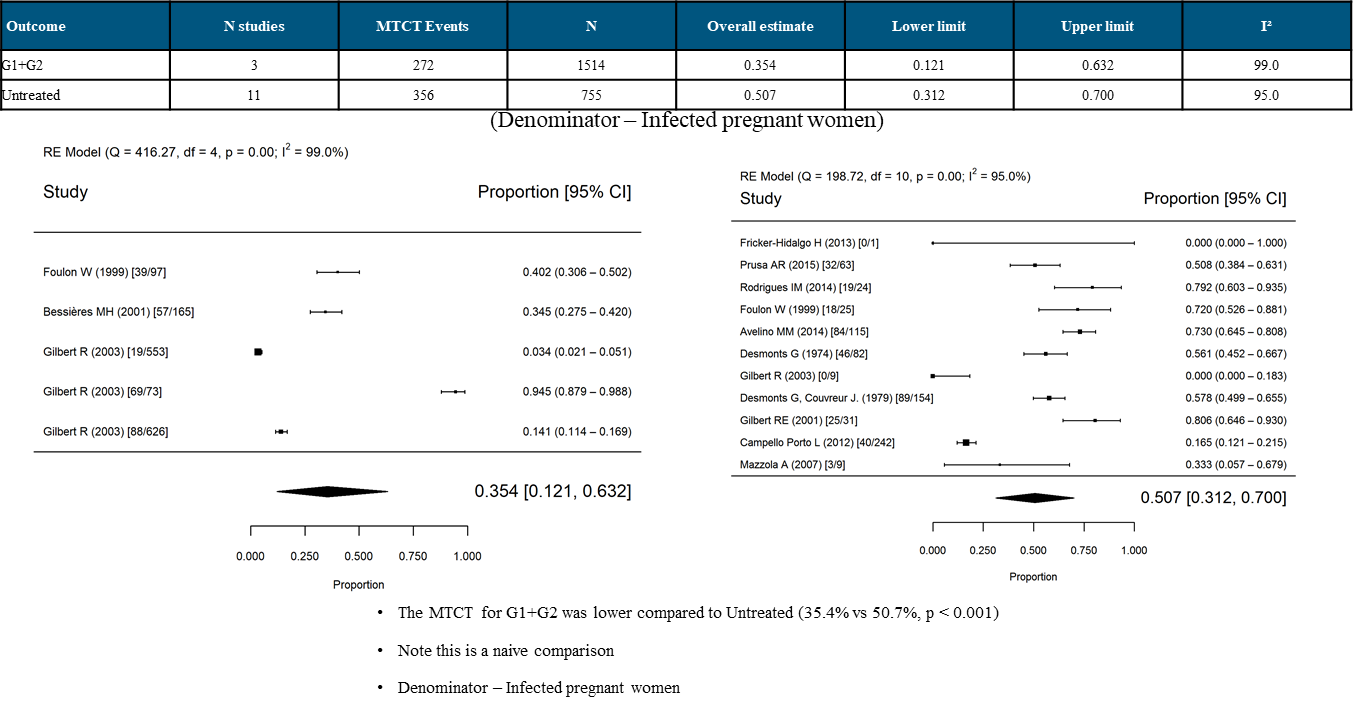


1. MTCT, G1 vs Untreated, After 2006


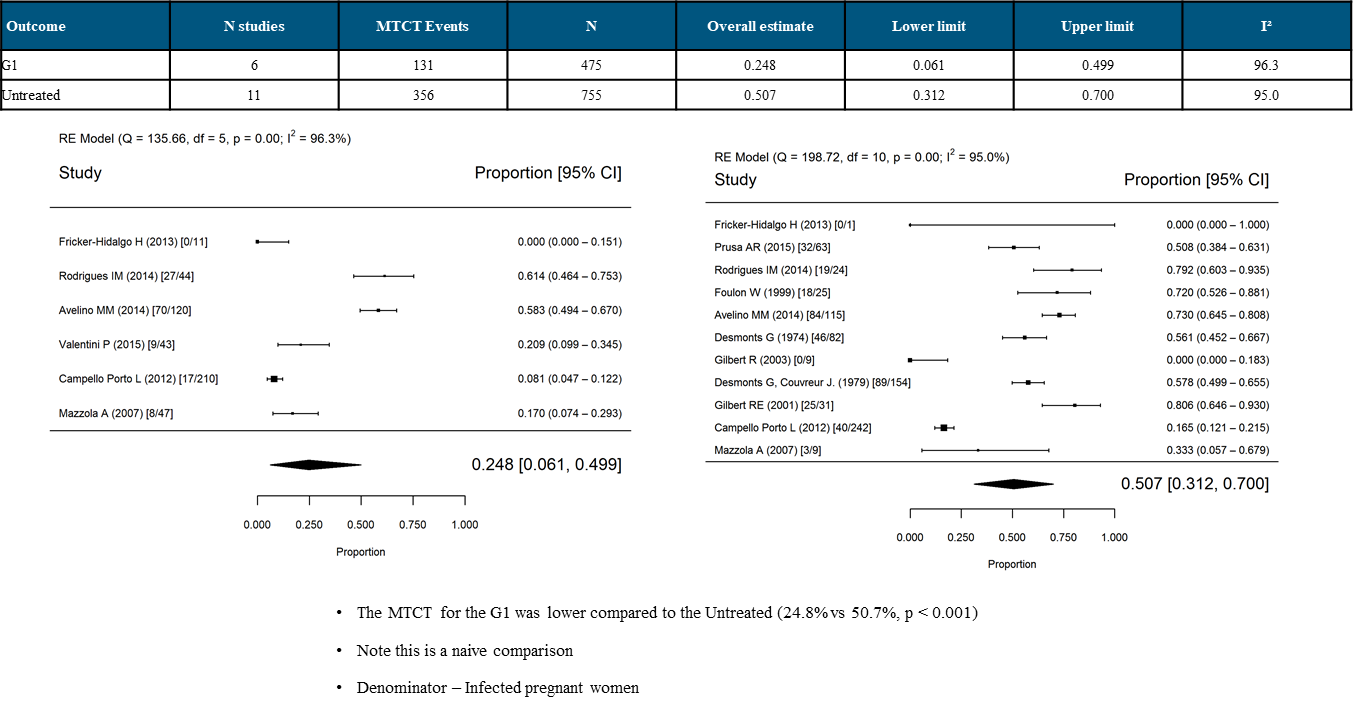


1. MTCT, G1+G2 vs Untreated, After 2006


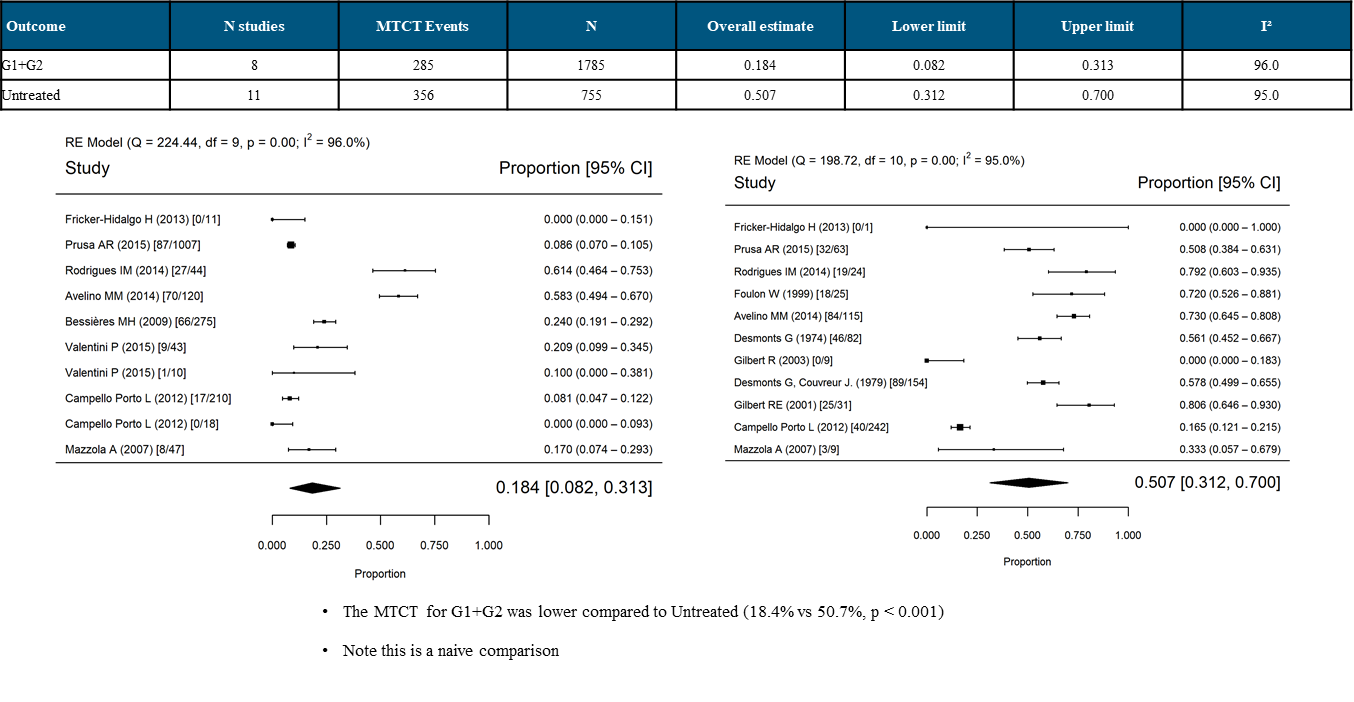


1. MTCT, G1 vs Untreated, 1^st^ Trimester


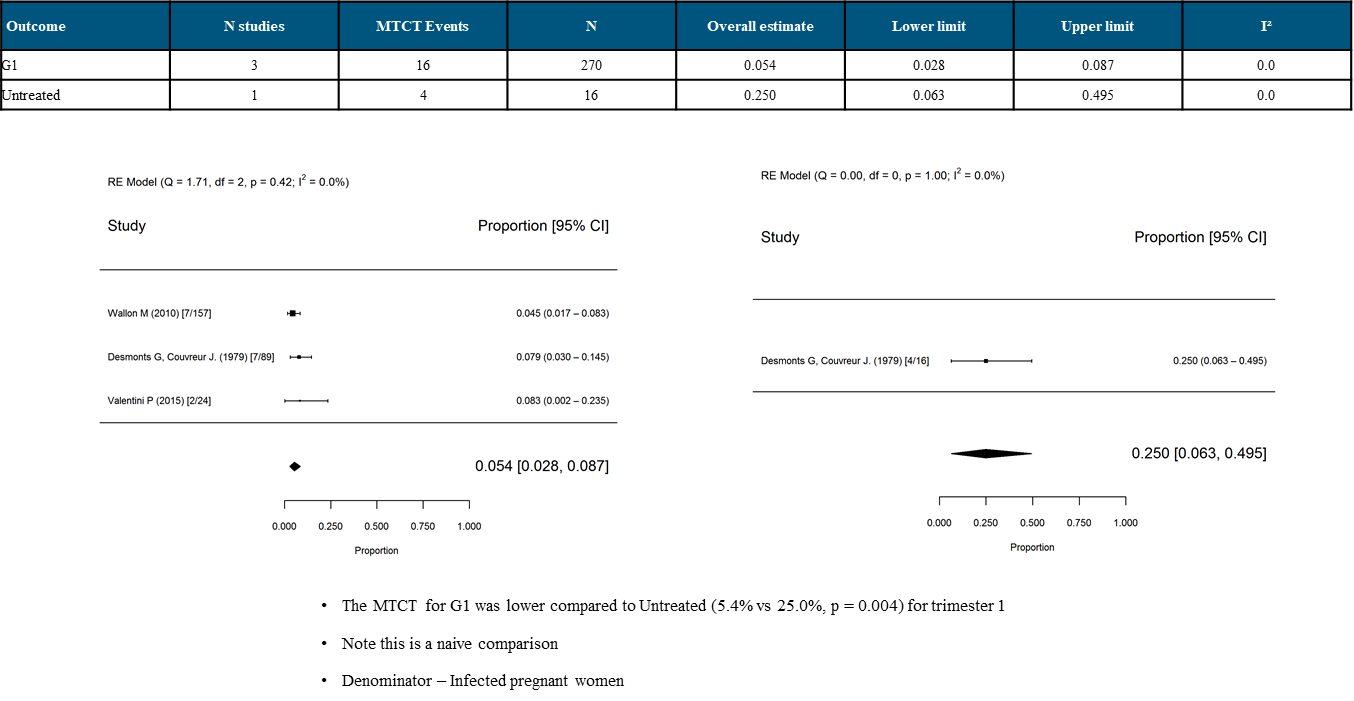


1. MTCT, G1+G2 vs Untreated, 1^st^ Trimester


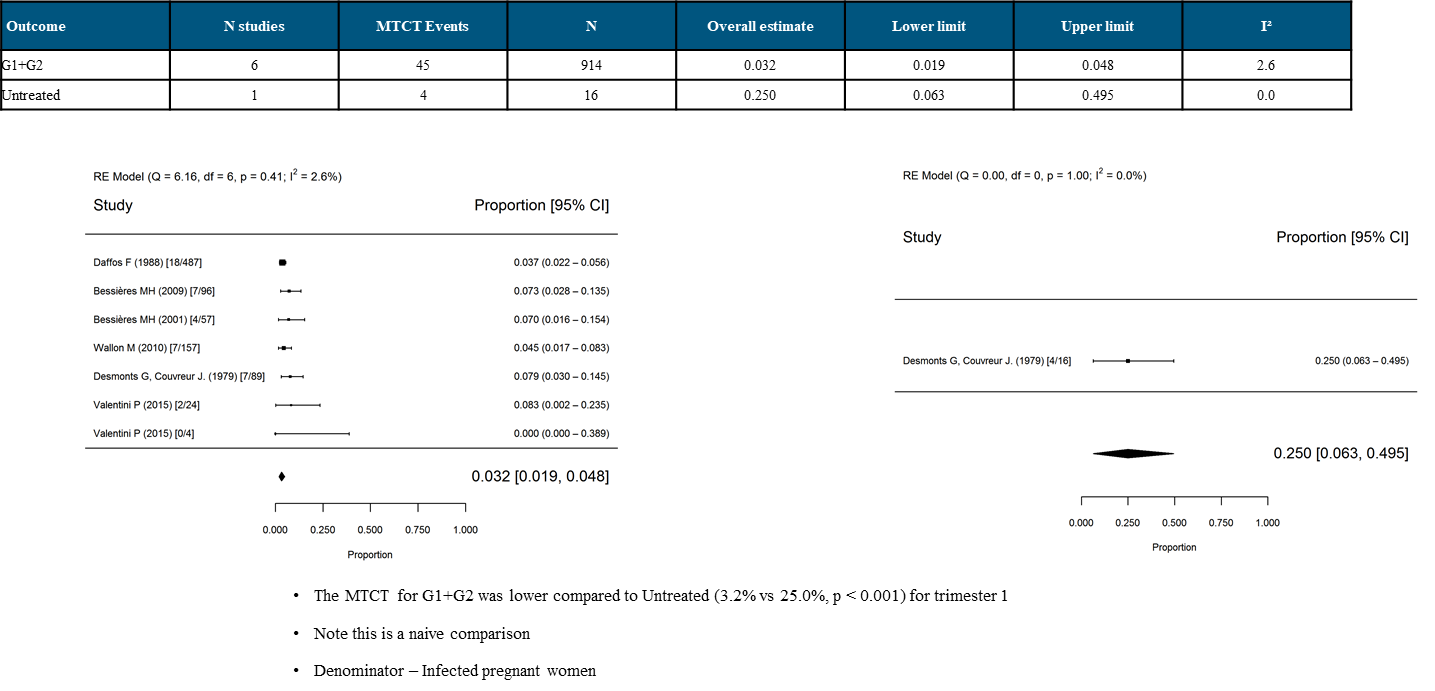


1. MTCT, G1 vs Untreated, 2^nd^ Trimester


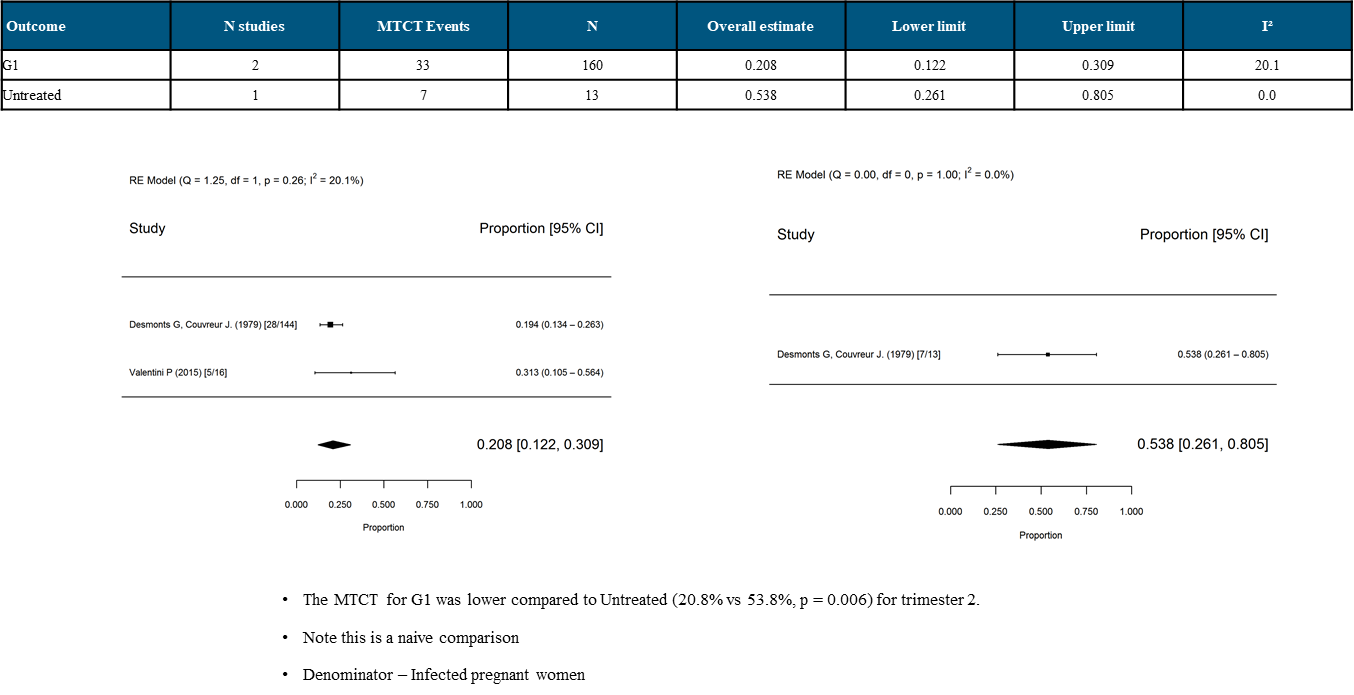


1. MTCT, G1+G2 vs Untreated, 2^nd^ Trimester


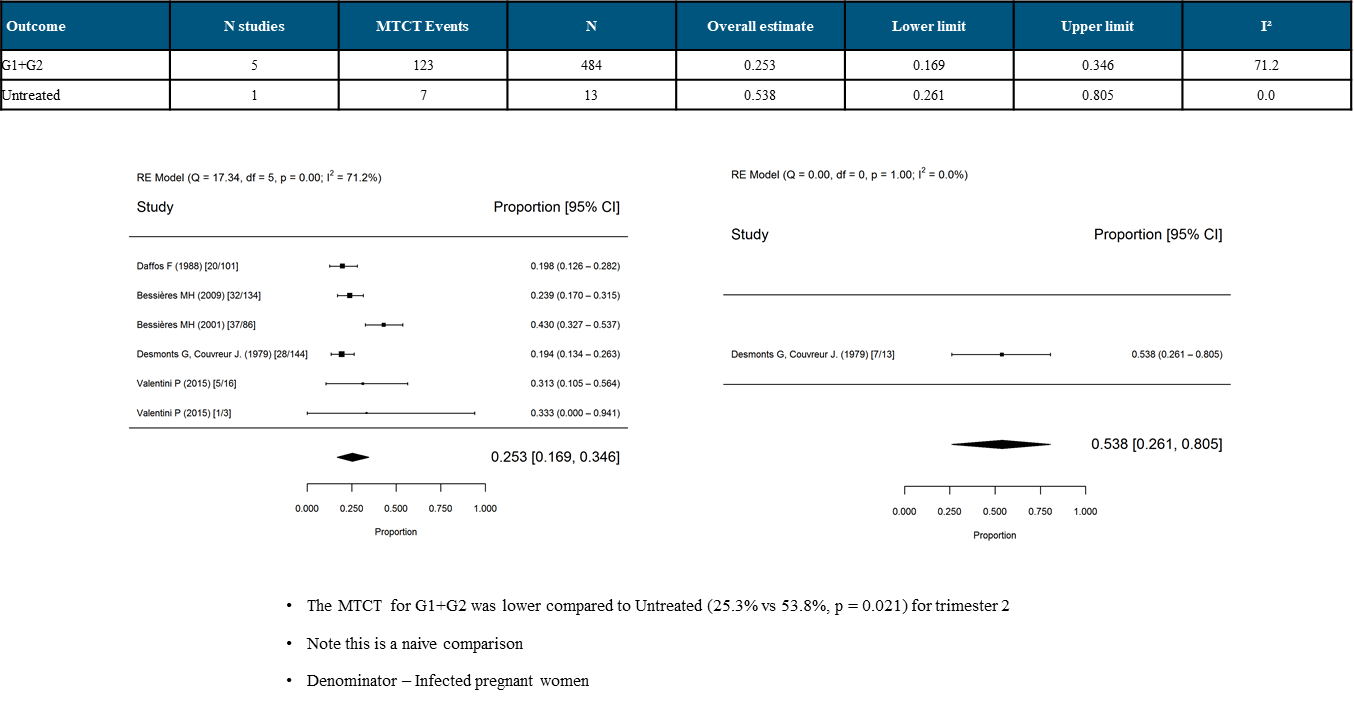


1. MTCT, G1 vs Untreated, 3^rd^ Trimester


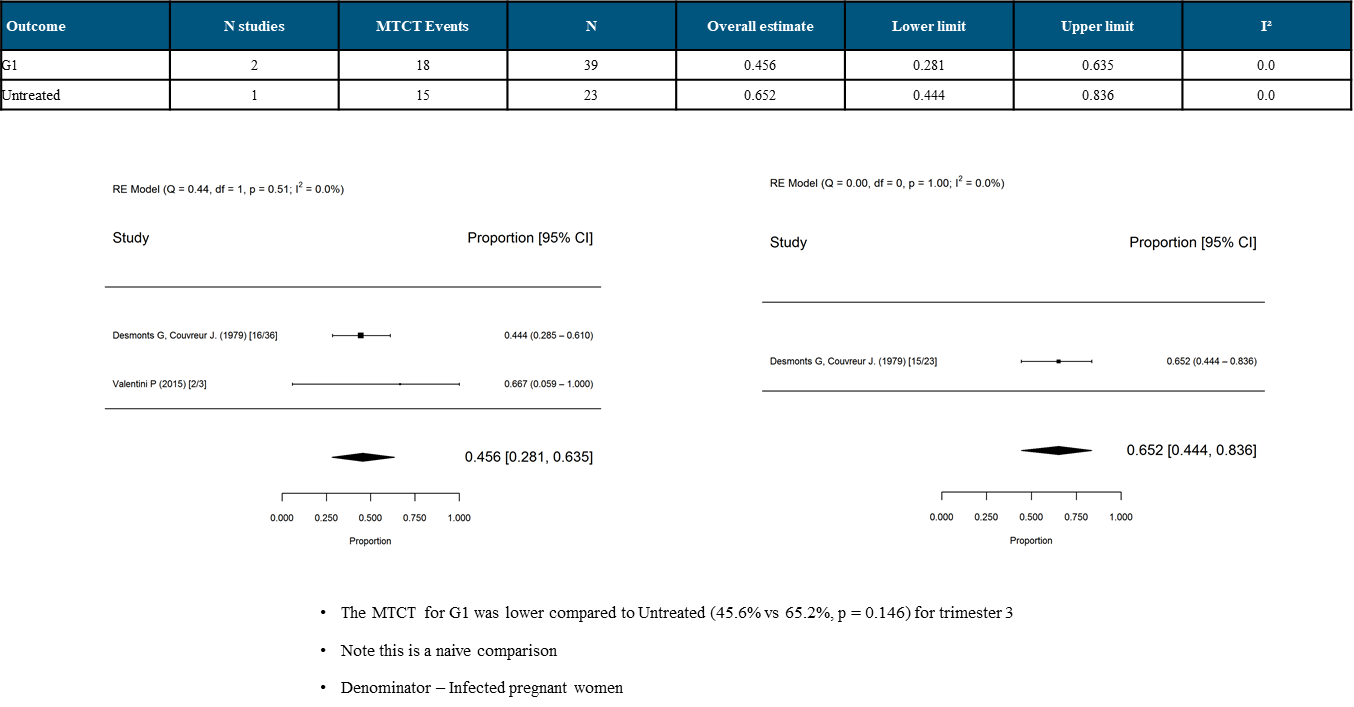


1. MTCT, G1+G2 vs Untreated, 3^rd^ Trimester


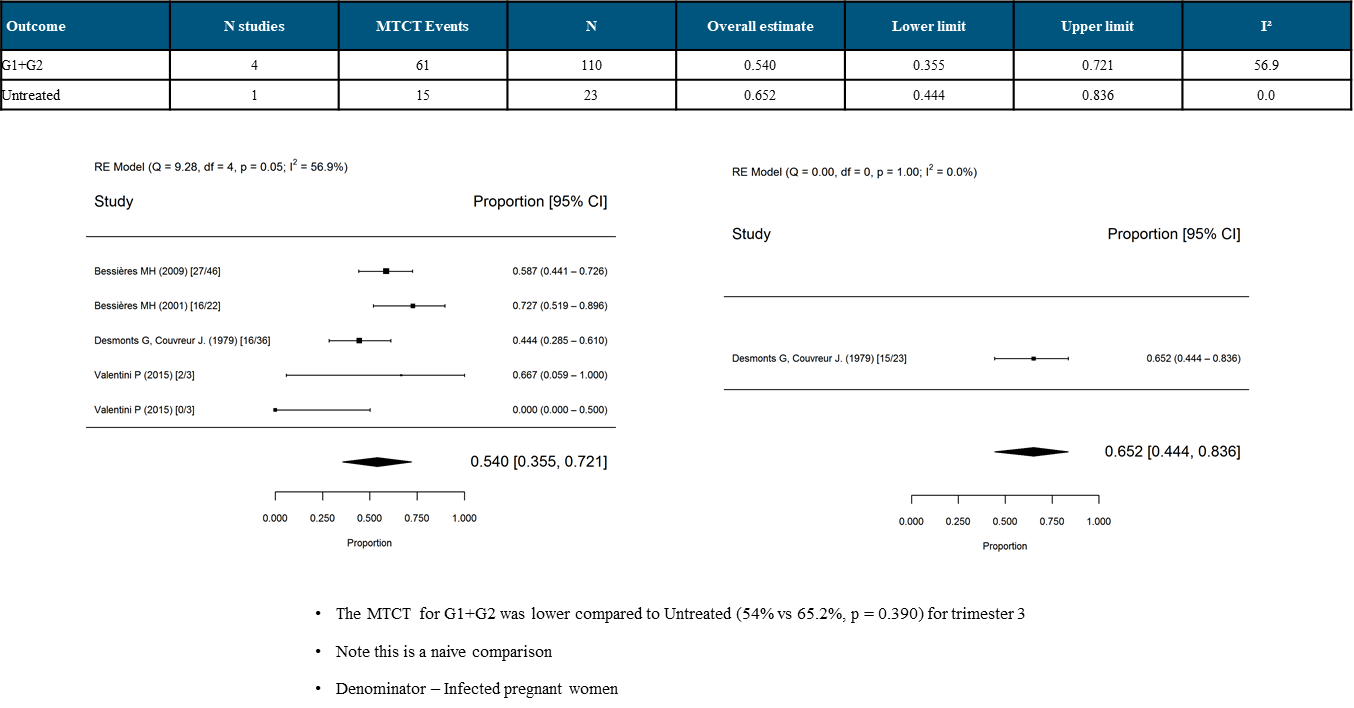


1. MTCT, G1 vs Untreated, 1^st^ and 2^nd^ Trimesters


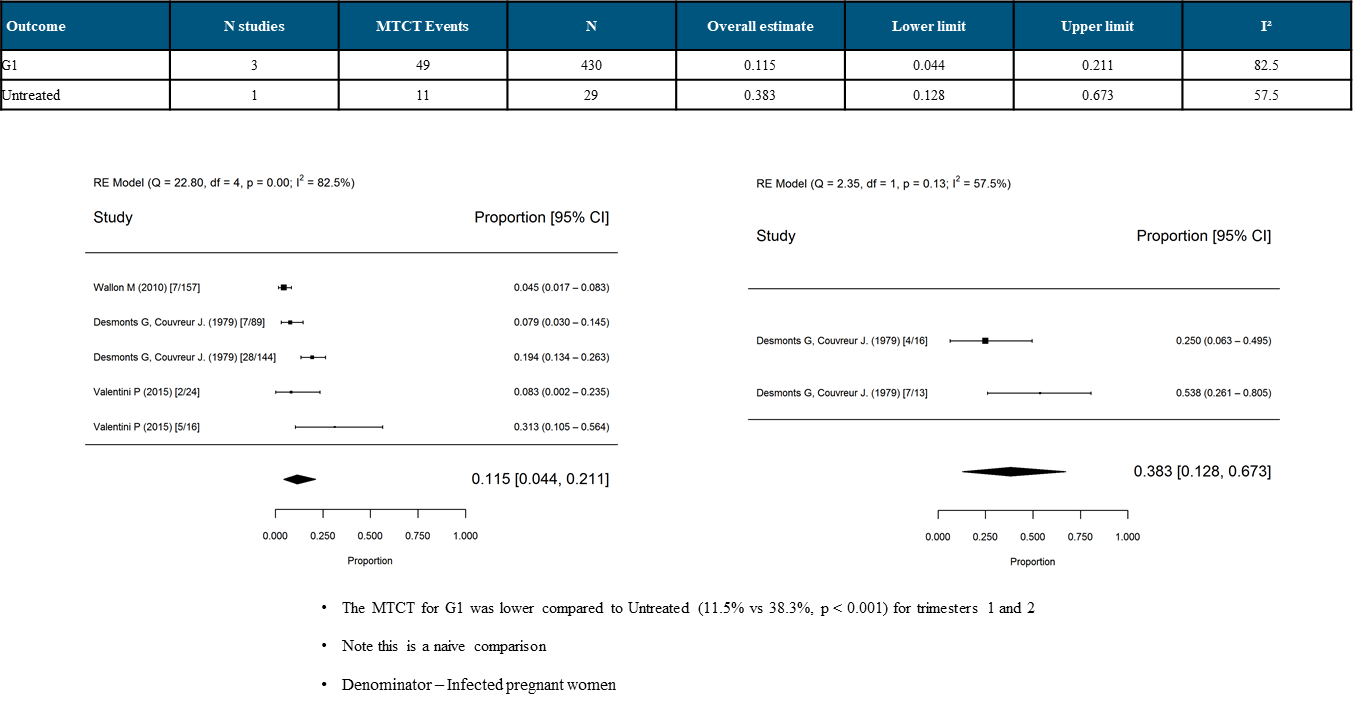


1. MTCT, G1+G2 vs Untreated, 1^st^ and 2^nd^ Trimesters


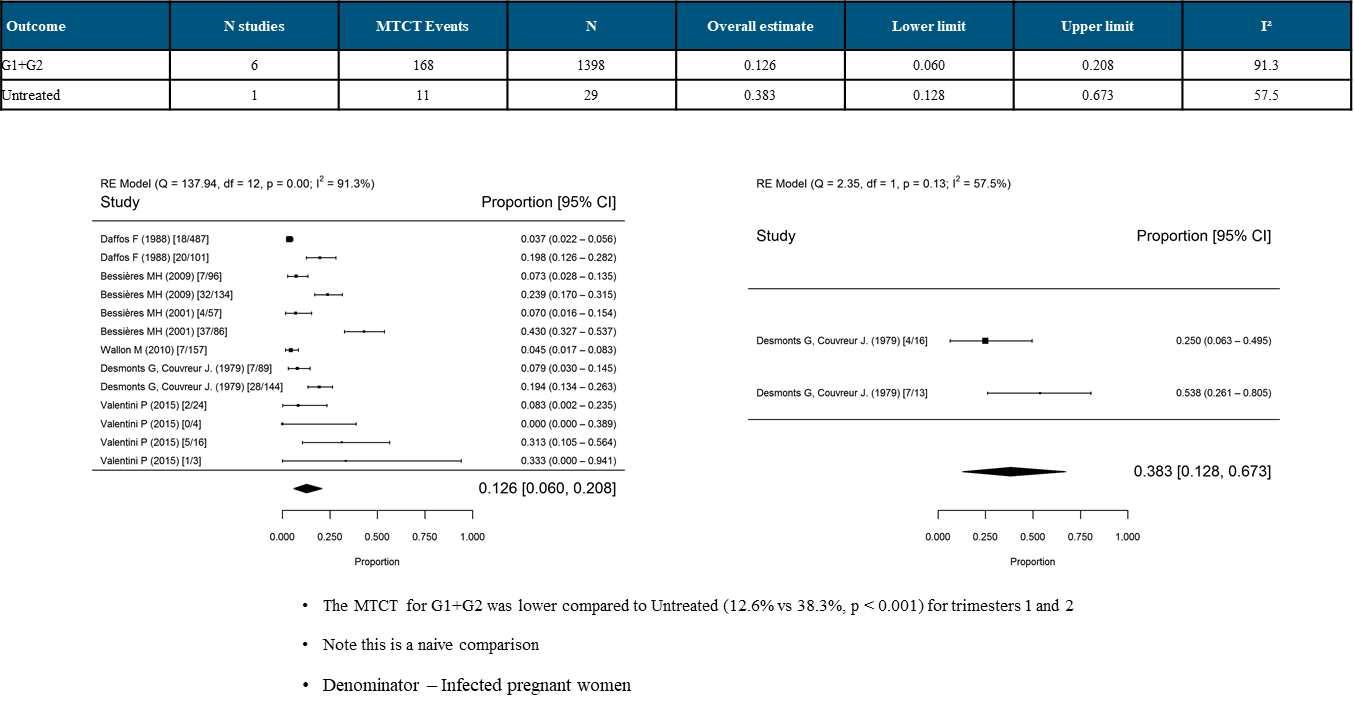


### Mortality due to CT Excluding *Elective* Terminations due to Fetal Infection - Subgroup Analyses

1. Mortality Excluding Elective Terminations, G1 vs Untreated, Before 1999 – Up to 1 year


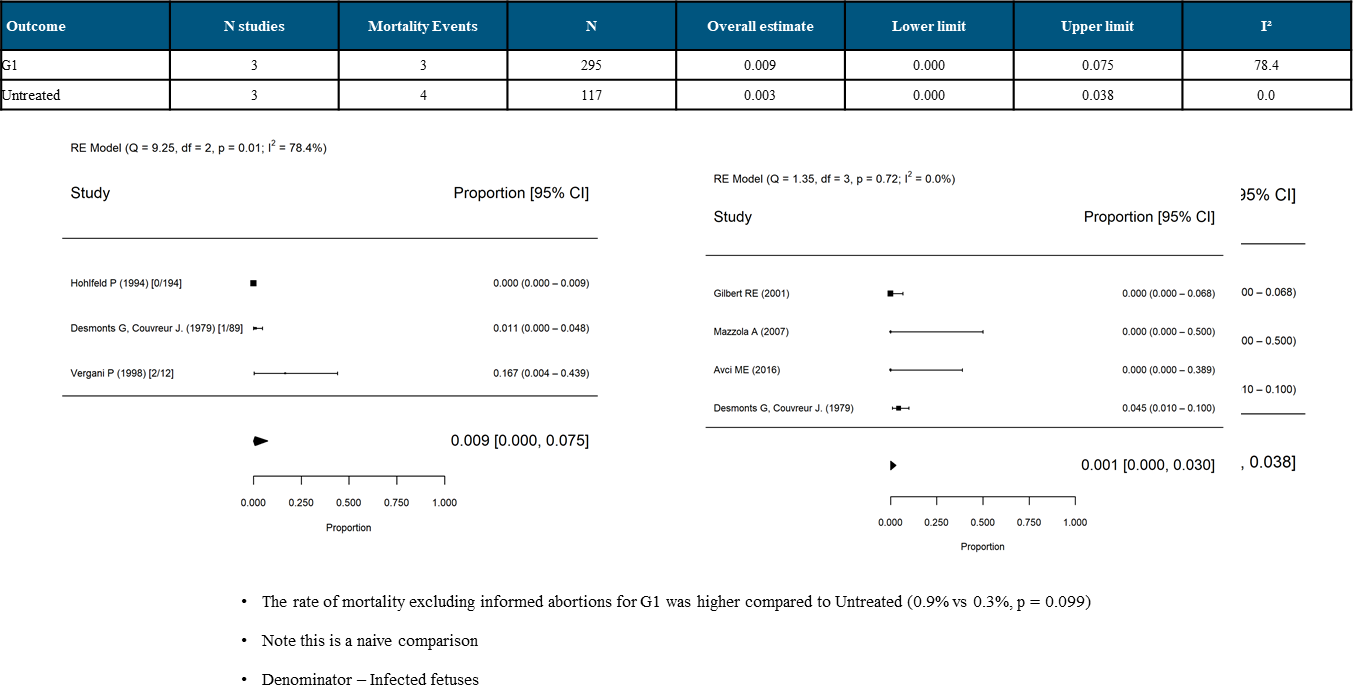


1. Mortality Excluding Elective Terminations, G1+G2 vs Untreated, Before 1999 – Up to 1 year


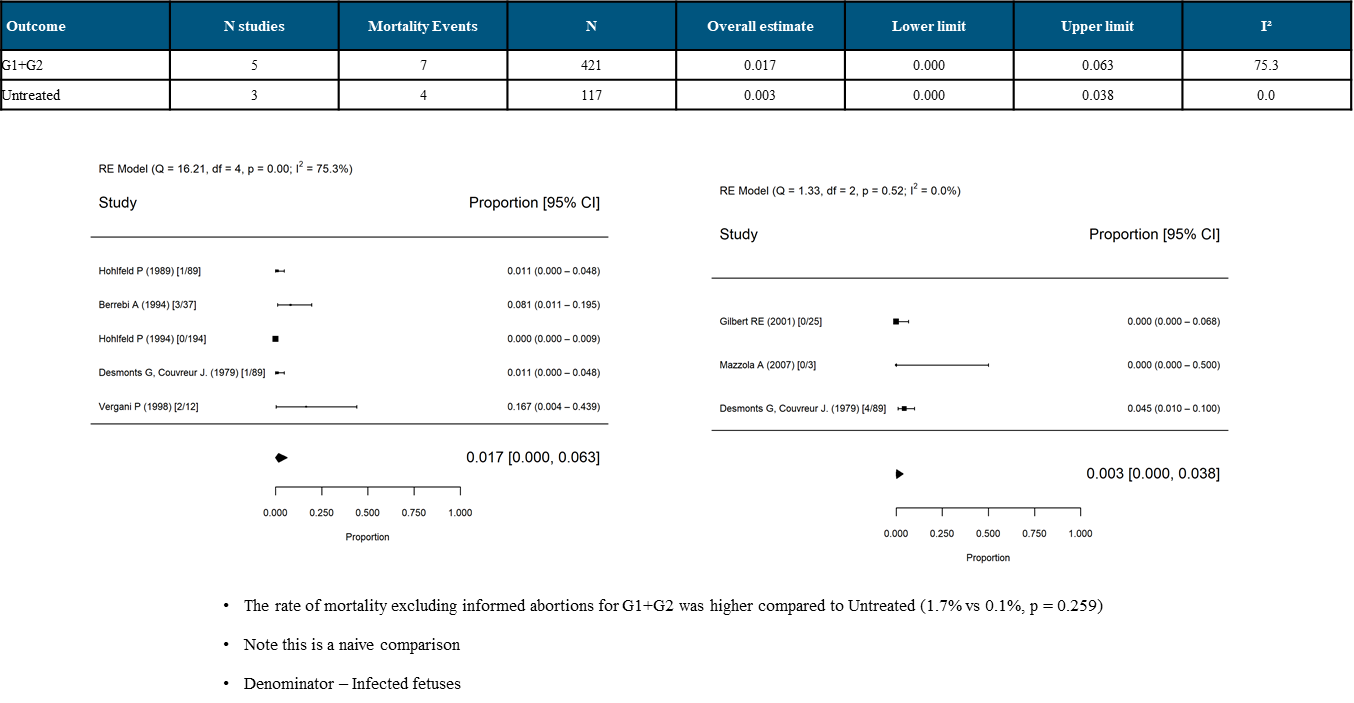


1. Mortality Excluding Elective Terminations, G1 vs Untreated, After 2006 – Up to 1 year


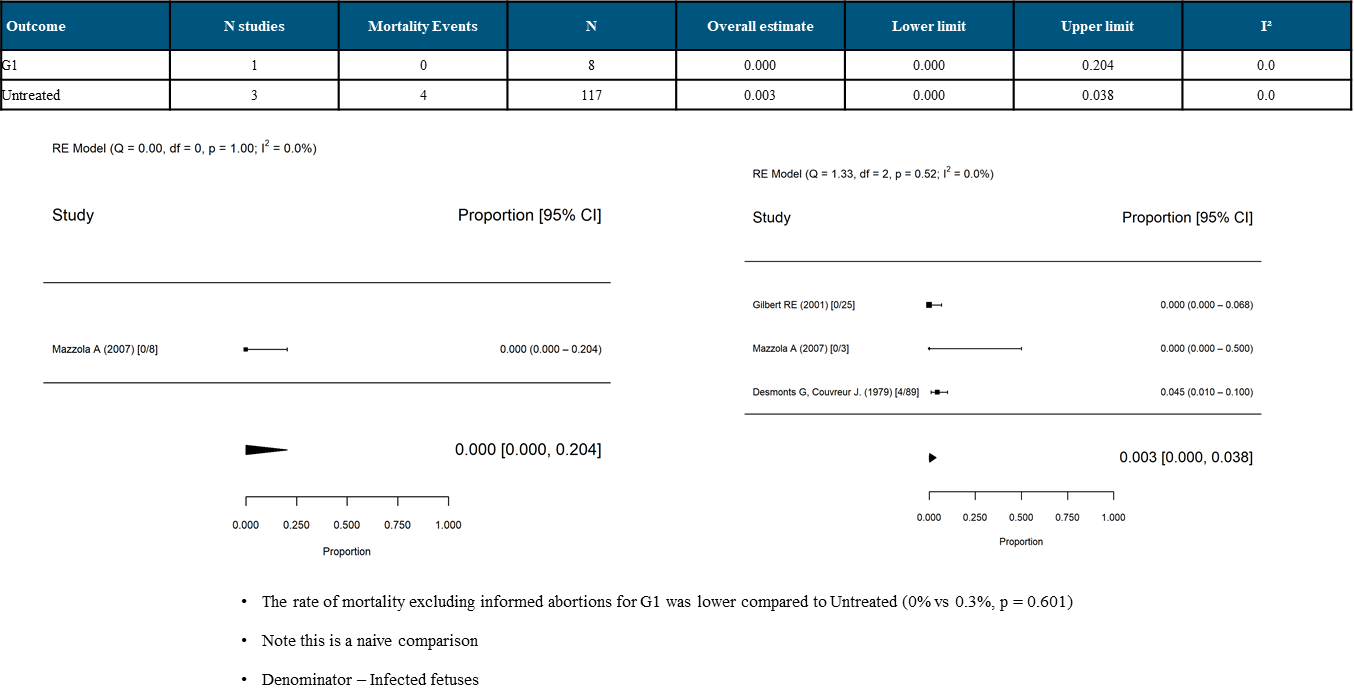


1. Mortality Excluding Elective Terminations, G1+G2 vs Untreated, After 2006 – Up to 1 year


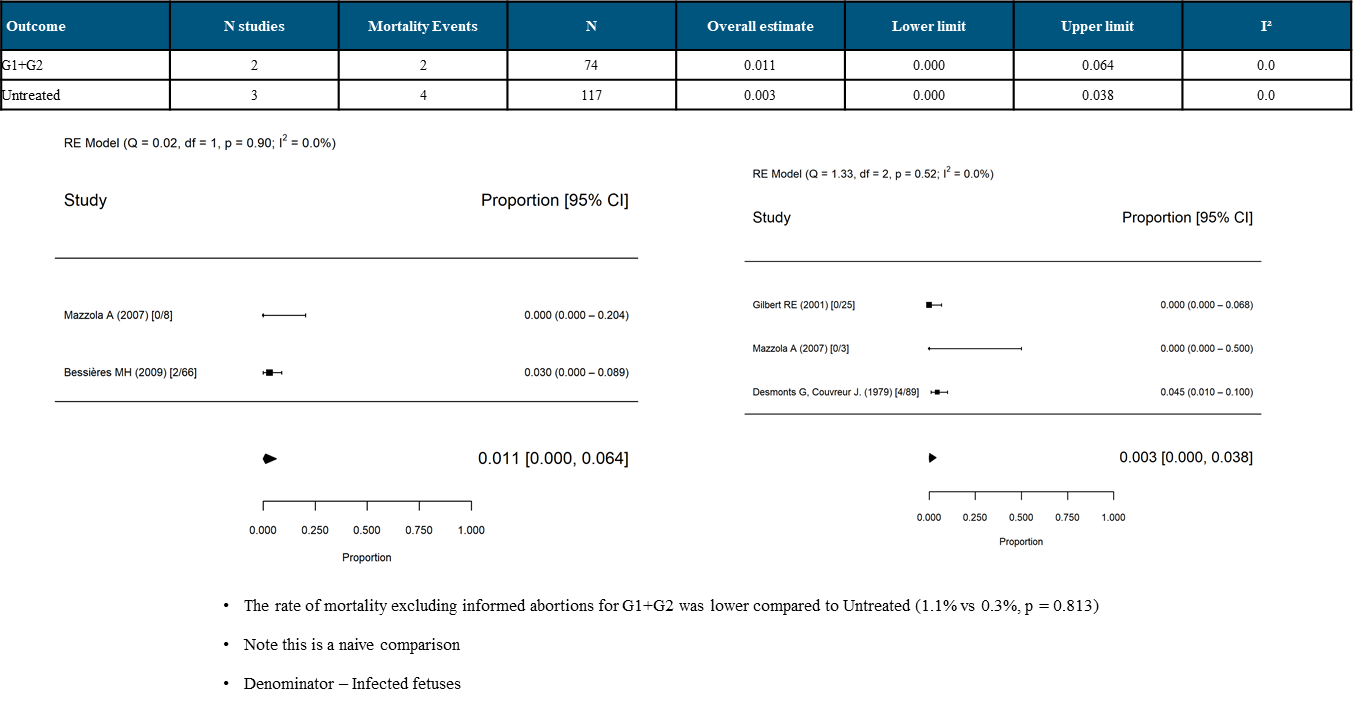


1. Mortality Excluding Elective Terminations, G1+G2, 1974-2016 – Beyond 1 year


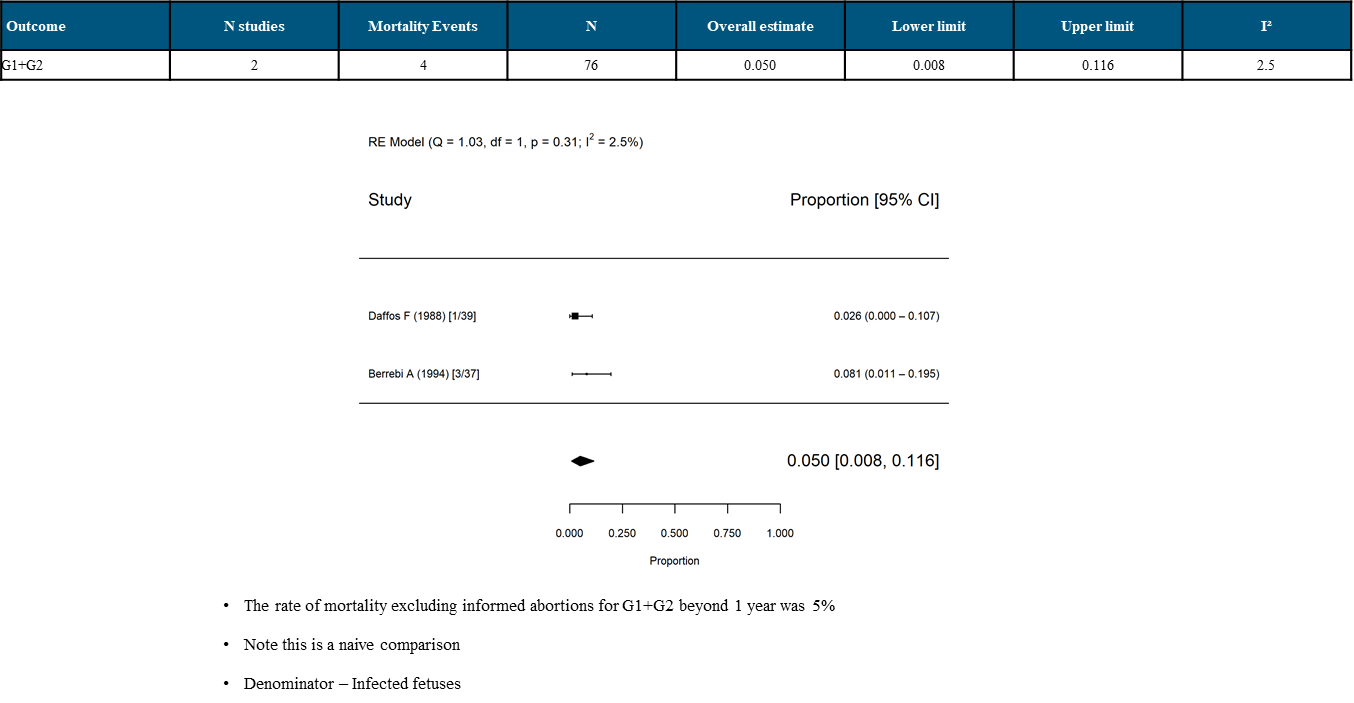


1. Mortality Excluding Elective Terminations, G1+G2, Before 1999 – Beyond 1 year


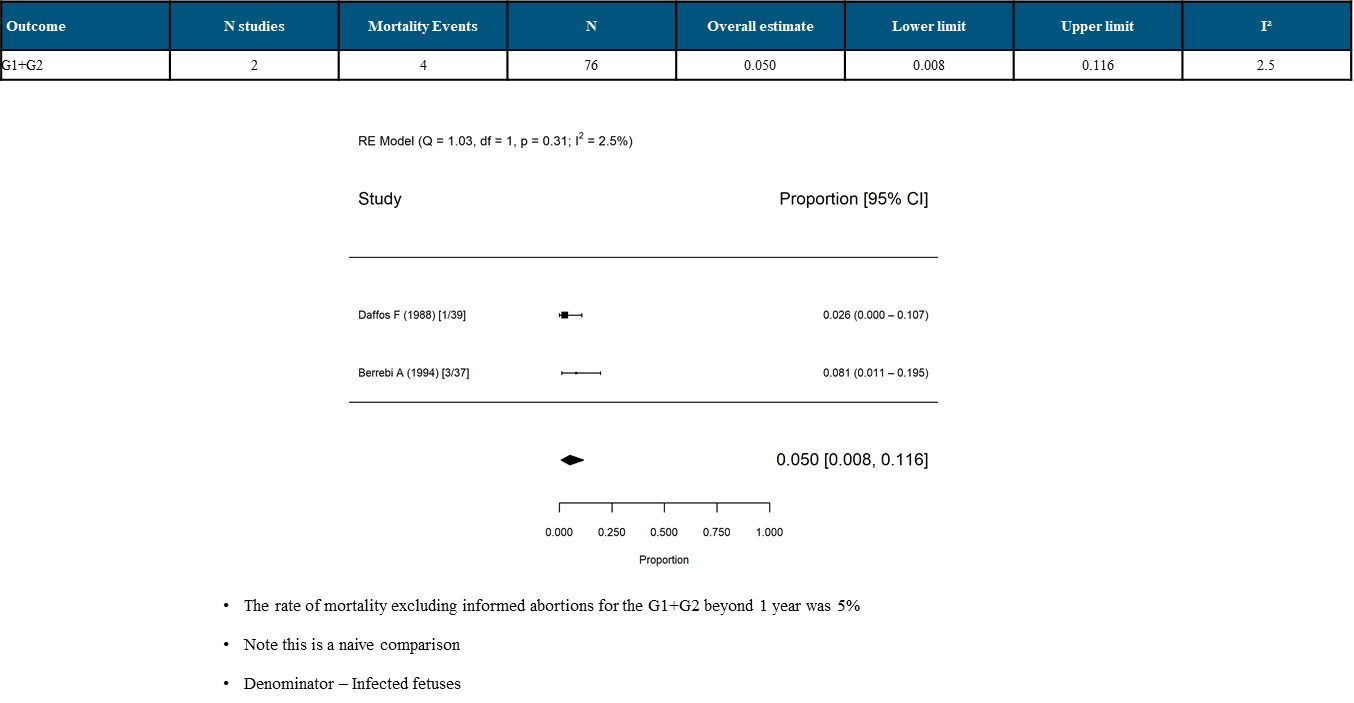


### Serious/Severe Sequelae and All Mortality: Infected Pregnant Women - Subgroup Analyses

1. All Serious/Severe Sequelae and All Mortality, G1 vs Untreated, Before 1999 – Up to 1 year


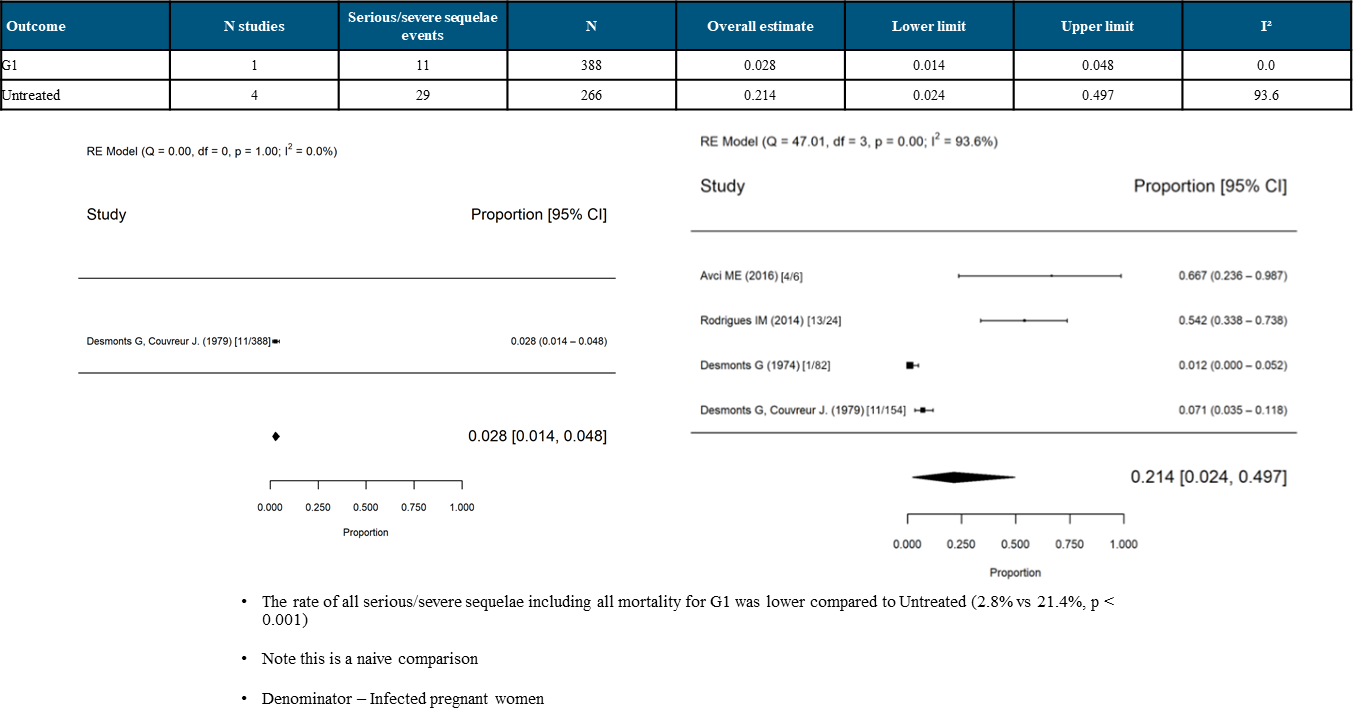


1. All Serious/Severe Sequelae and All Mortality, G1+G2 vs Untreated, Before 1999 – Up to 1 year


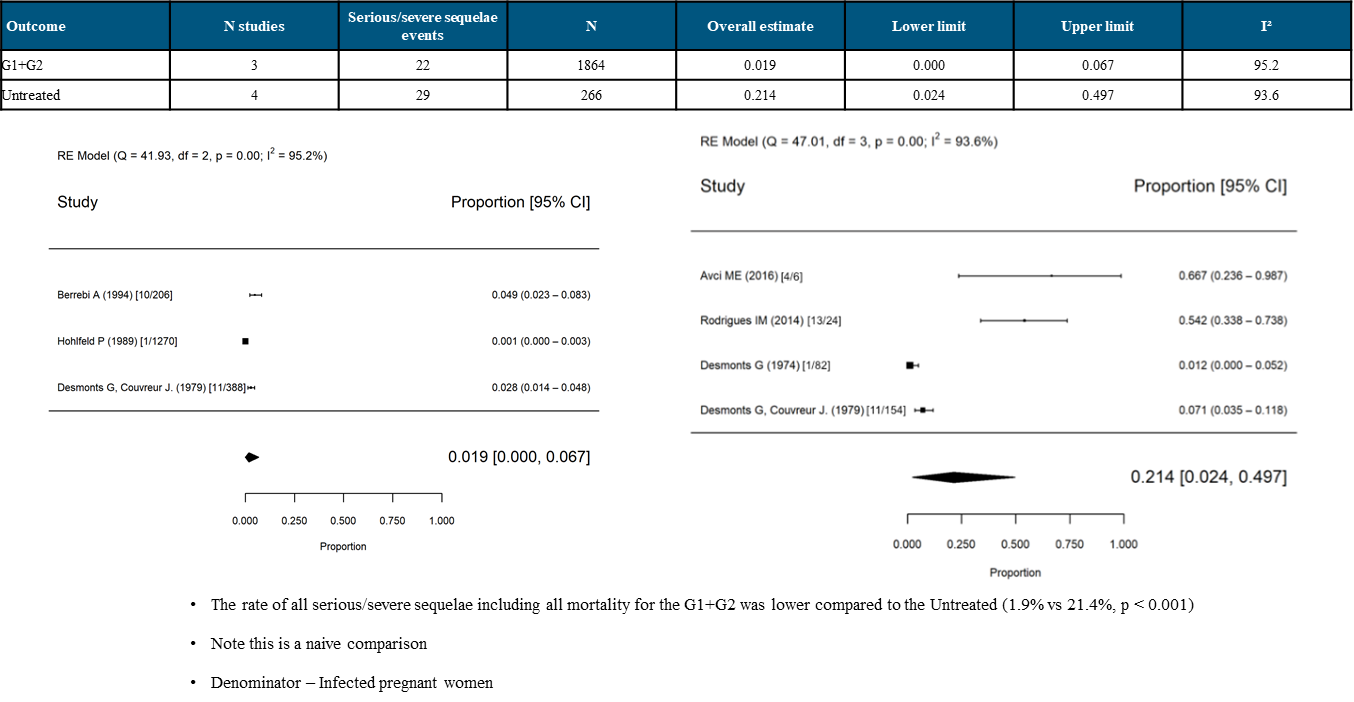


1. All Serious/Severe Sequelae and All Mortality, G1 vs Untreated, After 2006 – Up to 1 year


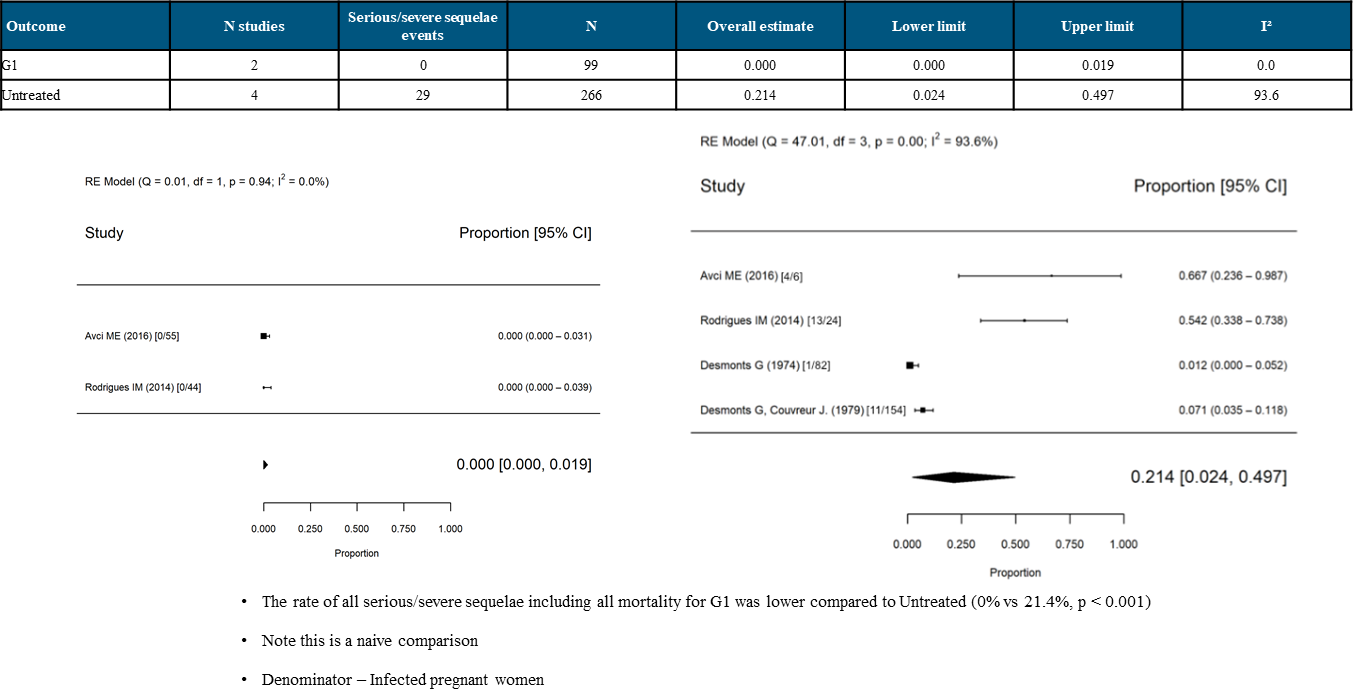


1. All Serious/Severe Sequelae and All Mortality, G1+G2 vs Untreated, After 2006 – Up to 1 year


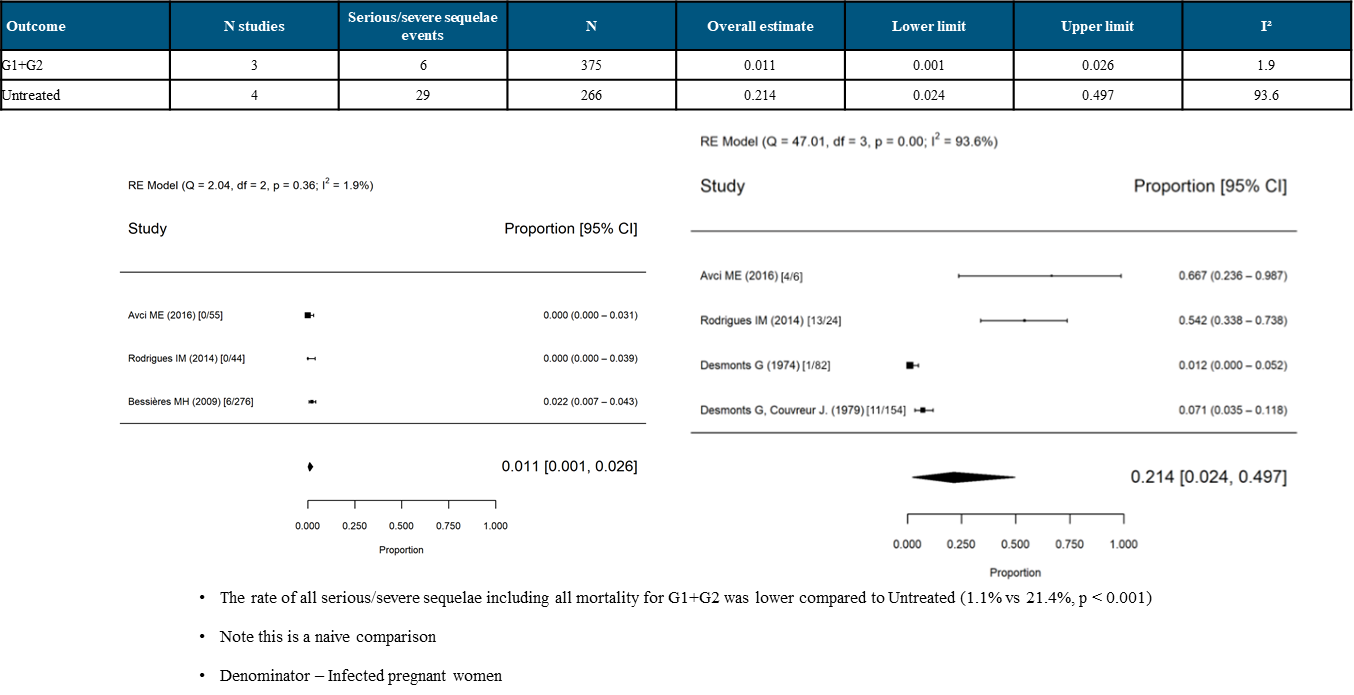


1. All Serious/Severe Sequelae and All Mortality, G1, 1974-2016 – Beyond 1 year


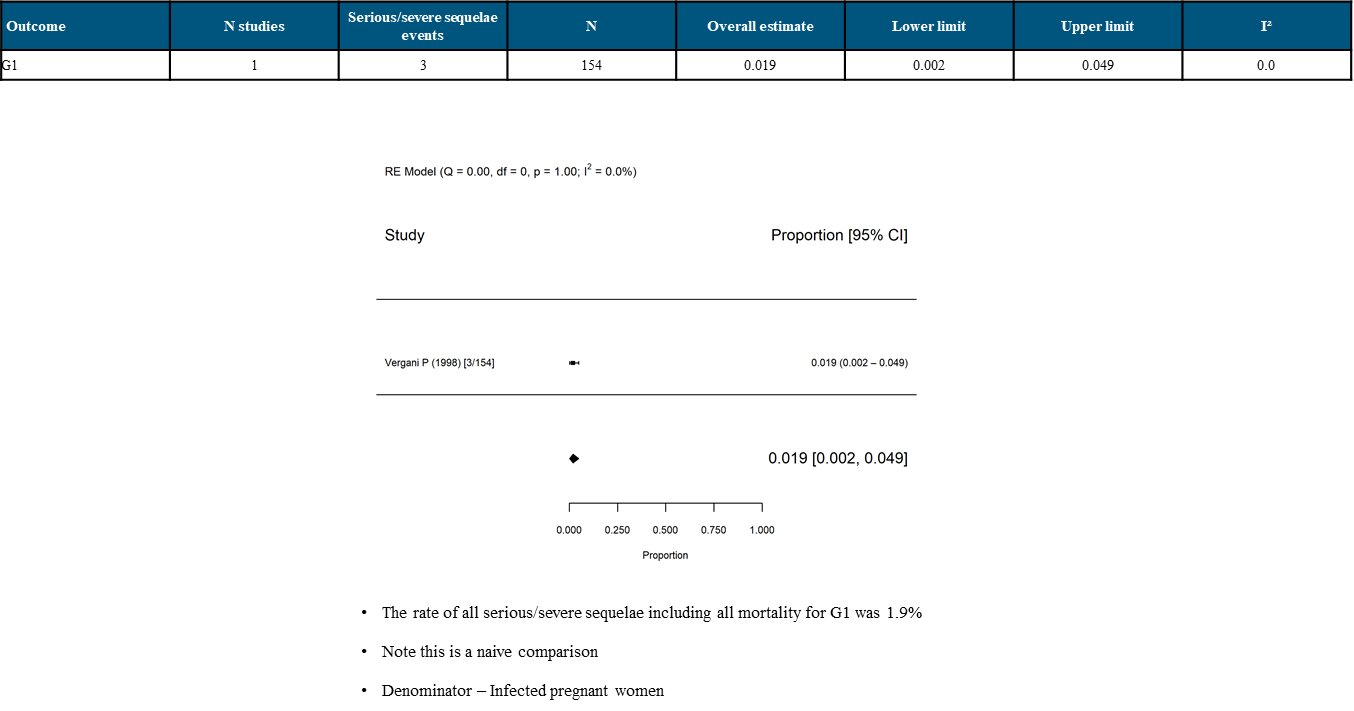


1. All Serious/Severe Sequelae and All Mortality, G1+G2, 1974-2016 – Beyond 1 year


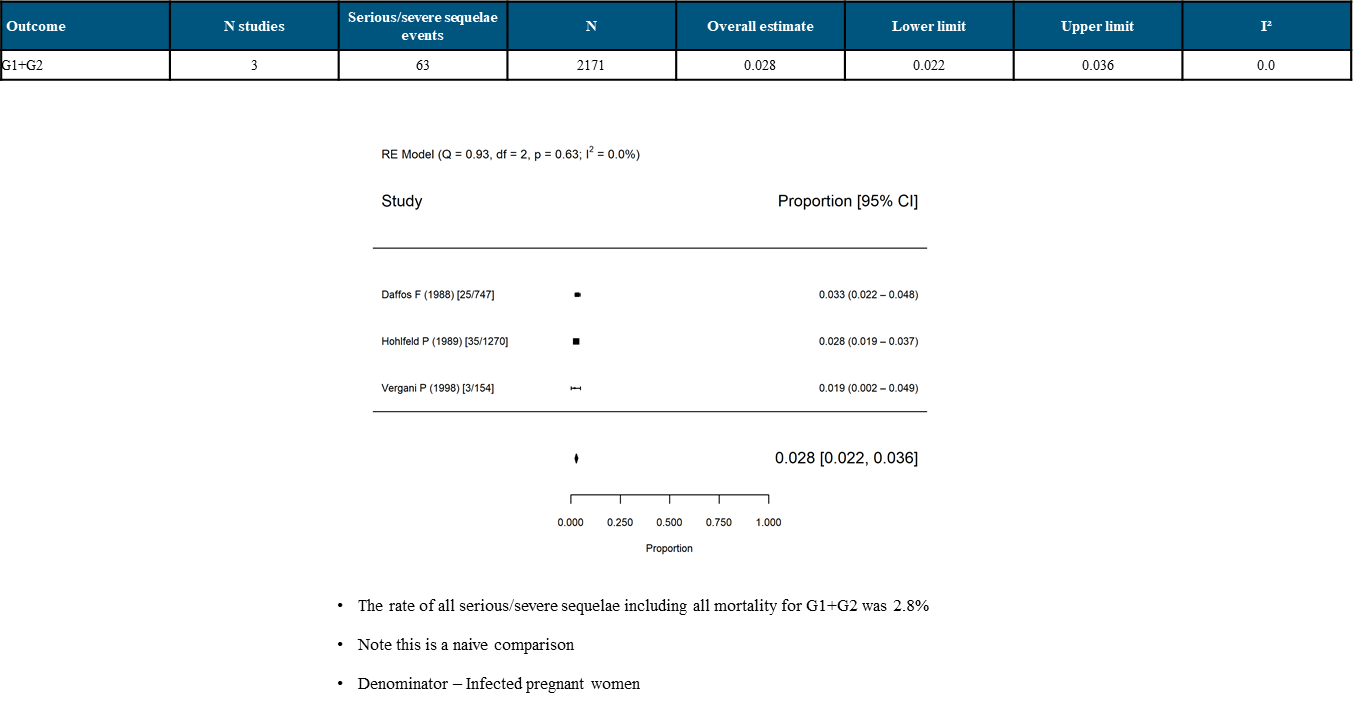


1. All Serious/Severe Sequelae and All Mortality, G1, Before 1999 – Beyond 1 year


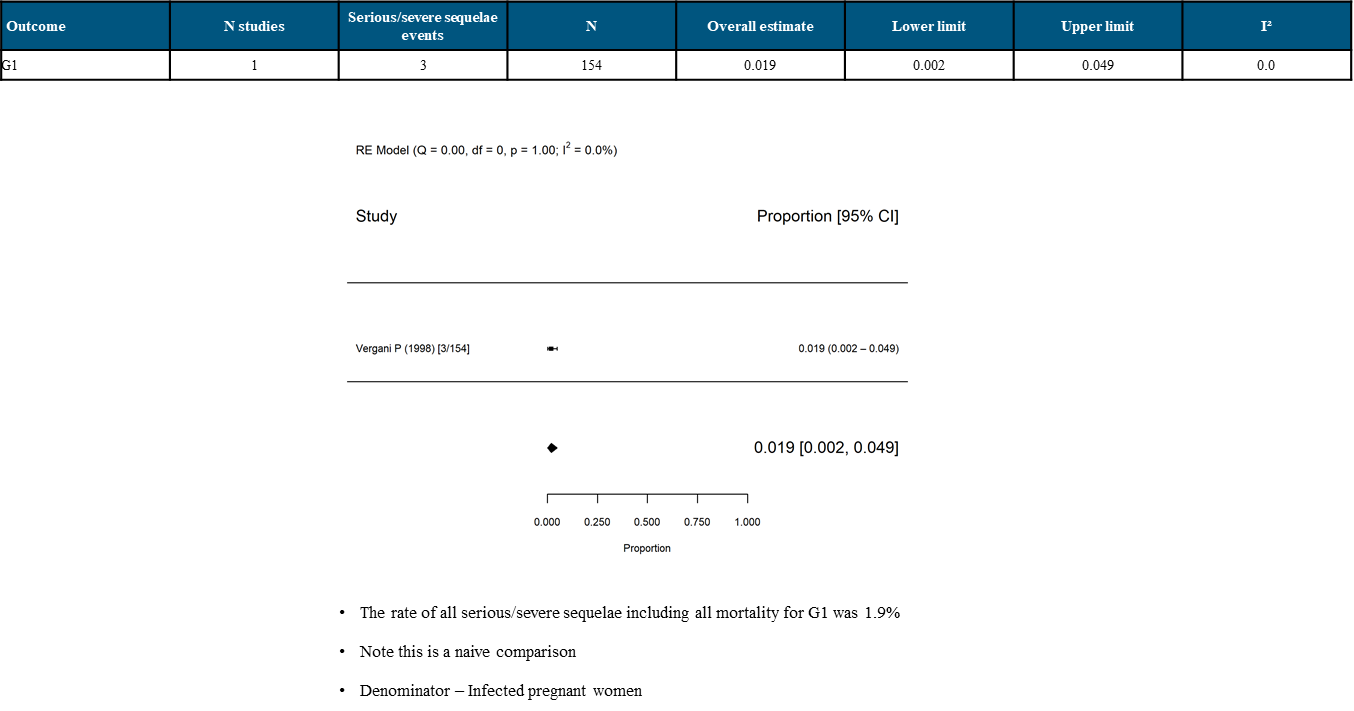


1. All Serious/Severe Sequelae and All Mortality, G1+G2, Before 1999 – Beyond 1 year


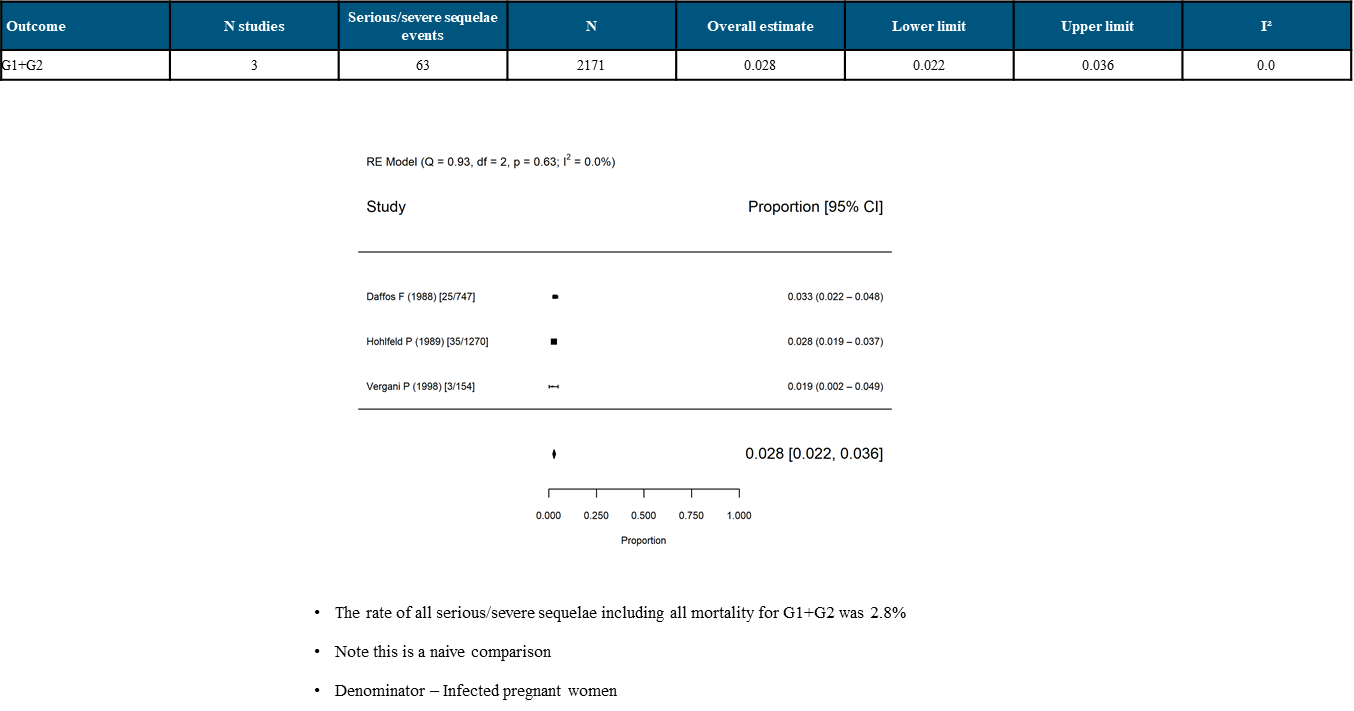


### Serious/Severe Sequelae and All Mortality: Infected Fetuses - Subgroup Analyses

1. All Serious/Severe Sequelae and All Mortality, G1 vs Untreated, Before 1999 – Up to 1 year


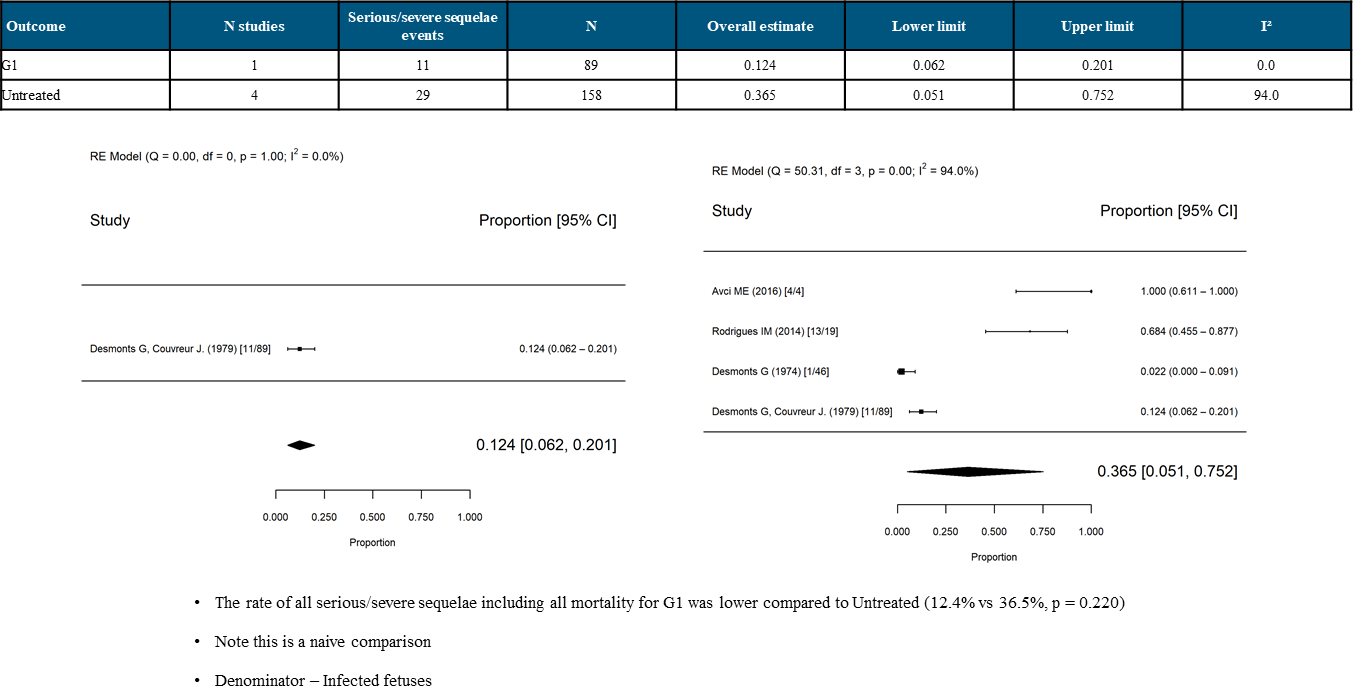


1. All Serious/Severe Sequelae and All Mortality, G1+G2 vs Untreated, Before 1999 – Up to 1 year


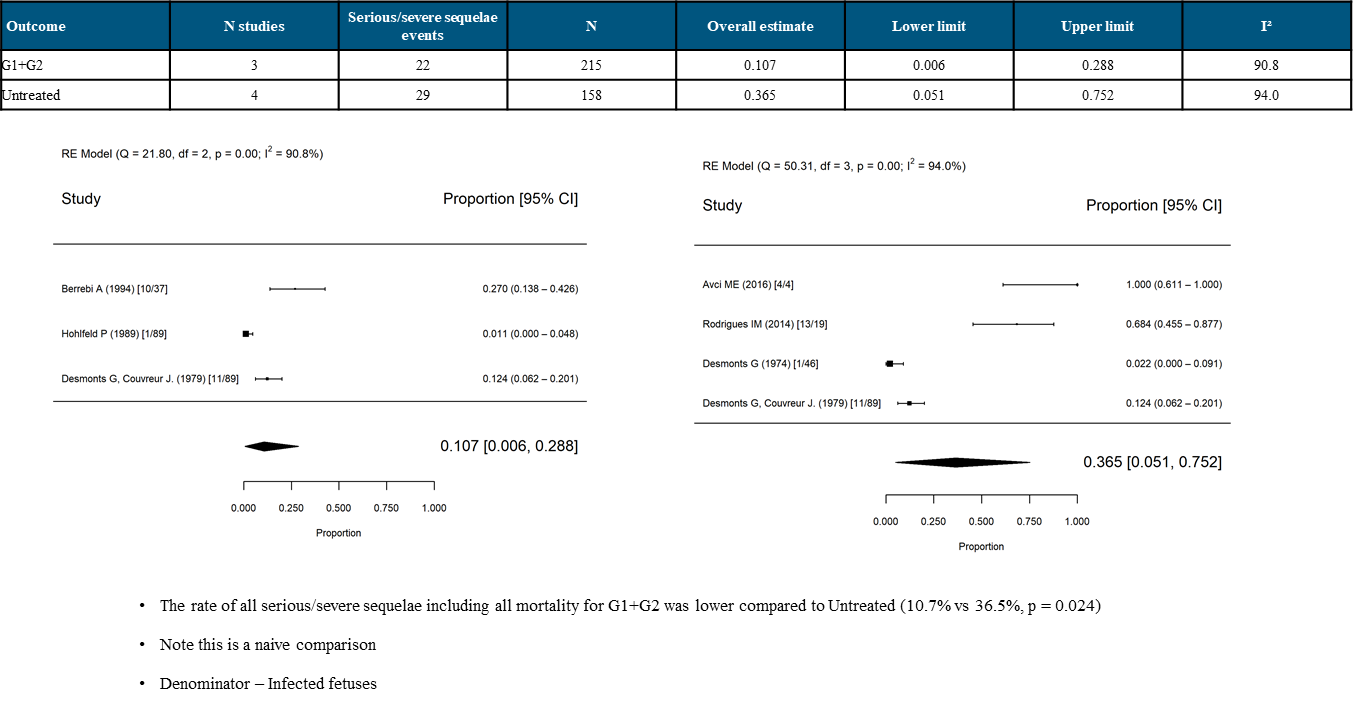


1. All Serious/Severe Sequelae and All Mortality, G1 vs Untreated, After 2006 – Up to 1 year


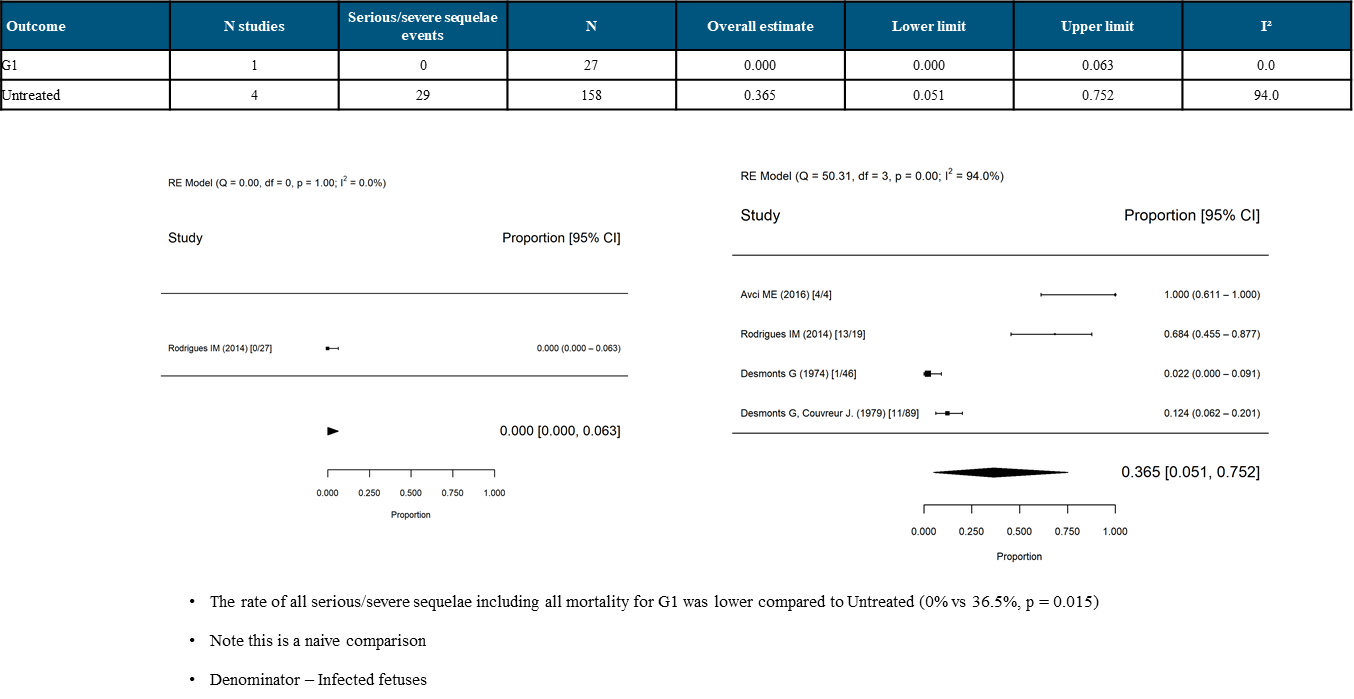


1. All Serious/Severe Sequelae and All Mortality, G1+G2 vs Untreated, After 2006 – Up to 1 year


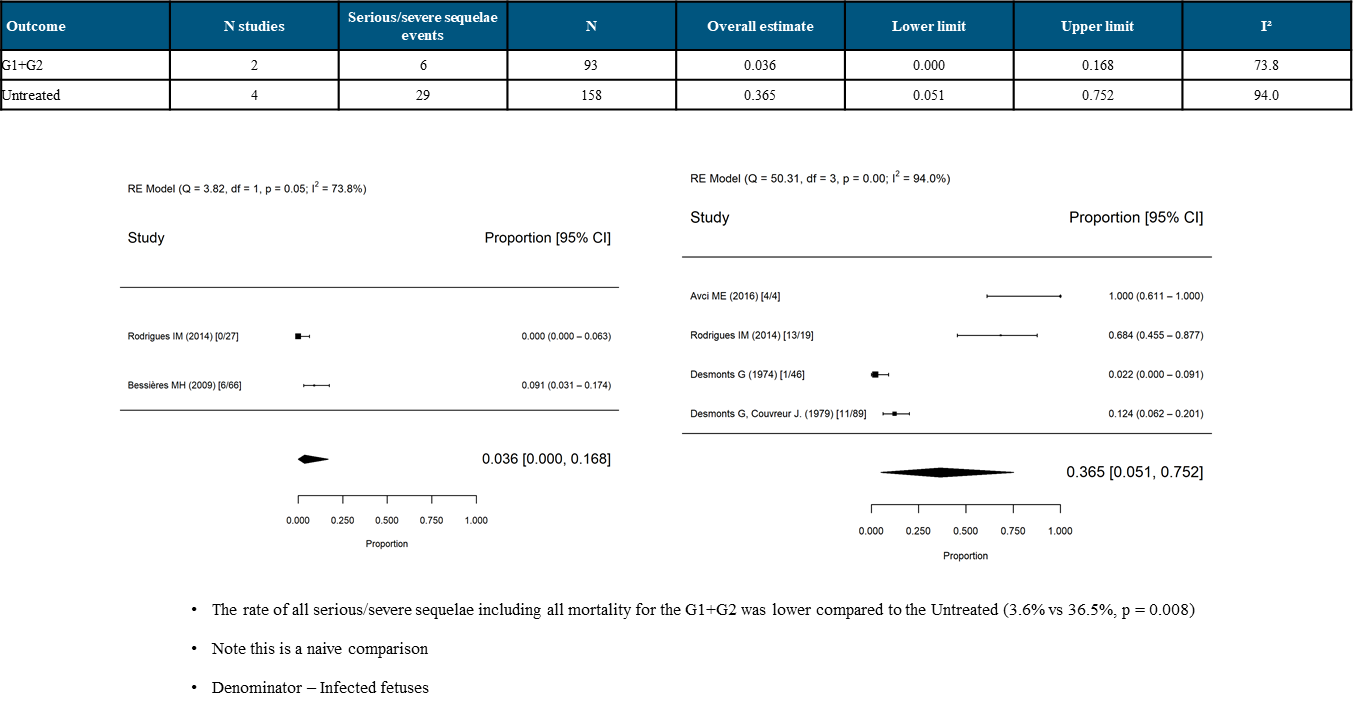


1. All Serious/Severe Sequelae and All Mortality, G1, 1974-2016 – Beyond 1 year


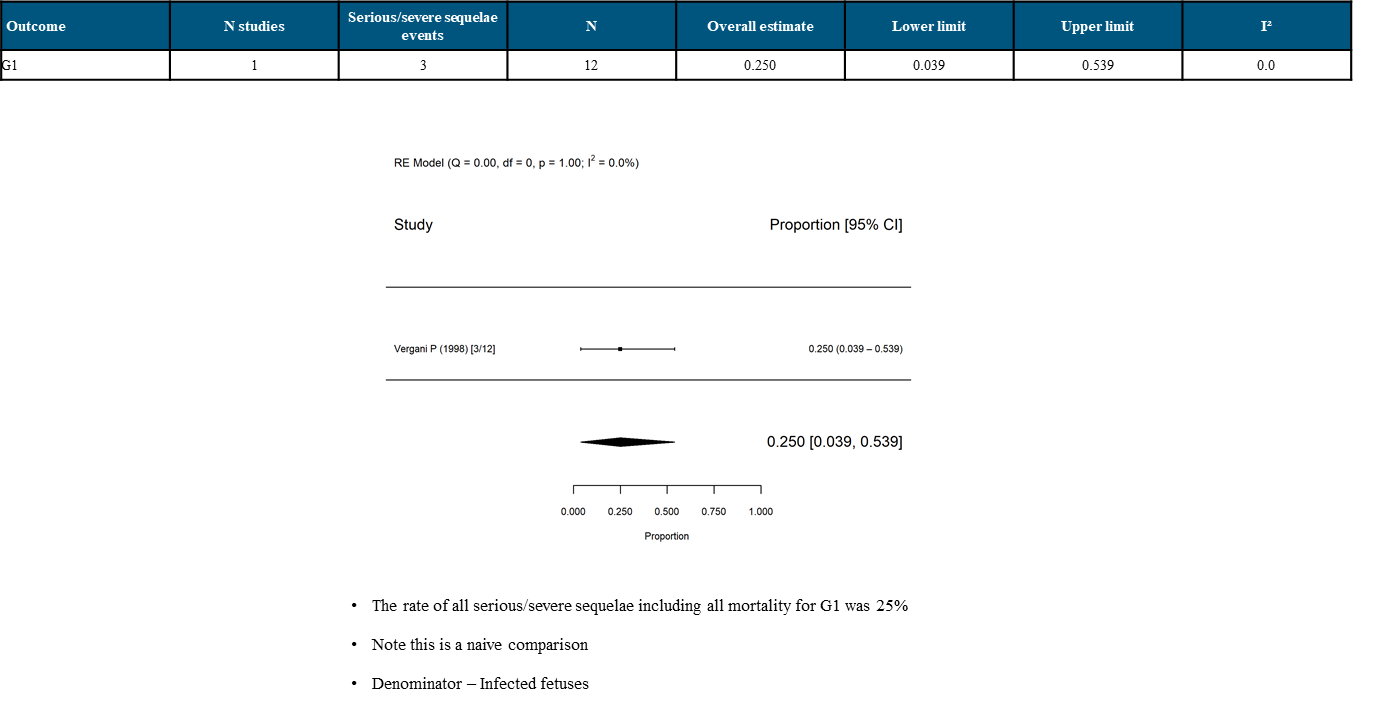


1. All Serious/Severe Sequelae and All Mortality, G1+G2, 1974-2016 – Beyond 1 year


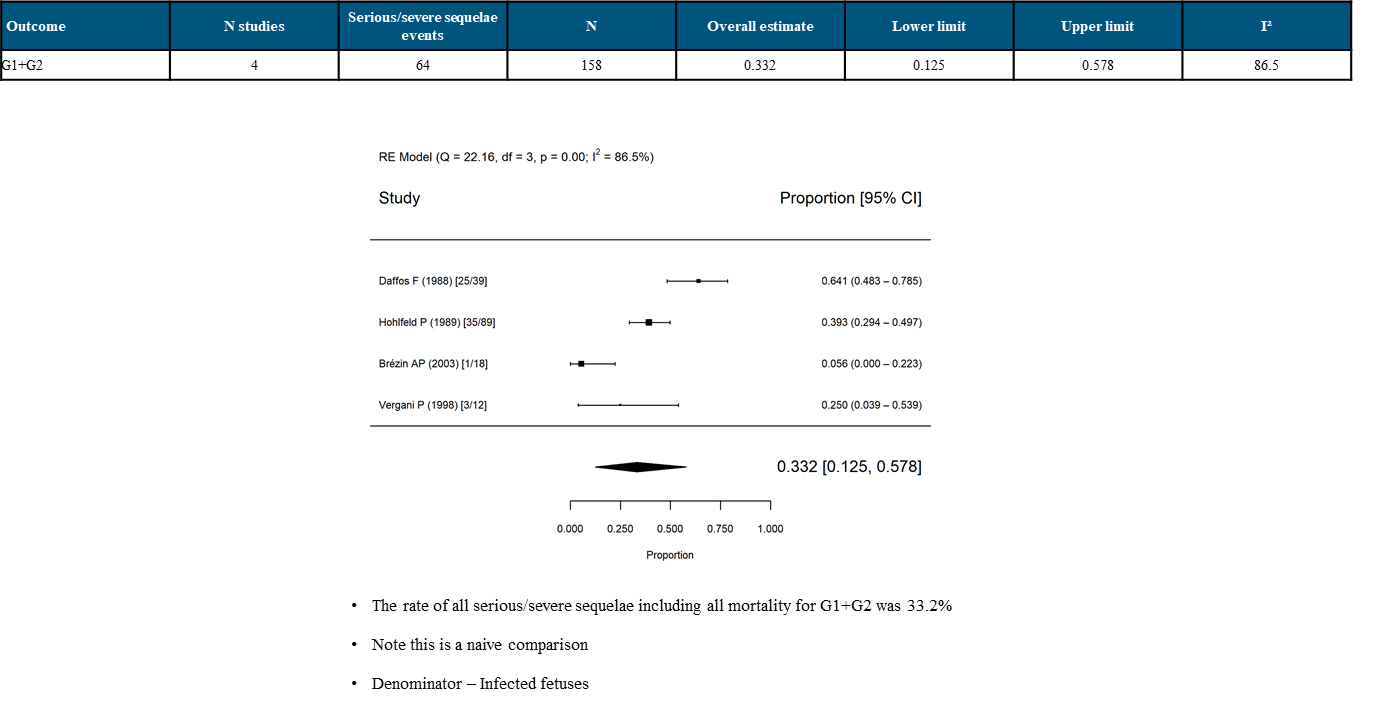


1. All Serious/Severe Sequelae and All Mortality, G1, Before 1999 – Beyond 1 year


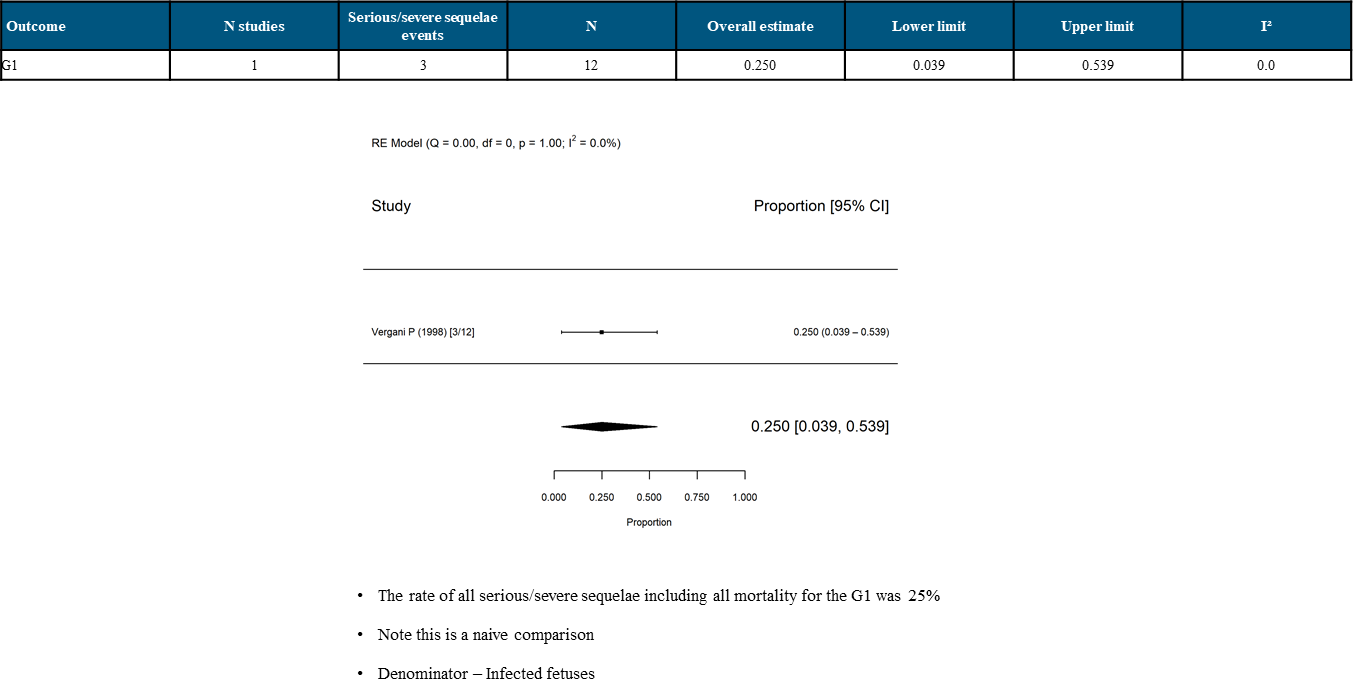


1. All Serious/Severe Sequelae and All Mortality, G1+G2, Before 1999 – Beyond 1 year


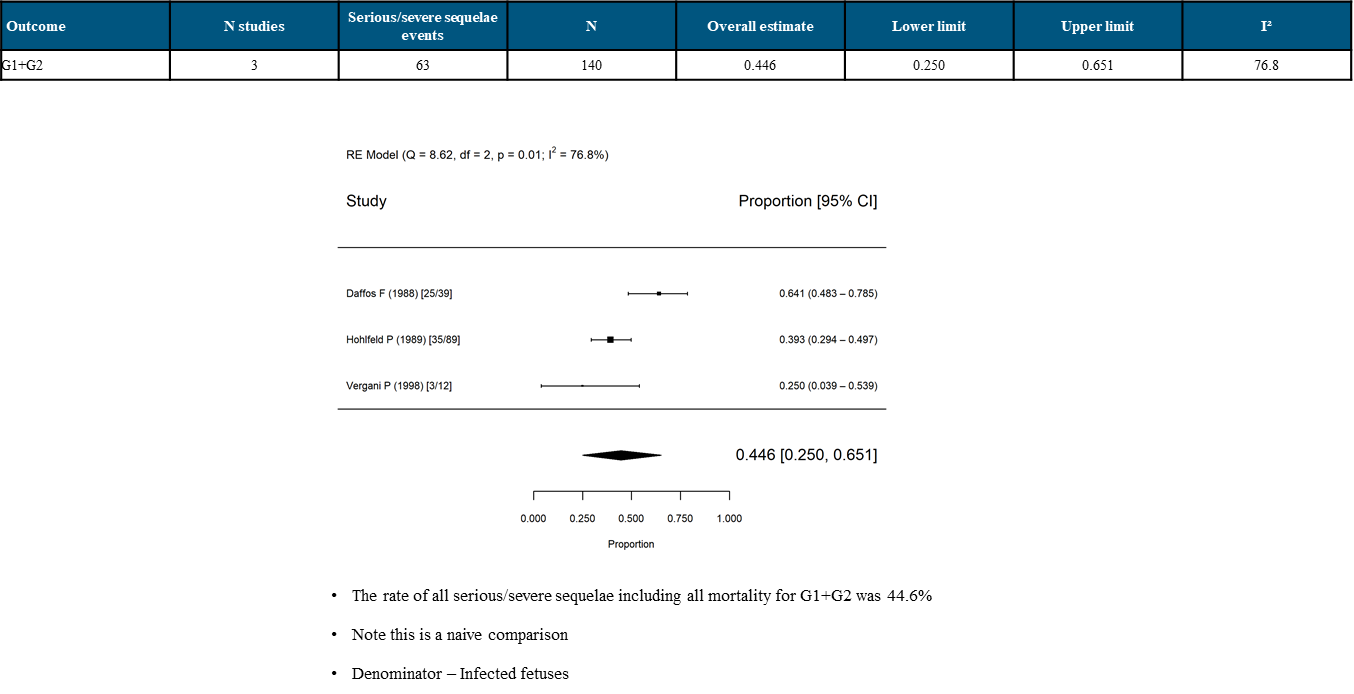


1. All Serious/Severe Sequelae and All Mortality, G1+G2, 1999-2006 – Beyond 1 year


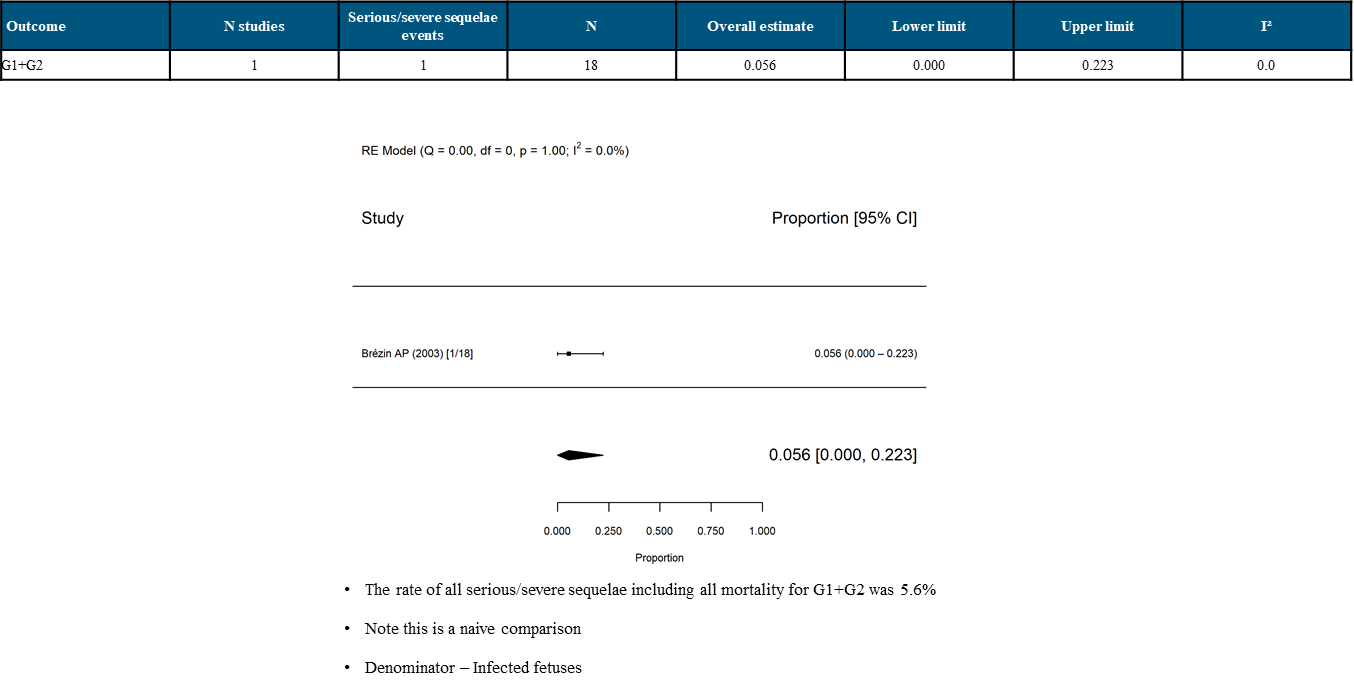


### Mild/Moderate/Severe Sequelae and Infant Mortality - Subgroup Analyses

1. All Mild/Moderate/Severe Sequelae and Infant Mortality (Postnatal), G1 vs Untreated, Before 1999 – Up to 1 year


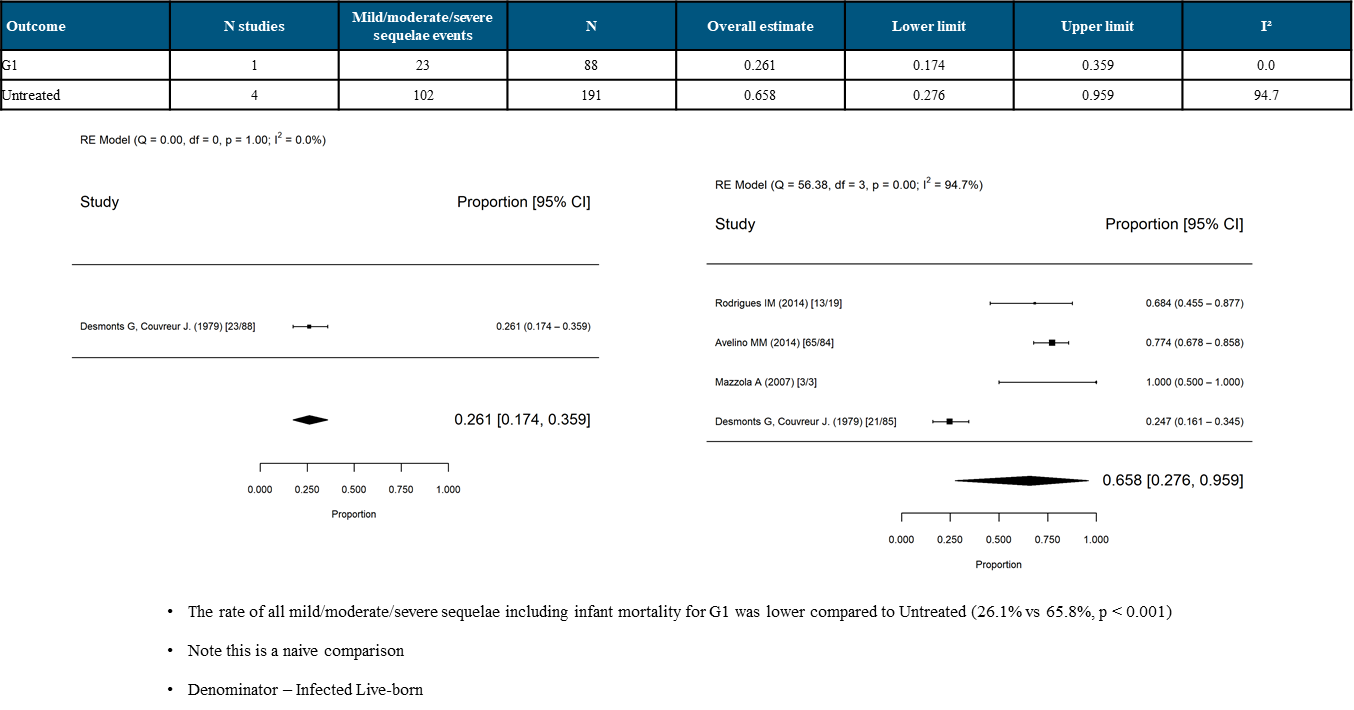


1. All Mild/Moderate/Severe Sequelae and Infant Mortality (Postnatal), G1+G2 vs Untreated, Before 1999 – Up to 1 year


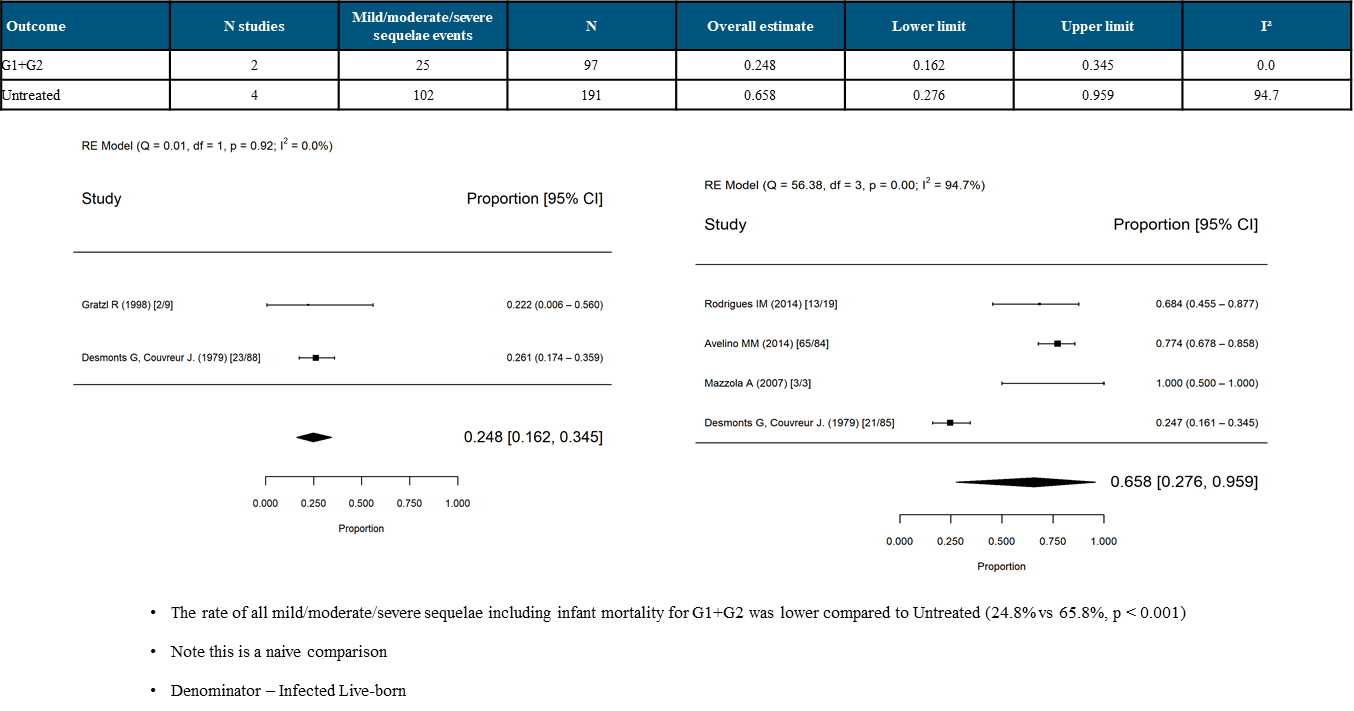


1. All Mild/Moderate/Severe Sequelae and Infant Mortality (Postnatal), G1+G2 vs Untreated, 1999-2006 – Up to 1 year


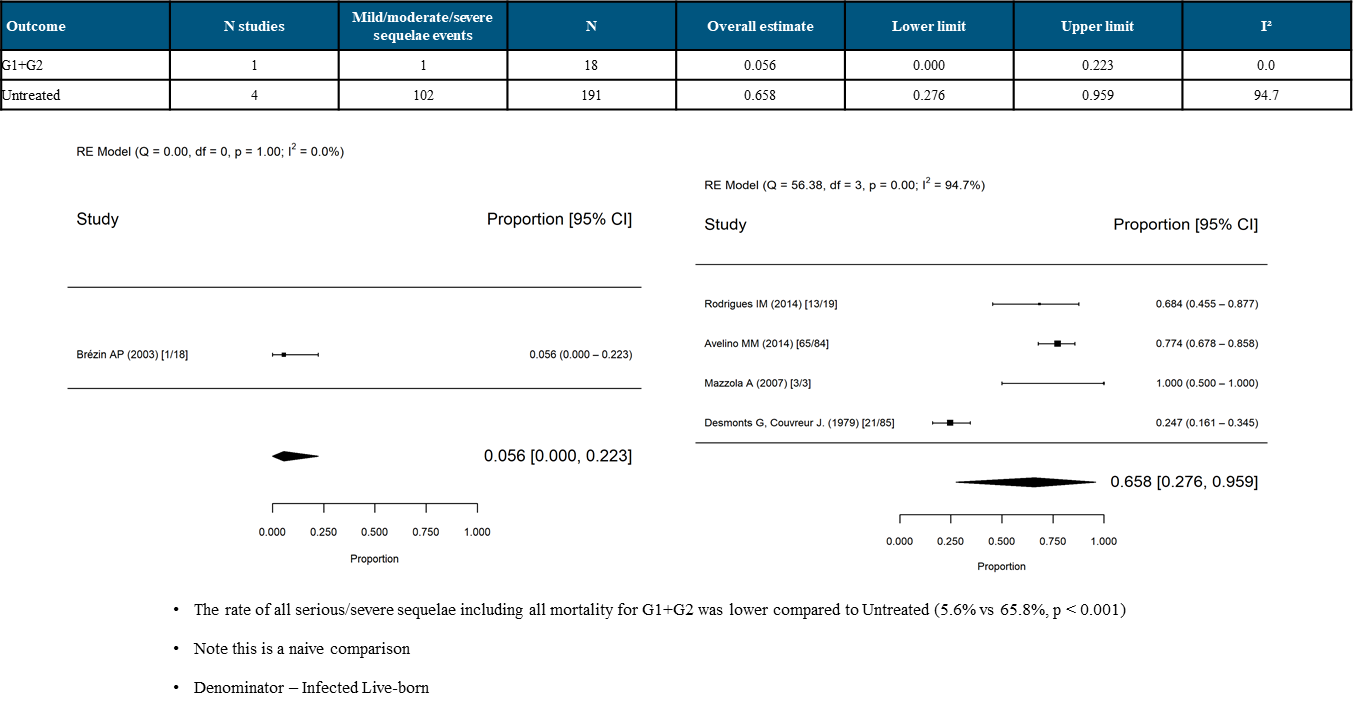


1. All Mild/Moderate/Severe Sequelae and Infant Mortality (Postnatal), G1 vs Untreated, After 2006 – Up to 1 year


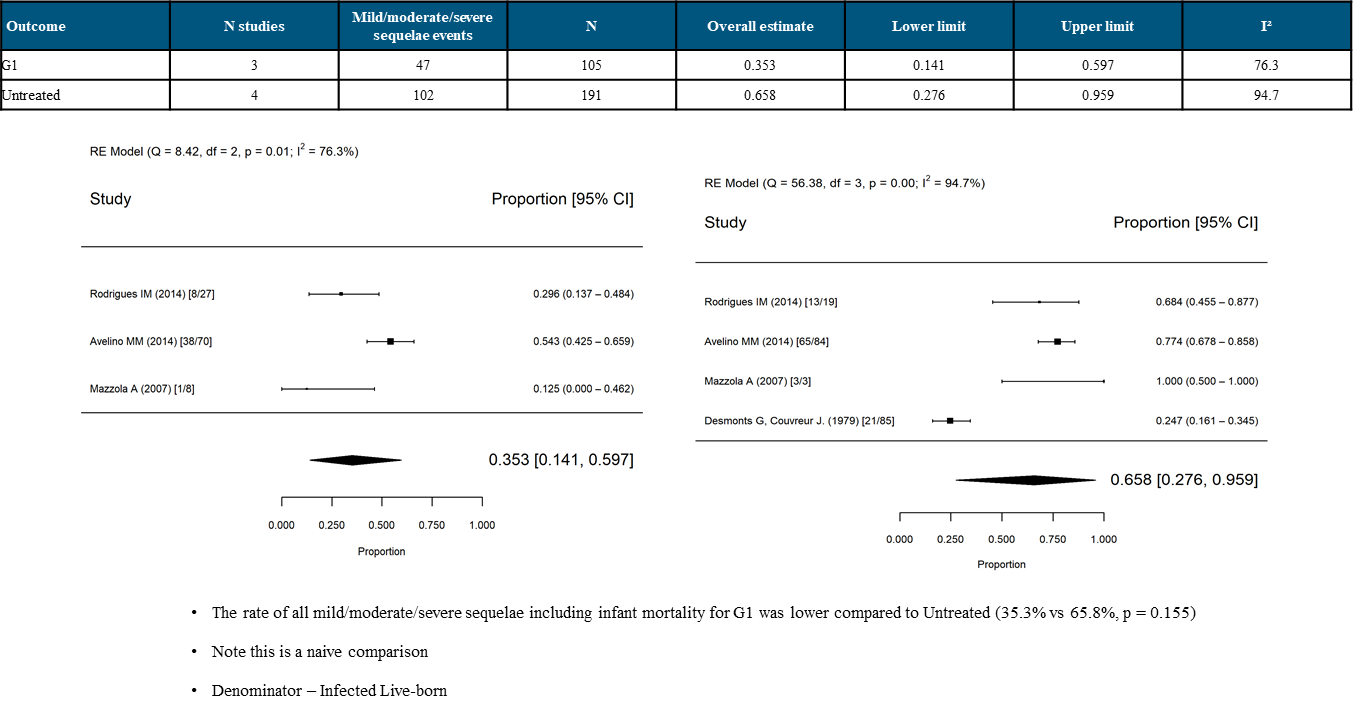


1. All Mild/Moderate/Severe Sequelae and Infant Mortality (Postnatal), G1+G2 vs Untreated, After 2006 – Up to 1 year


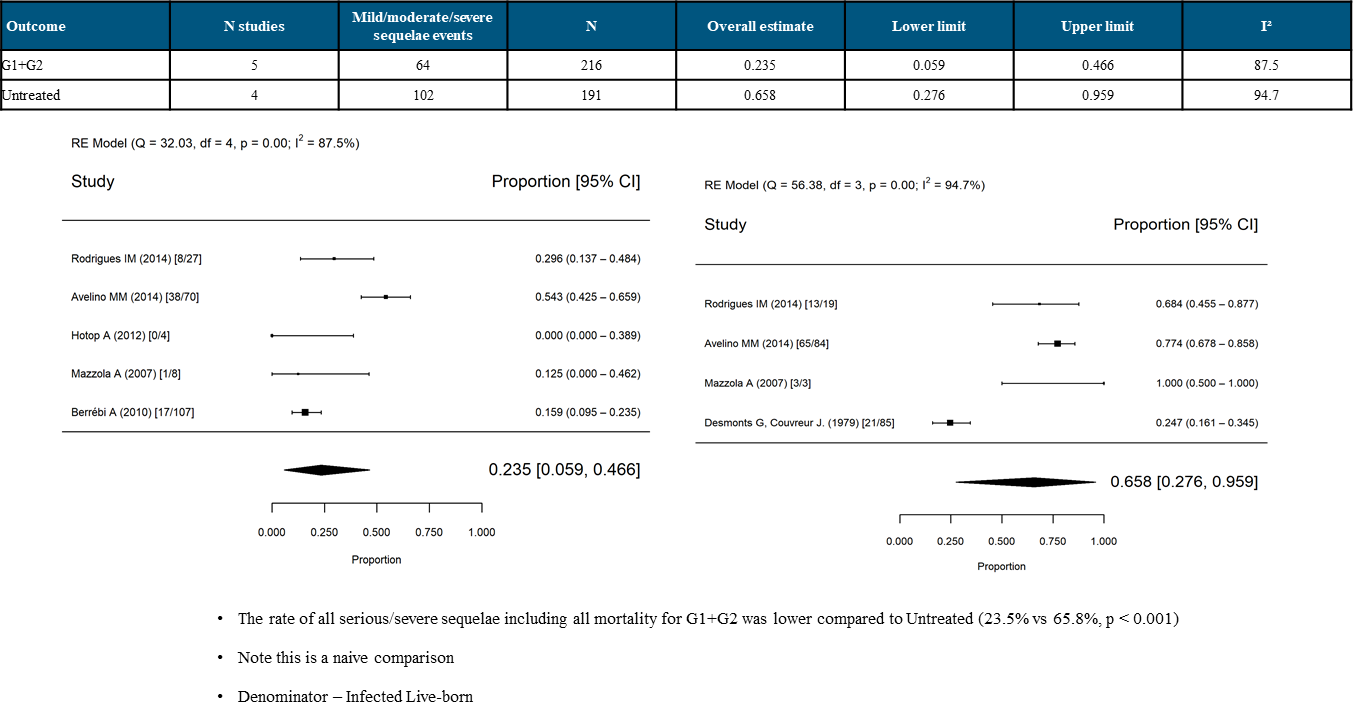


1. All Mild/Moderate/Severe Sequelae and Infant Mortality (Postnatal), G1, 1974-2016 – Beyond 1 year


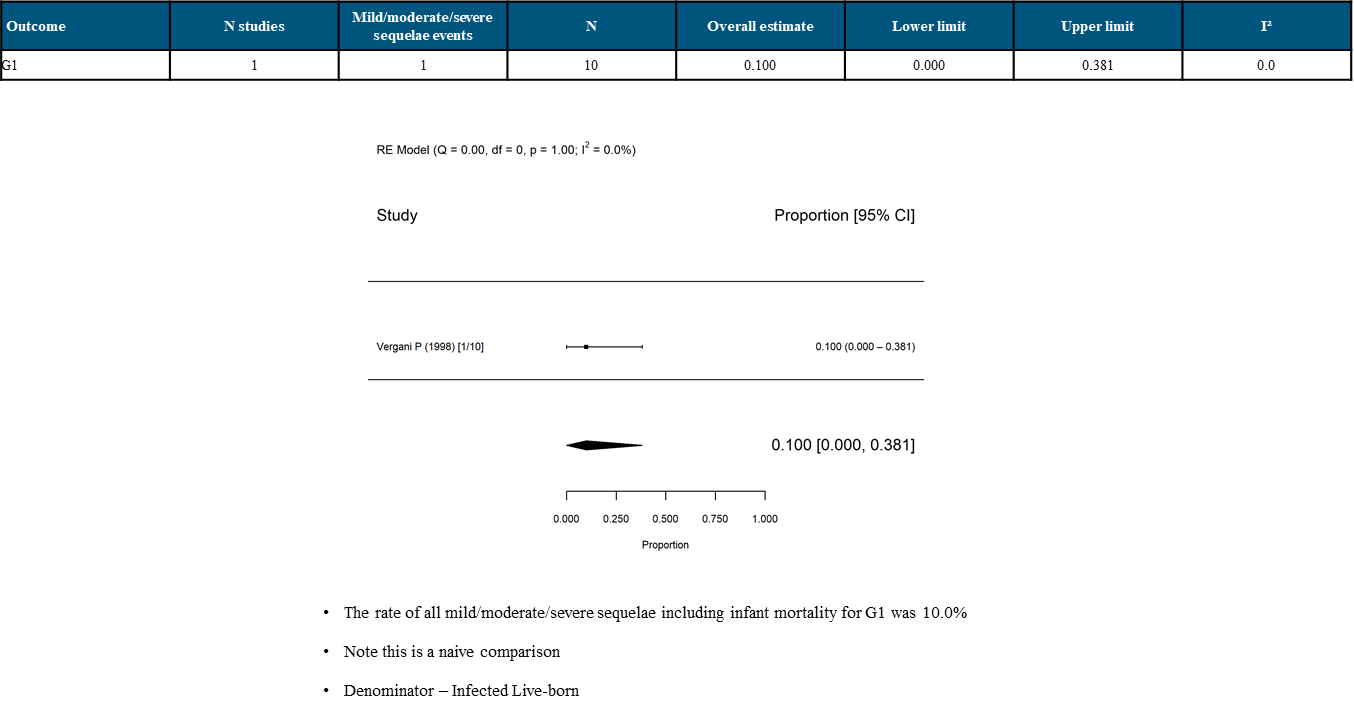


1. All Mild/Moderate/Severe Sequelae and Infant Mortality (Postnatal), G1+G2, 1974-2016 – Beyond 1 year


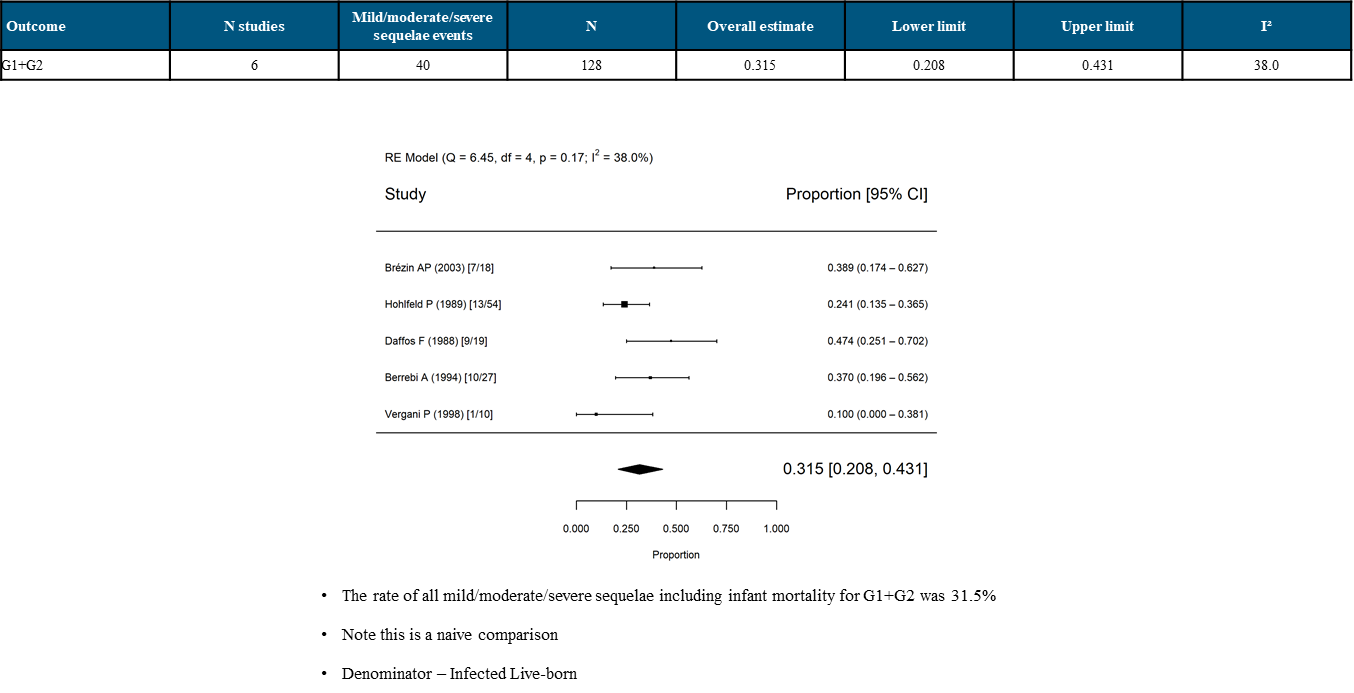


1. All Mild/Moderate/Severe Sequelae and Infant Mortality (Postnatal), G1, Before 1999 – Beyond 1 year


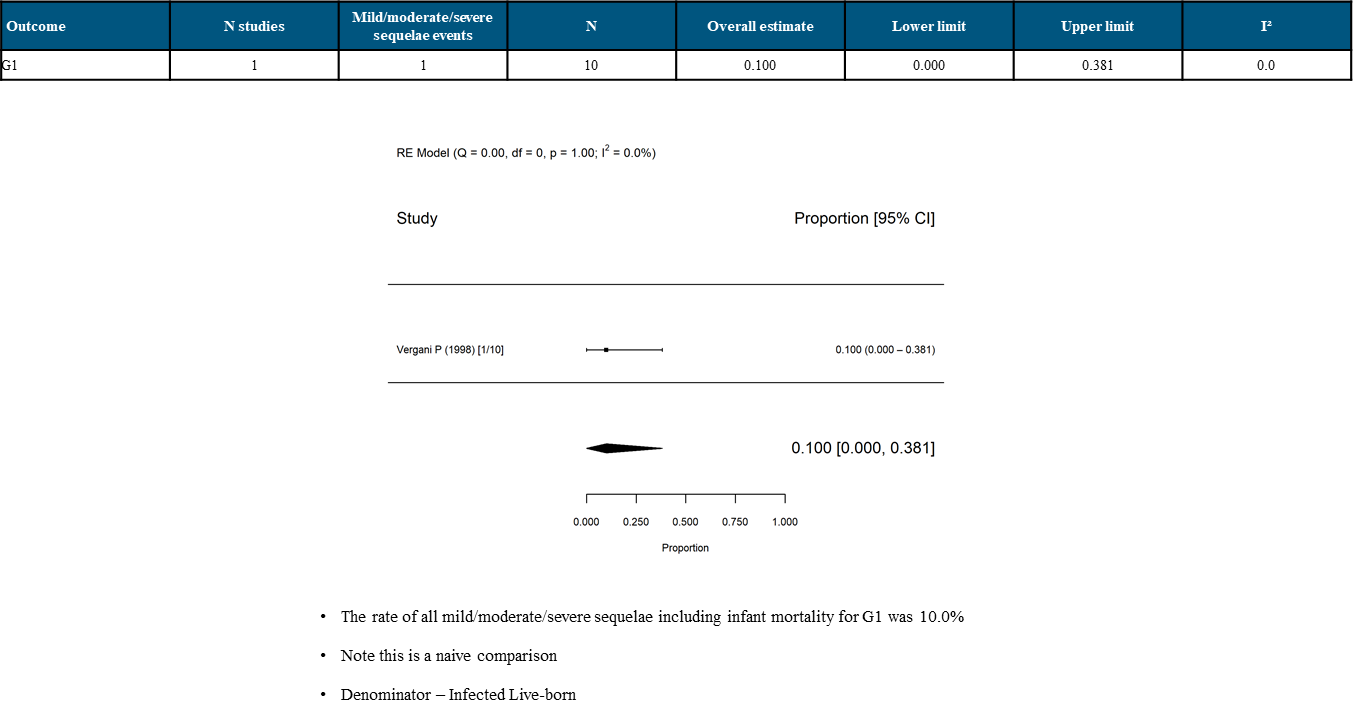


1. All Mild/Moderate/Severe Sequelae and Infant Mortality (Postnatal), G1+G2, Before 1999 – Beyond 1 year


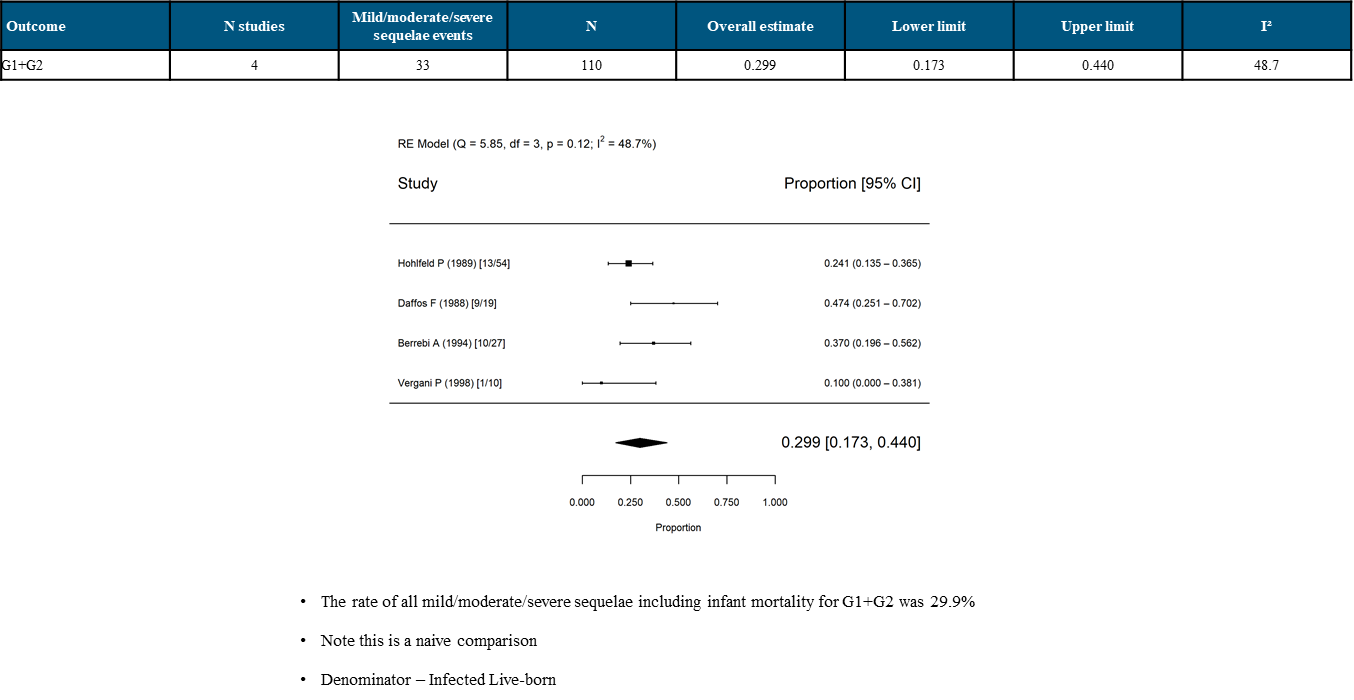


1. All Mild/Moderate/Severe Sequelae and Infant Mortality (Postnatal), G1+G2, 1999-2006 – Beyond 1 year


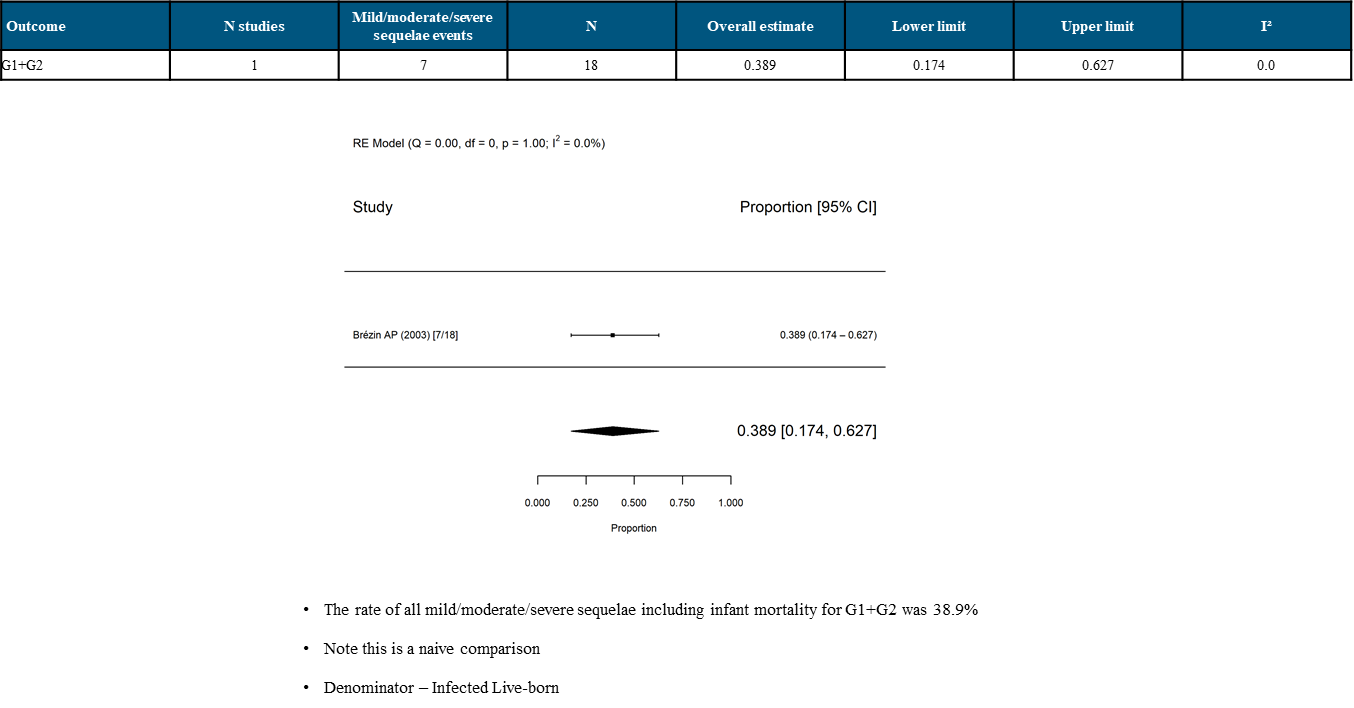


1. All Mild/Moderate/Severe Sequelae and Infant Mortality (Postnatal), G1+G2, After 2006 – Beyond 1 year


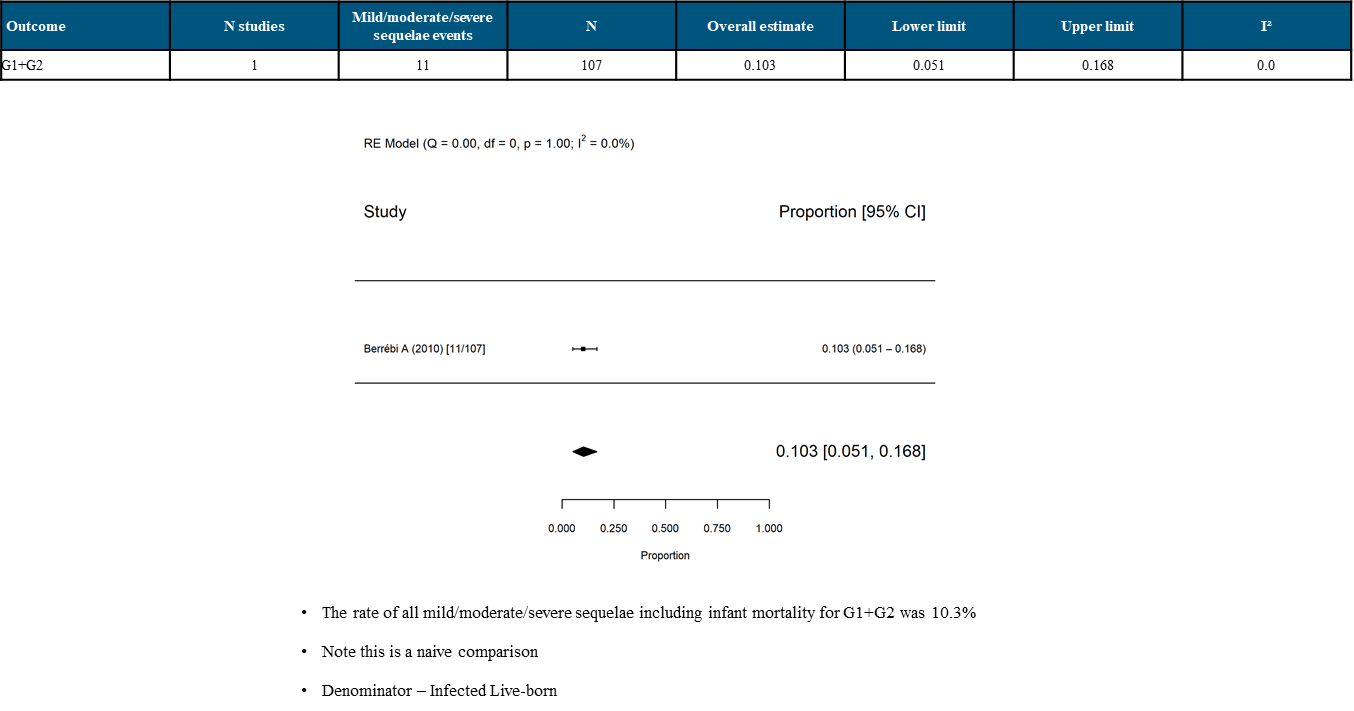


### Subclinical Disease - Subgroup Analyses

1. Subclinical Disease, G1 vs Untreated, Before 1999 – Up to 1 year


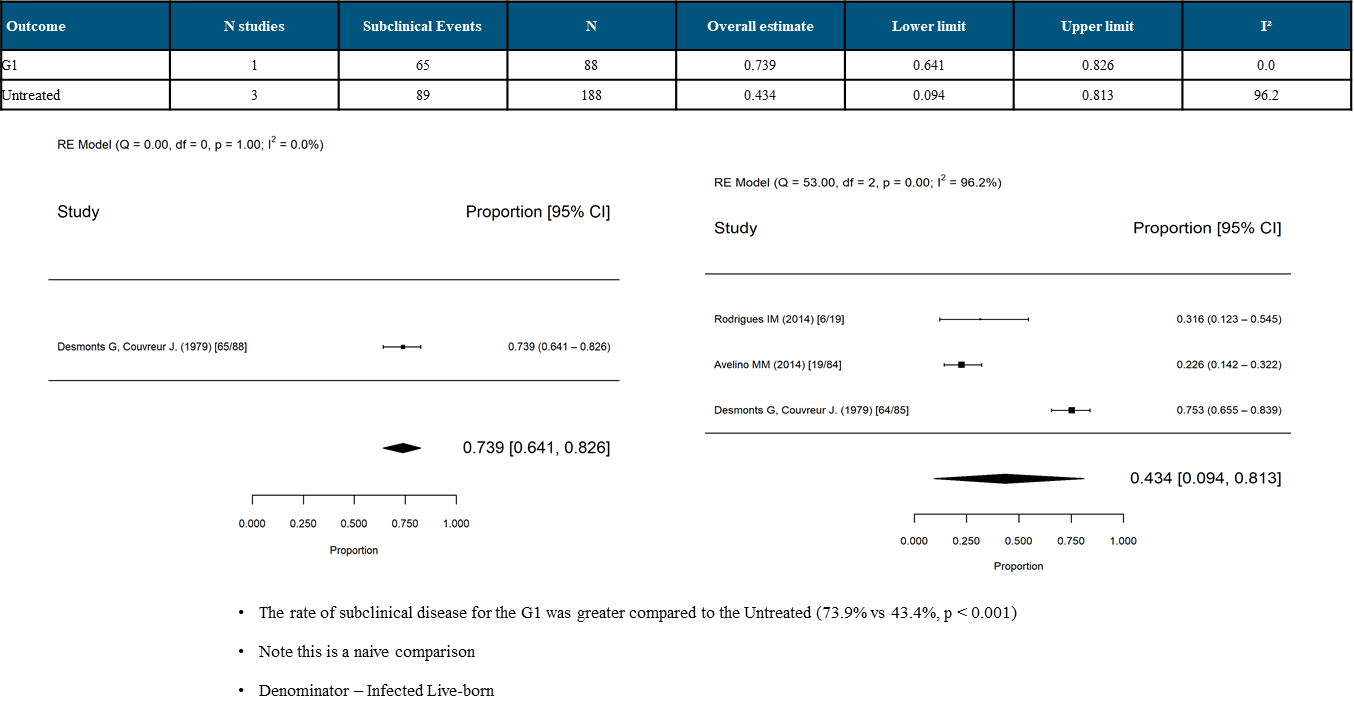


1. Subclinical Disease, G1+G2 vs Untreated, Before 1999 – Up to 1 year


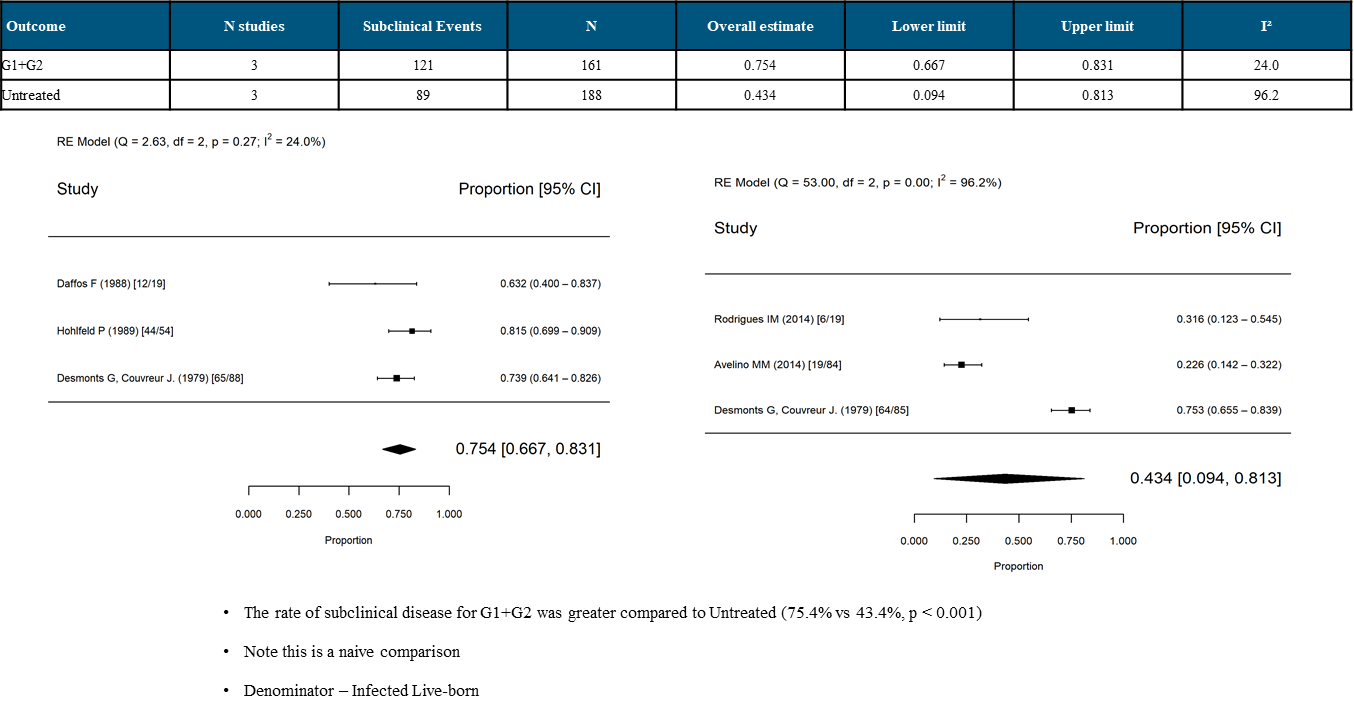


1. Subclinical Disease, G1 vs Untreated, After 2006 – Up to 1 year


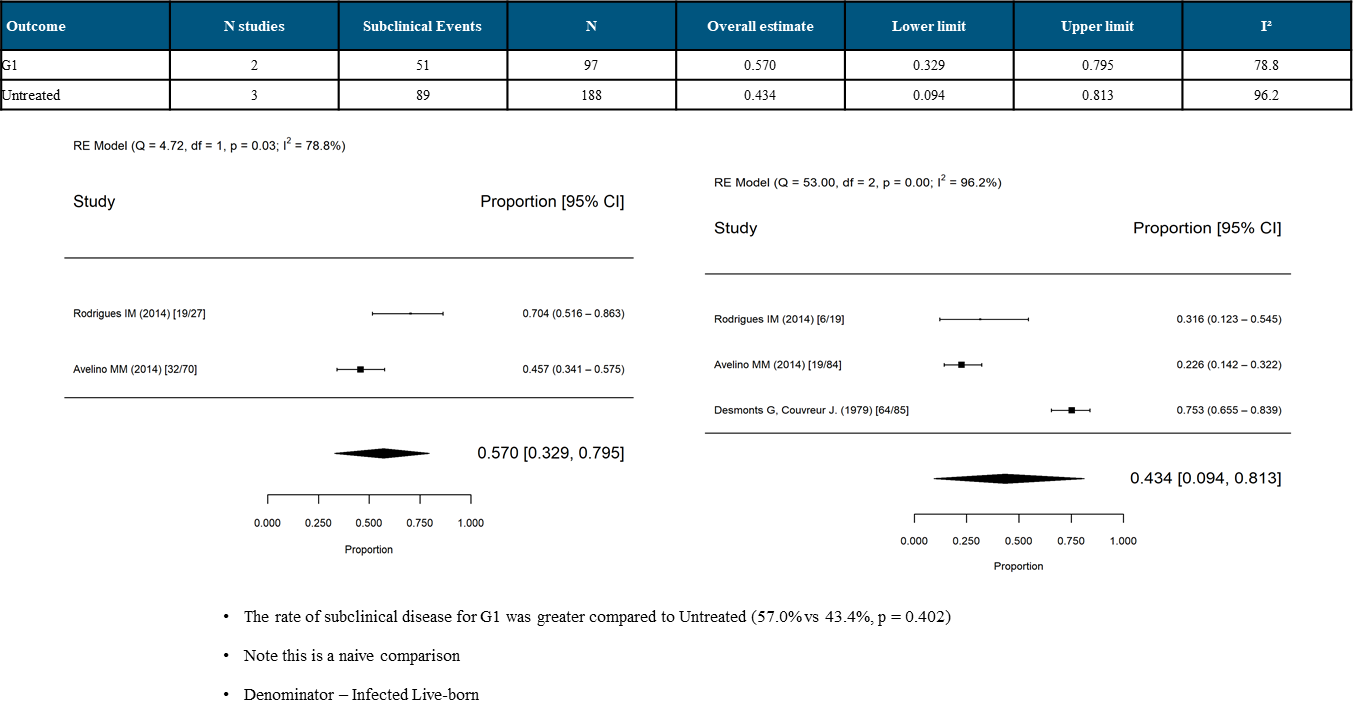


1. Subclinical Disease, G1+G2 vs Untreated, After 2006 – Up to 1 year


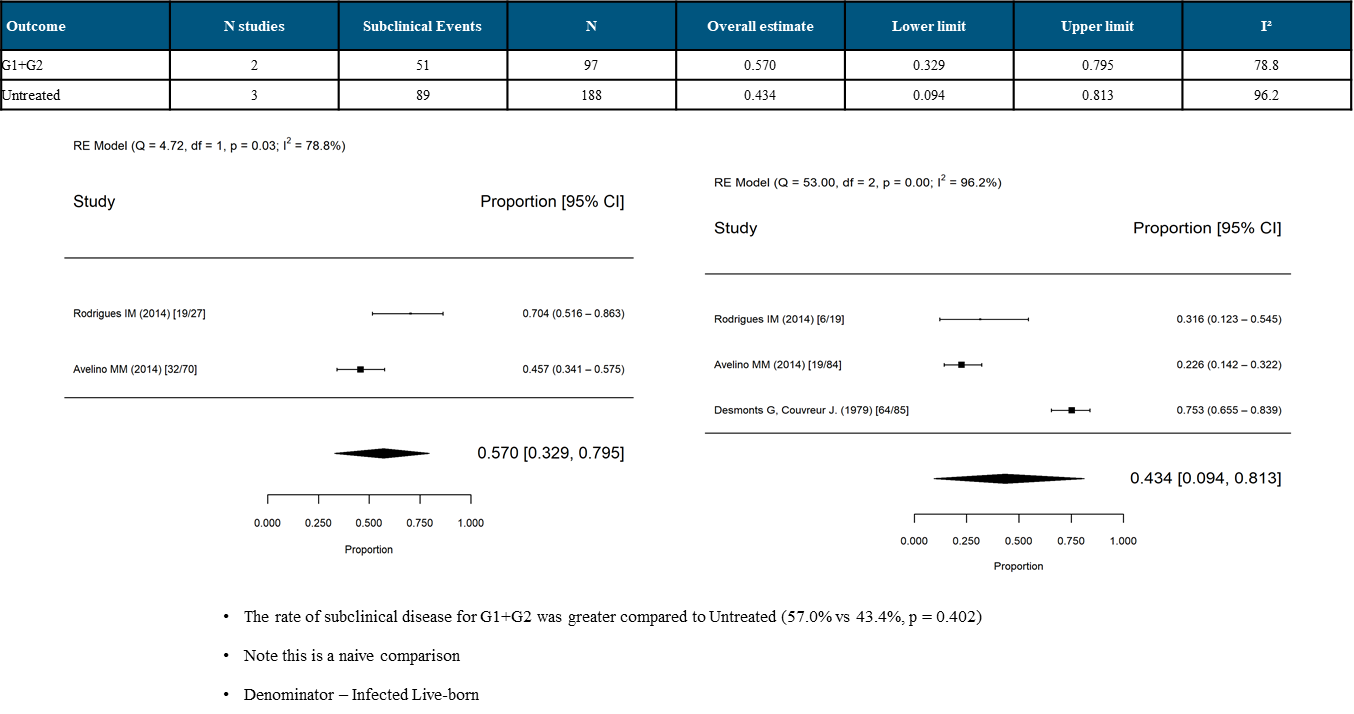


1. Subclinical Disease, G1, 1974-2016 – Beyond 1 year


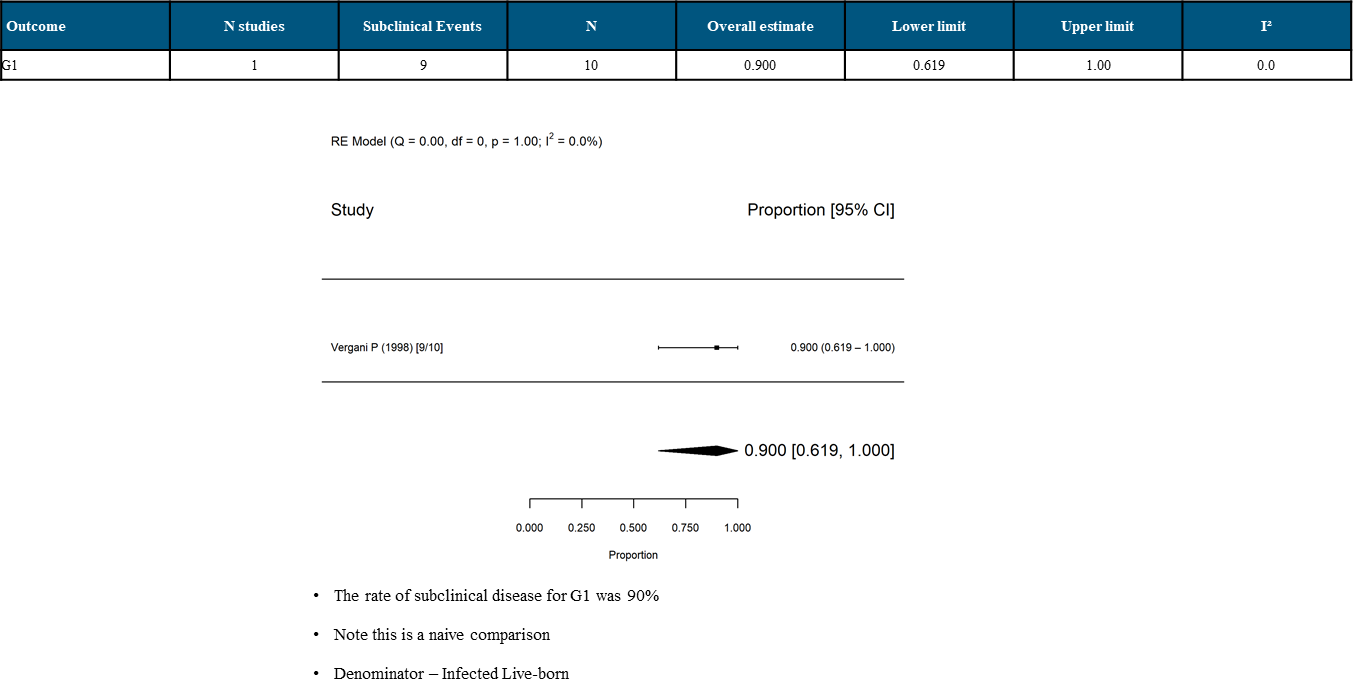


1. Subclinical Disease, G1+G2, 1974-2016 – Beyond 1 year


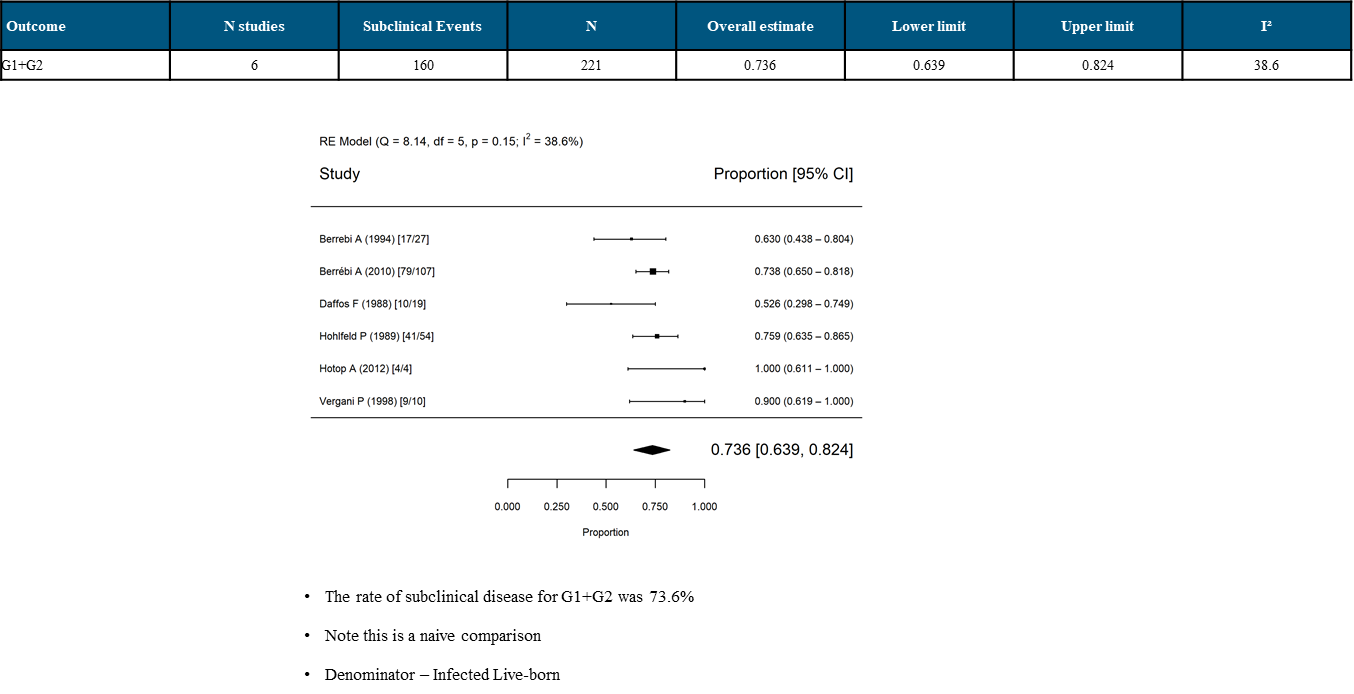


1. Subclinical Disease, G1, Before 1999 – Beyond 1 year


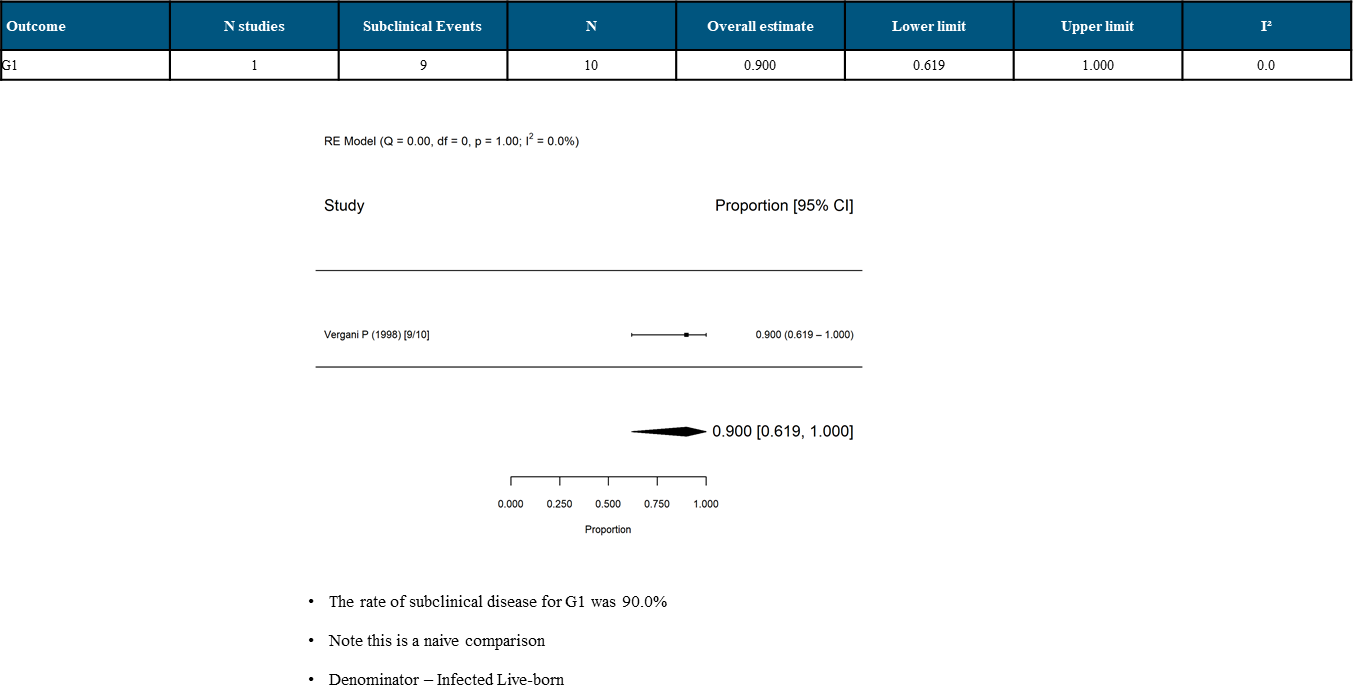


1. Subclinical Disease, G1+G2, Before 1999 – Beyond 1 year


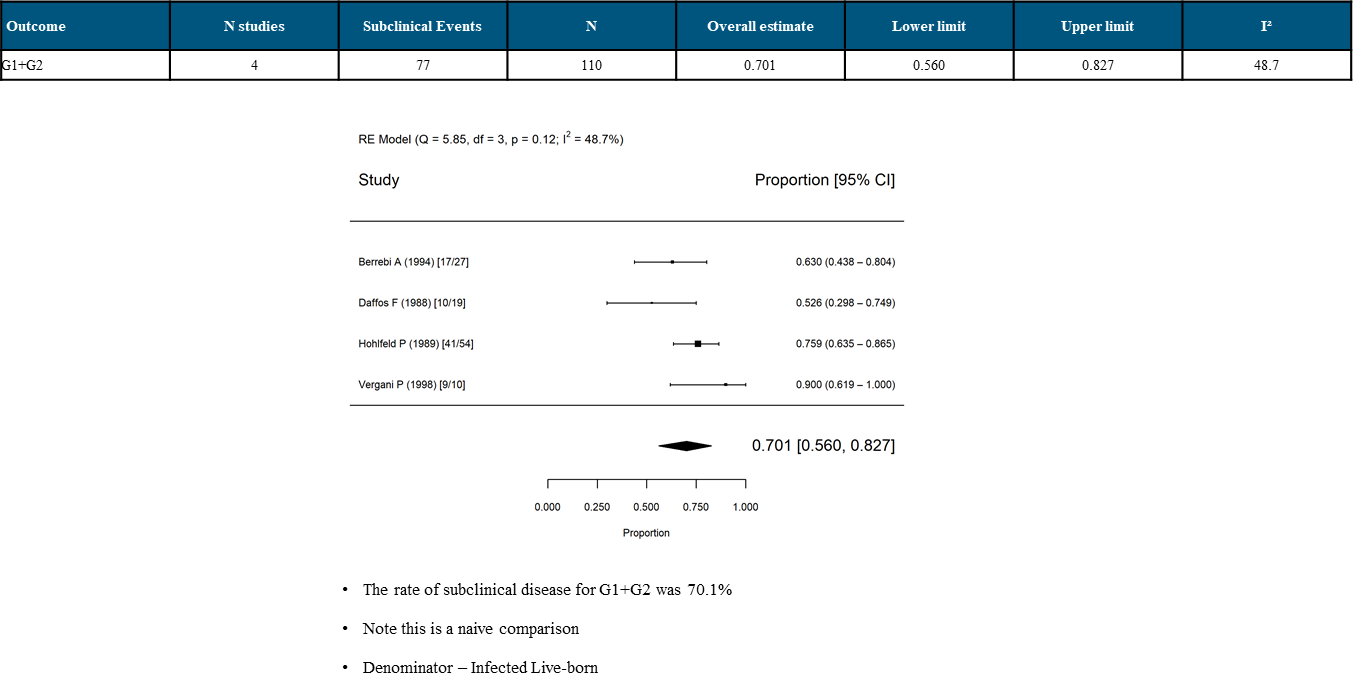


1. Subclinical Disease, G1+G2, After 2006 – Beyond 1 year


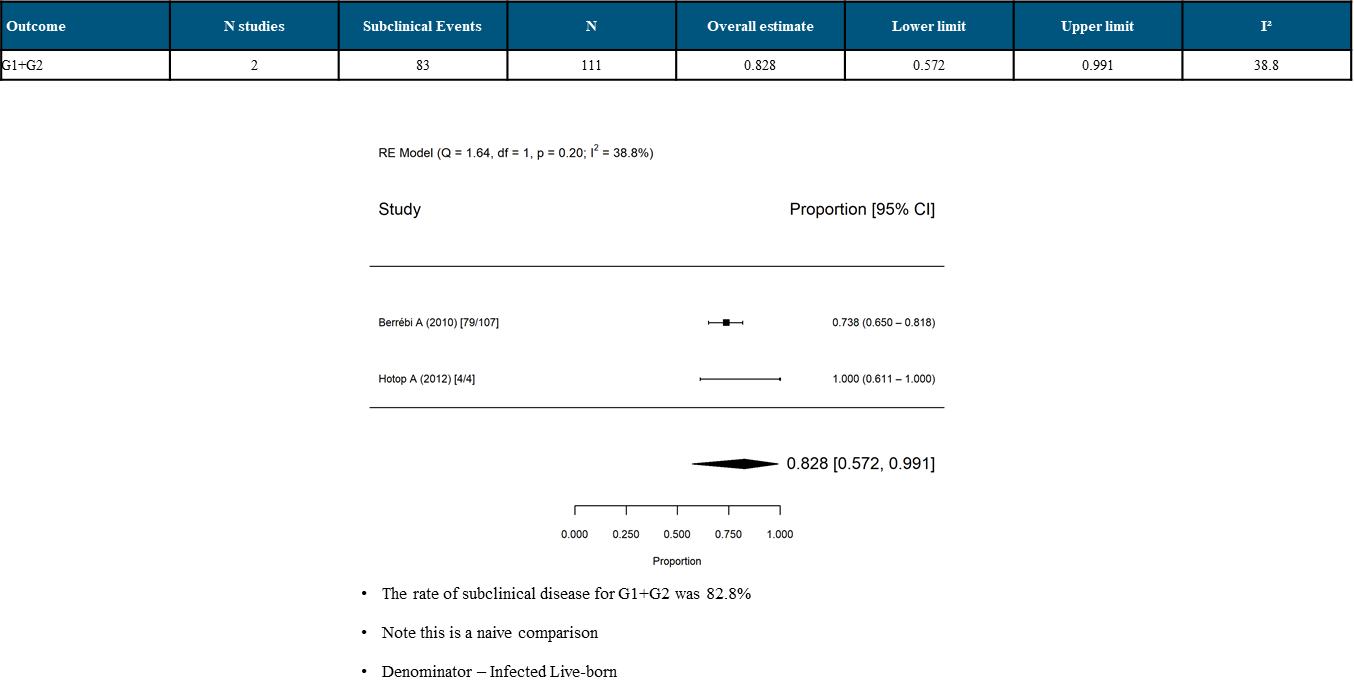


### Chorioretinitis Subgroup - Analyses

1. Chorioretinitis, G1 vs Untreated, After 2006 – Up to 1 year


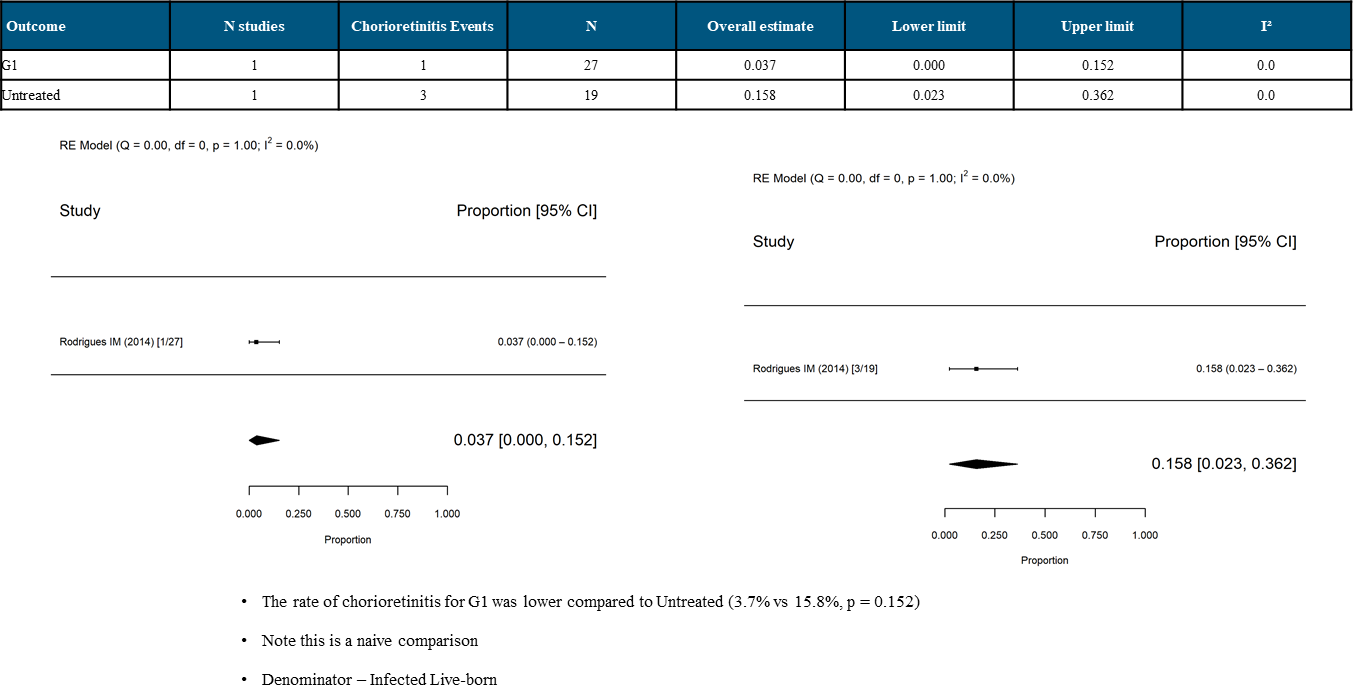


1. Chorioretinitis, G1+G2 vs Untreated, After 2006 – Up to 1 year


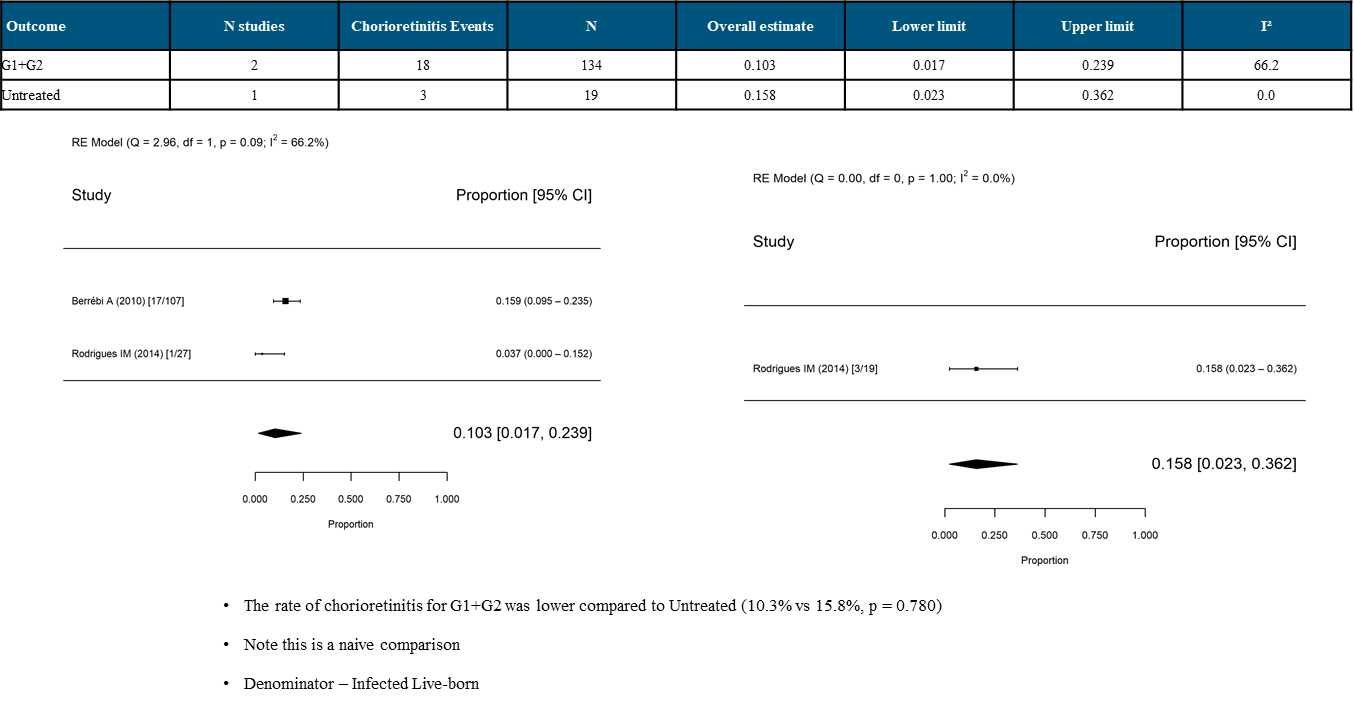


1. Chorioretinitis, G1 vs Untreated, 1974-2016 – Beyond 1 year


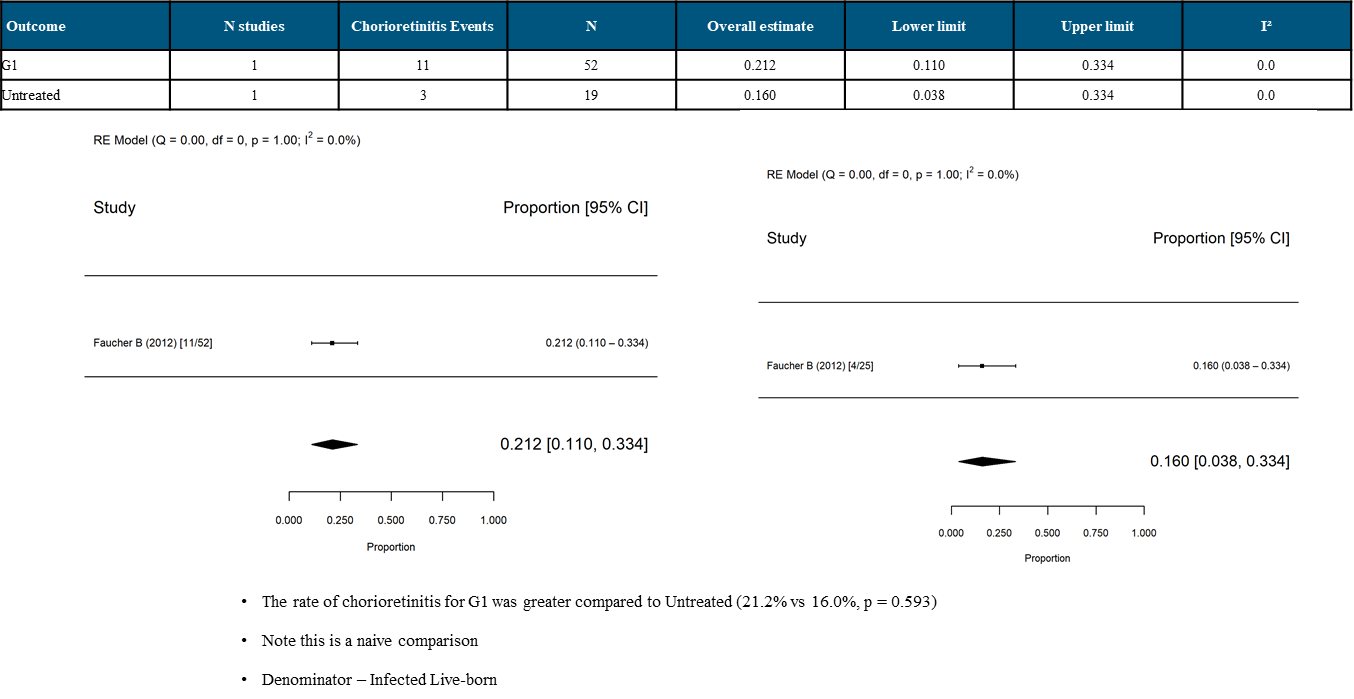


1. Chorioretinitis, G1+G2 vs Untreated, 1974-2016 – Beyond 1 year


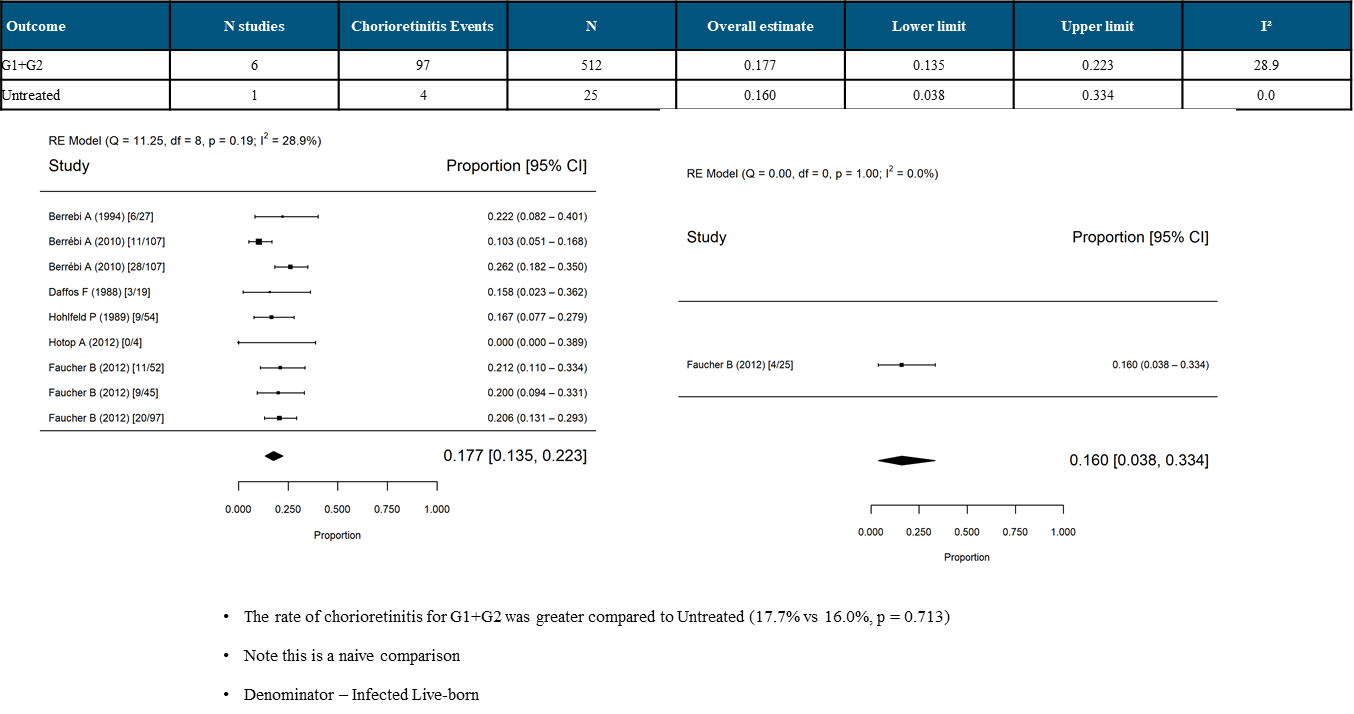


1. Chorioretinitis, G1+G2 vs Untreated, Before 1999 – Beyond 1 year


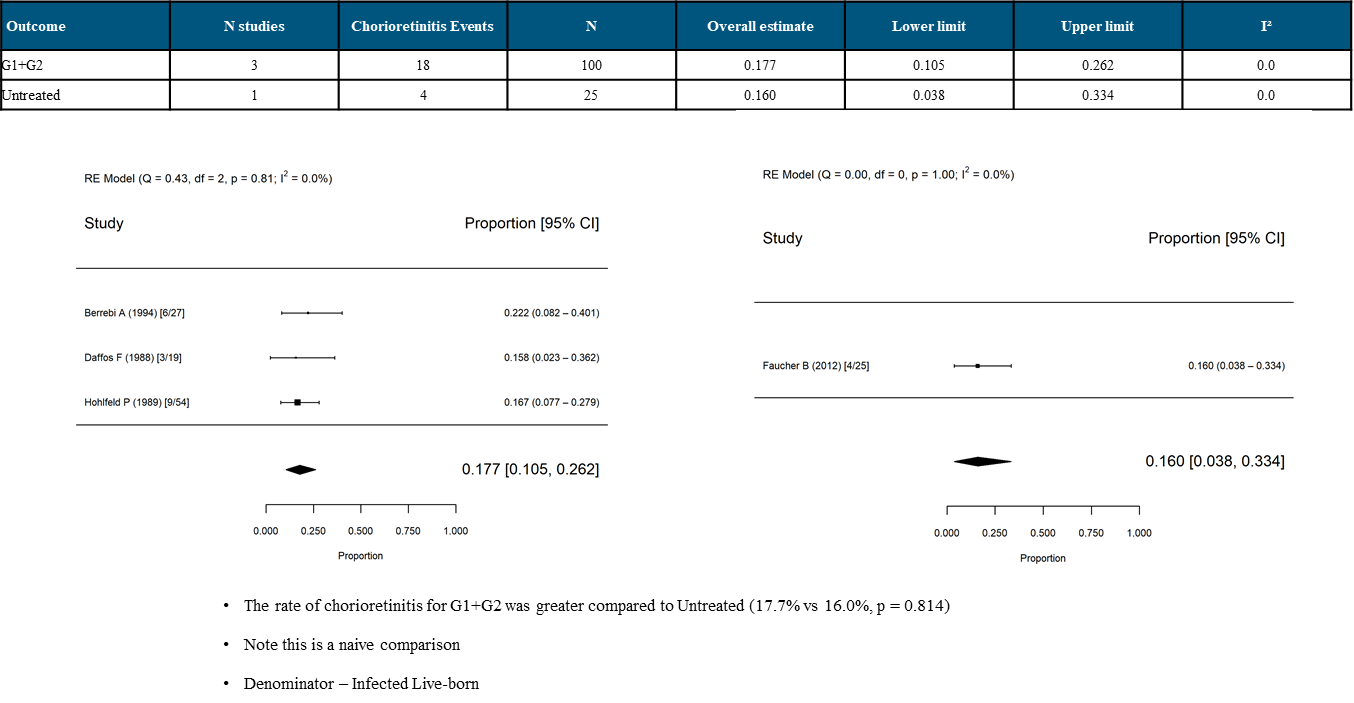


1. Chorioretinitis, G1 vs Untreated, After 2006 – Beyond 1 year


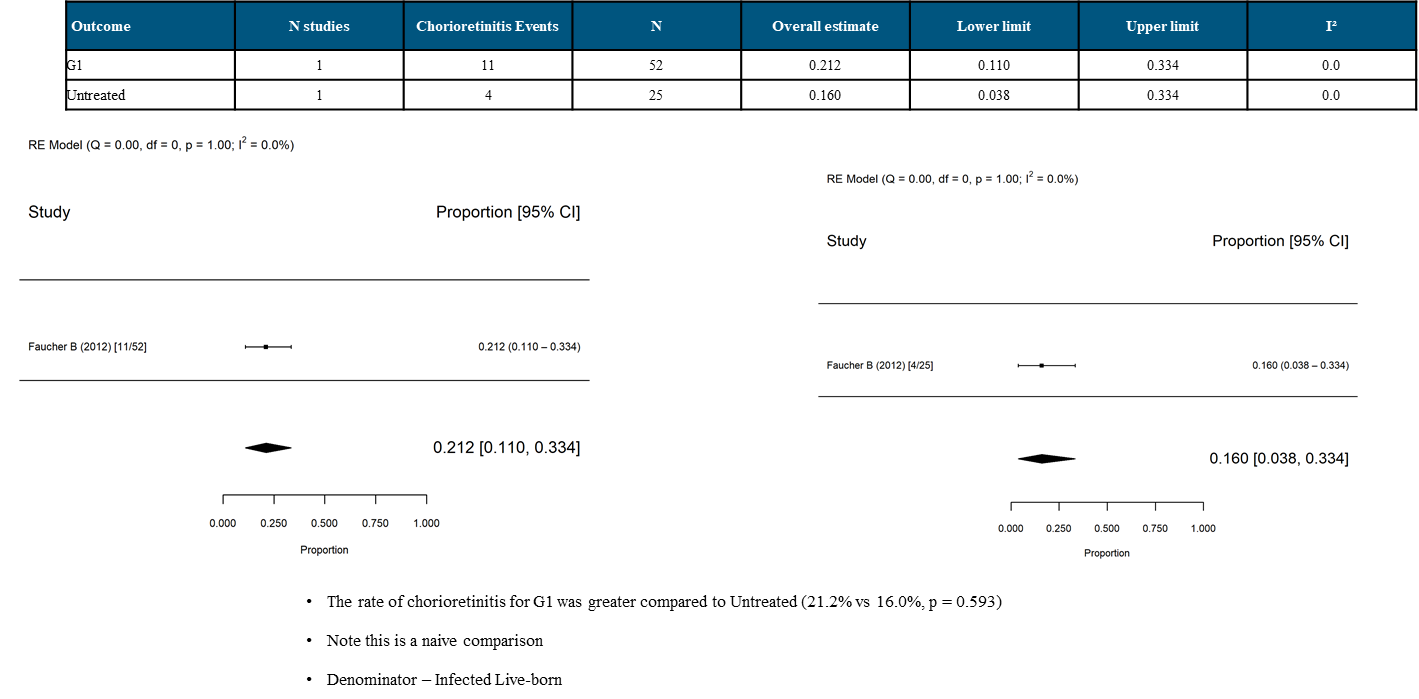


1. Chorioretinitis, G1+G2 vs Untreated, After 2006 – Beyond 1 year


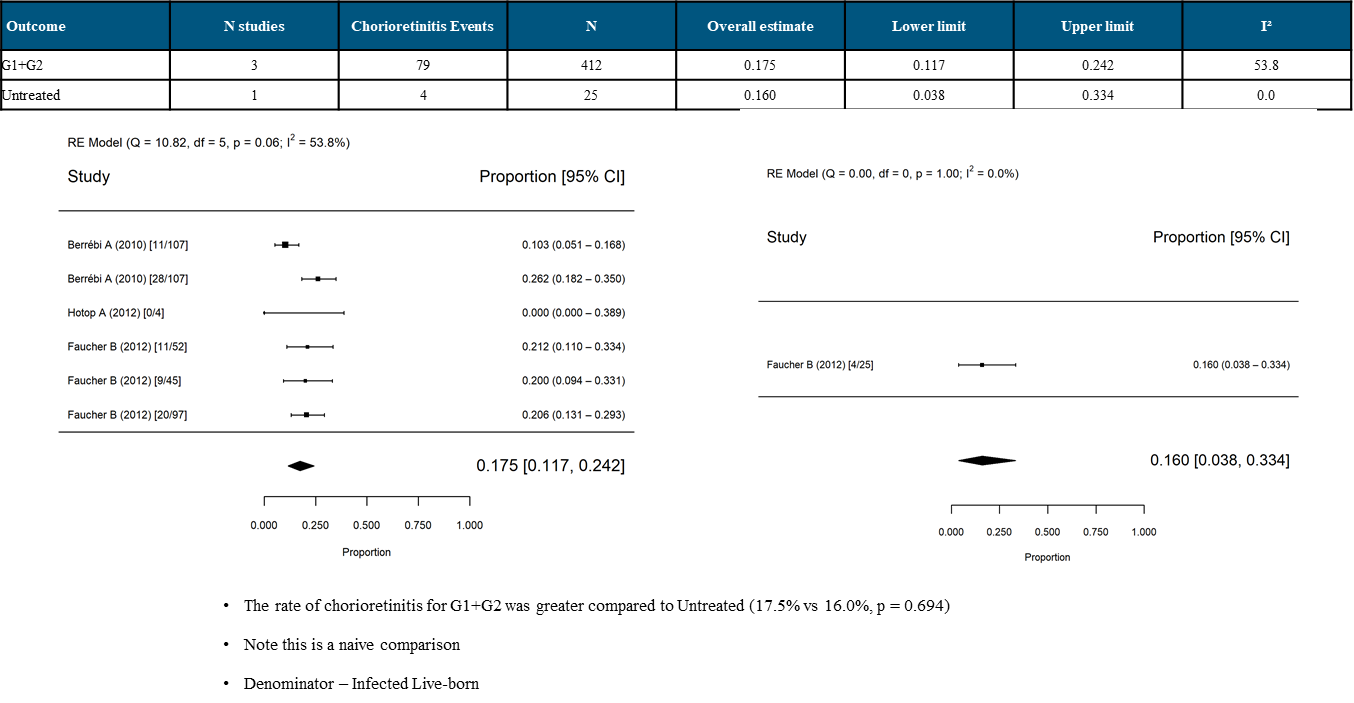


## Sensitivity Analyses

Avelino 2014 was excluded in a sensitivity analysis due to the South American cohort’s highly virulent *T. gondii* strain, which increased the MTCT rate (**Figure S77-S78**). When MTCT was calculated for the remaining 19 studies conducted in 1974-2016 [[1-18](#_ENREF_1)], the mean rates were 14.3% (95% CI: 8.4-21.4%) for spiramycin monotherapy, 17.9% (95% CI: 12.9-23.6%)[[1-18](#_ENREF_1)][[1-18](#_ENREF_1)]^1-185,7,9-11,13,16,17,20,21,23,24,28-33^ for spiramycin and/or PSF, and 47.7% (95% CI: 27.4-68.3%) for the untreated (p<0.001 for both treated versus untreated comparisons).

Among the 33 included studies, 15 studies were identified as meeting the prespecified criteria for sensitivity analyses whose are provided in **Figure S79-S108**. MTCT was lower in any group that received treatment compared to the untreated group across all trimesters.

### MTCT - Sensitivity Analyses

1. MTCT, G1 vs Untreated, Excluding Avelino 2014


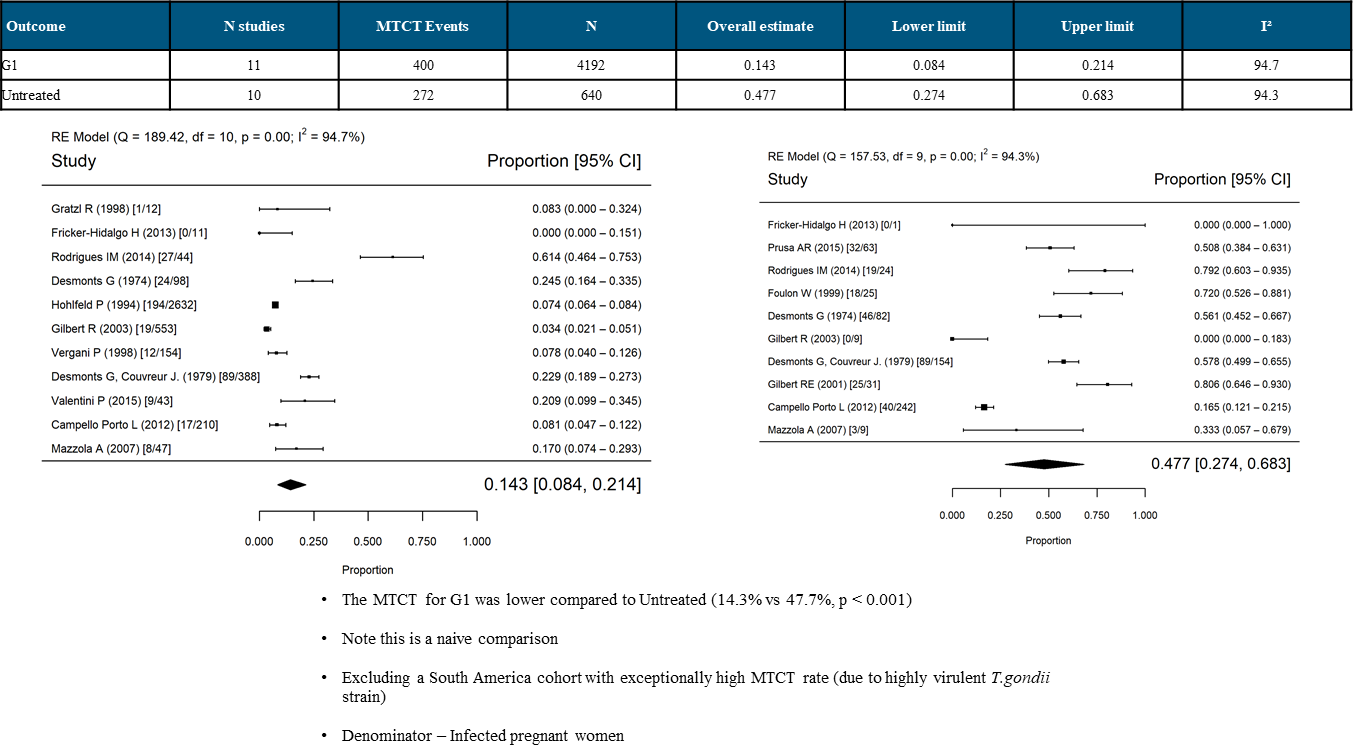


1. MTCT, G1+G2 vs Untreated, Excluding Avelino 2014


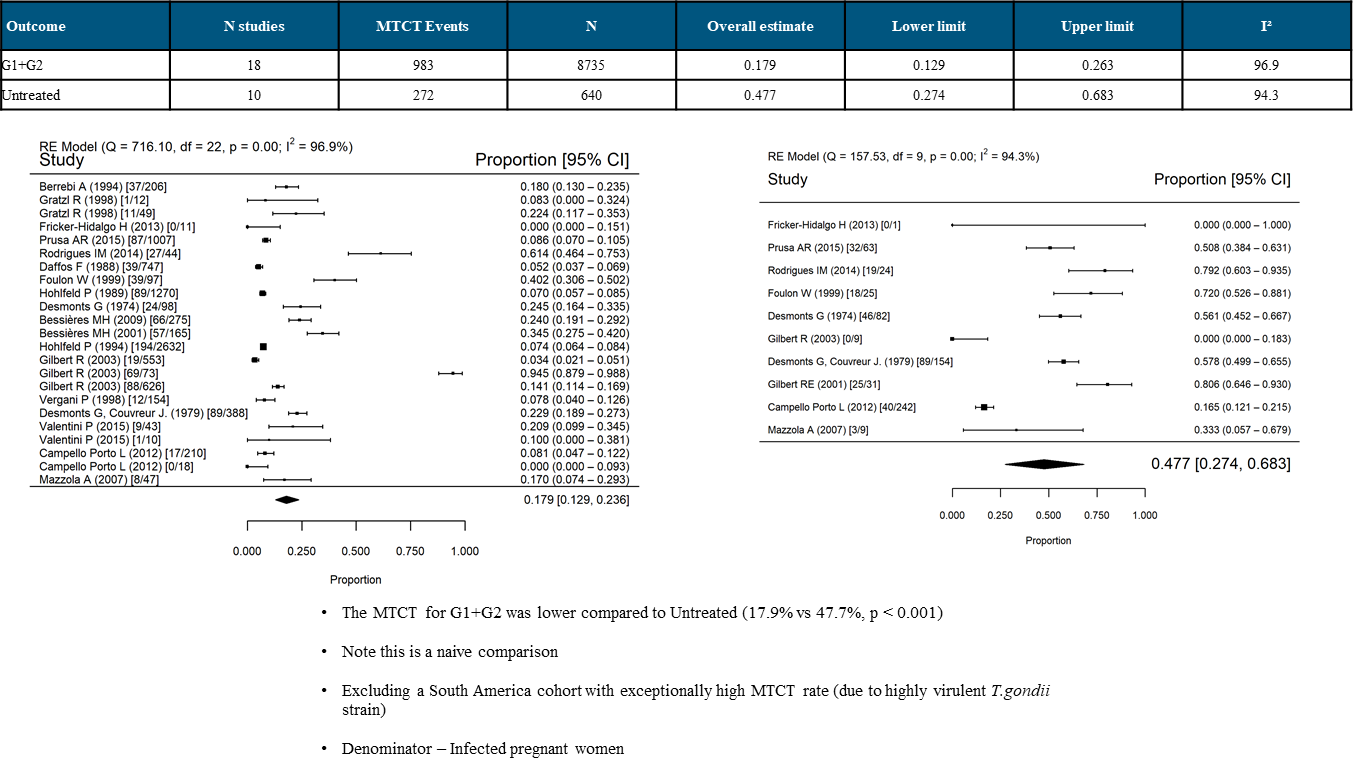


1. MTCT, G1 vs Untreated, Sensitivity Analysis


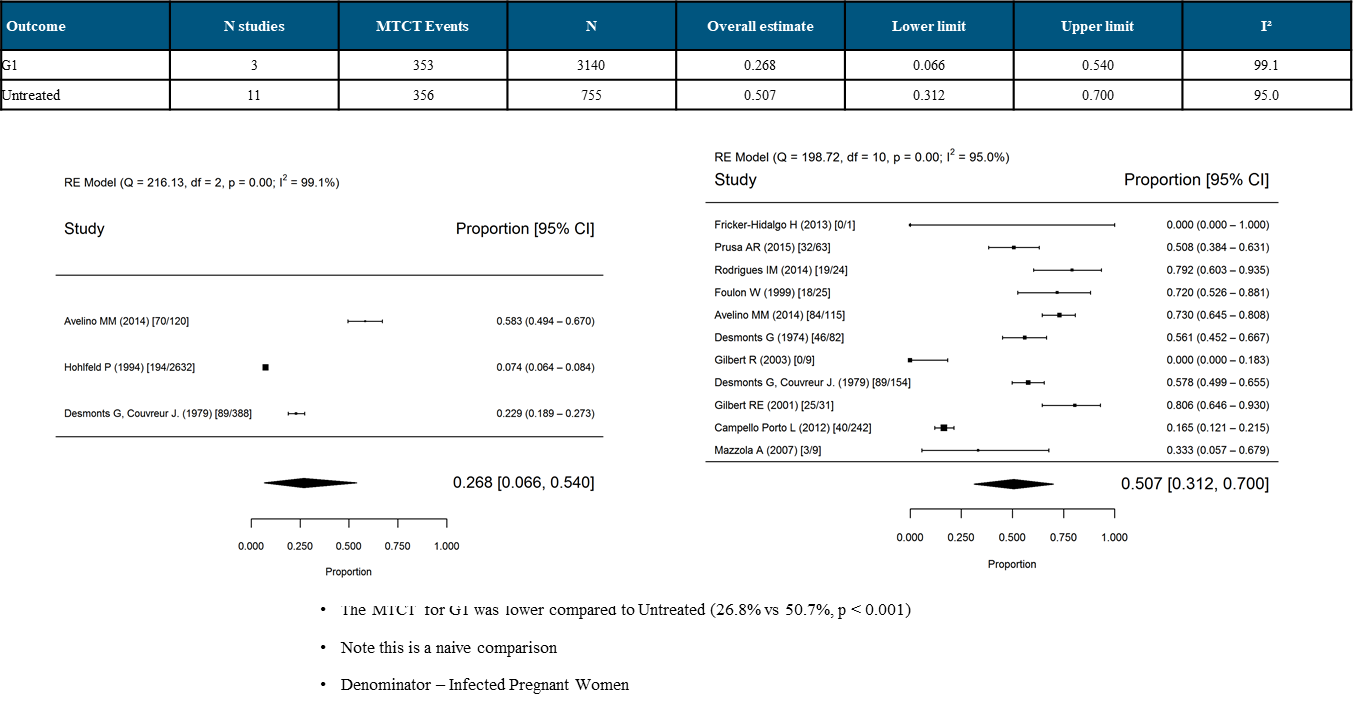


1. MTCT, G1+G2 vs Untreated, Sensitivity Analysis


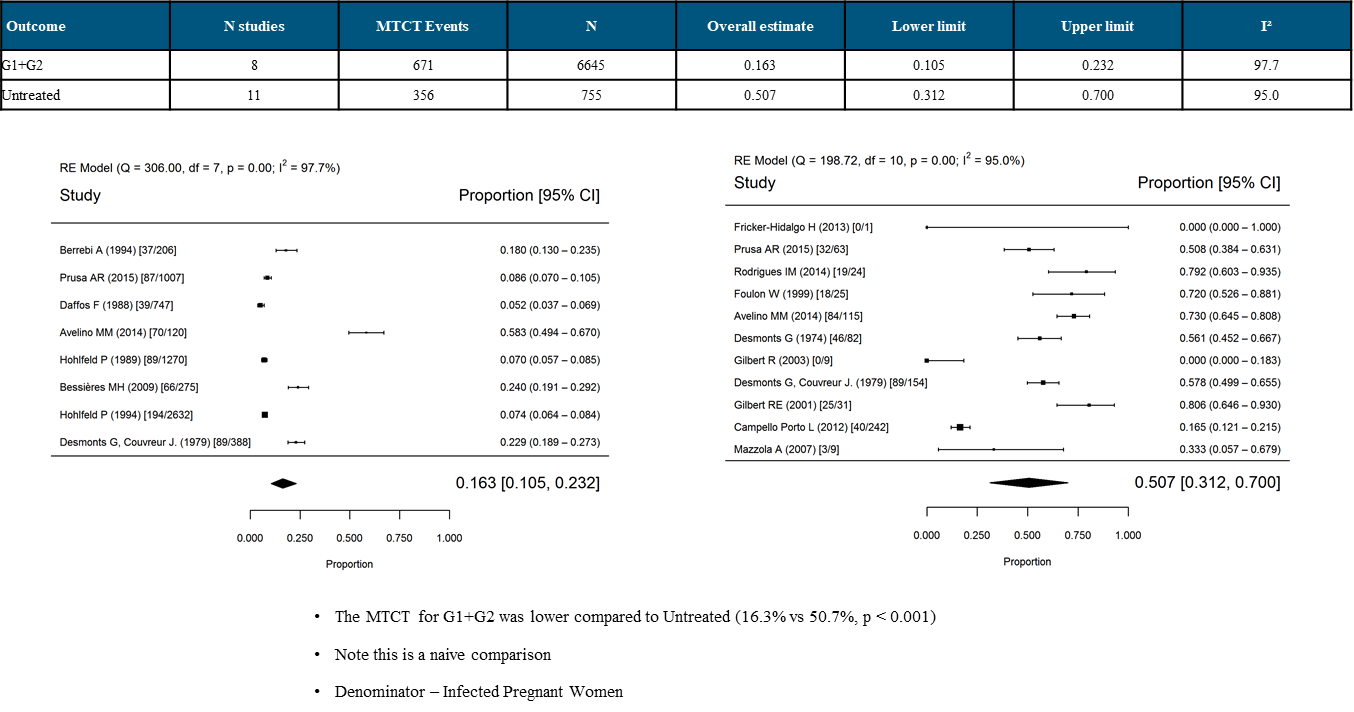


1. MTCT, G1 vs Untreated, 1^st^ Trimester, Sensitivity Analysis


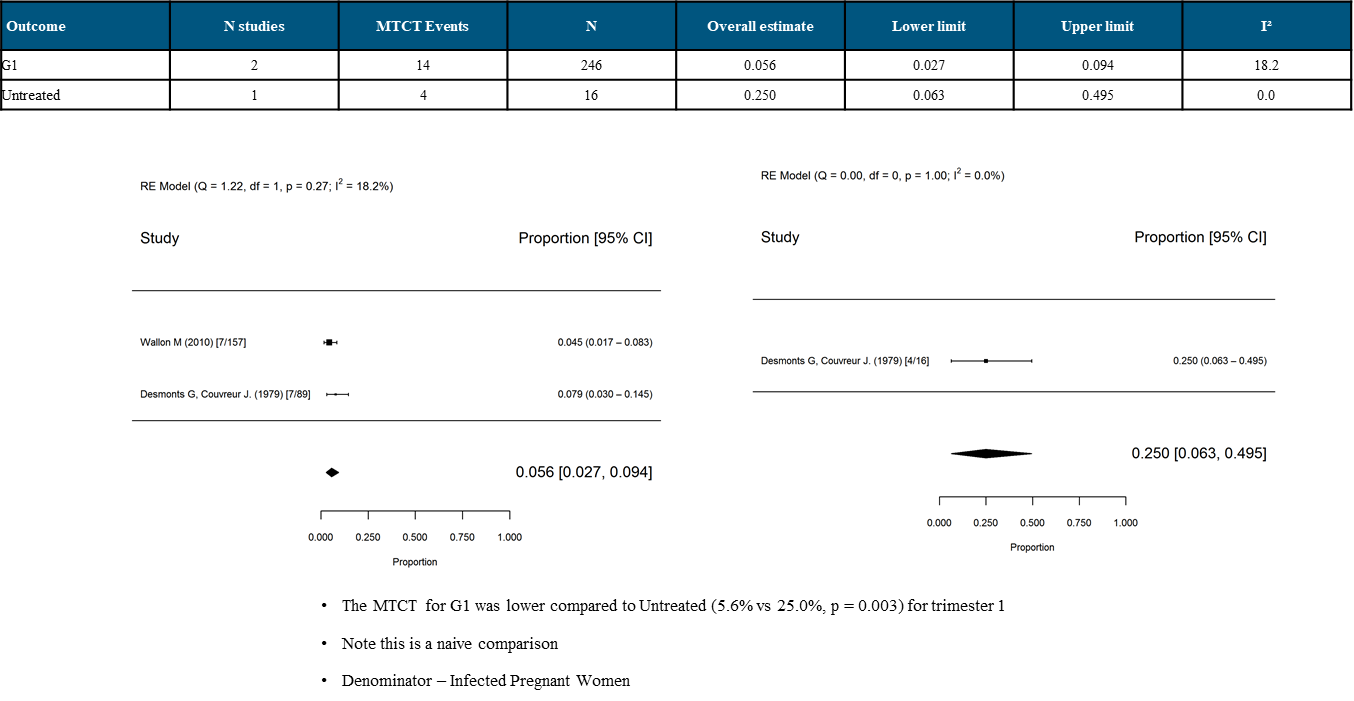


1. MTCT, G1+G2 vs Untreated, 1^st^ Trimester, Sensitivity Analysis


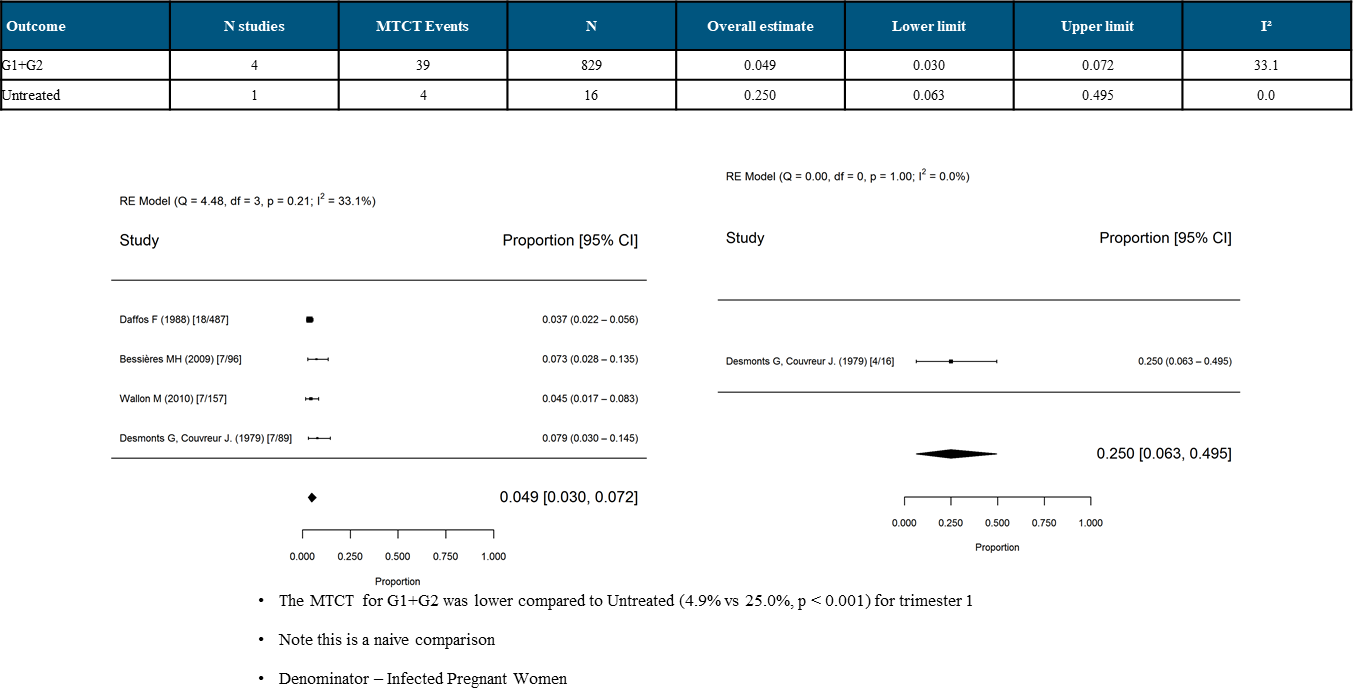


1. MTCT, G1 vs Untreated, 2^nd^ Trimester, Sensitivity Analysis


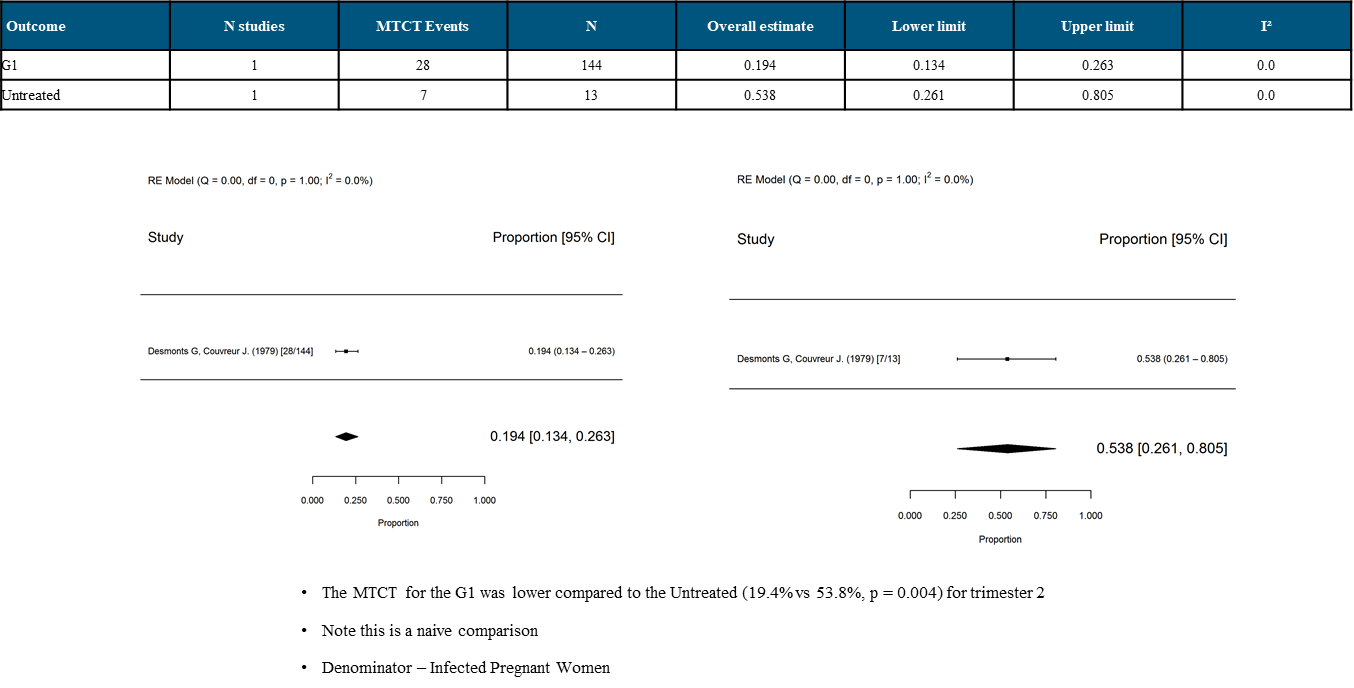


1. MTCT, G1+G2 vs Untreated, 2^nd^ Trimester, Sensitivity Analysis


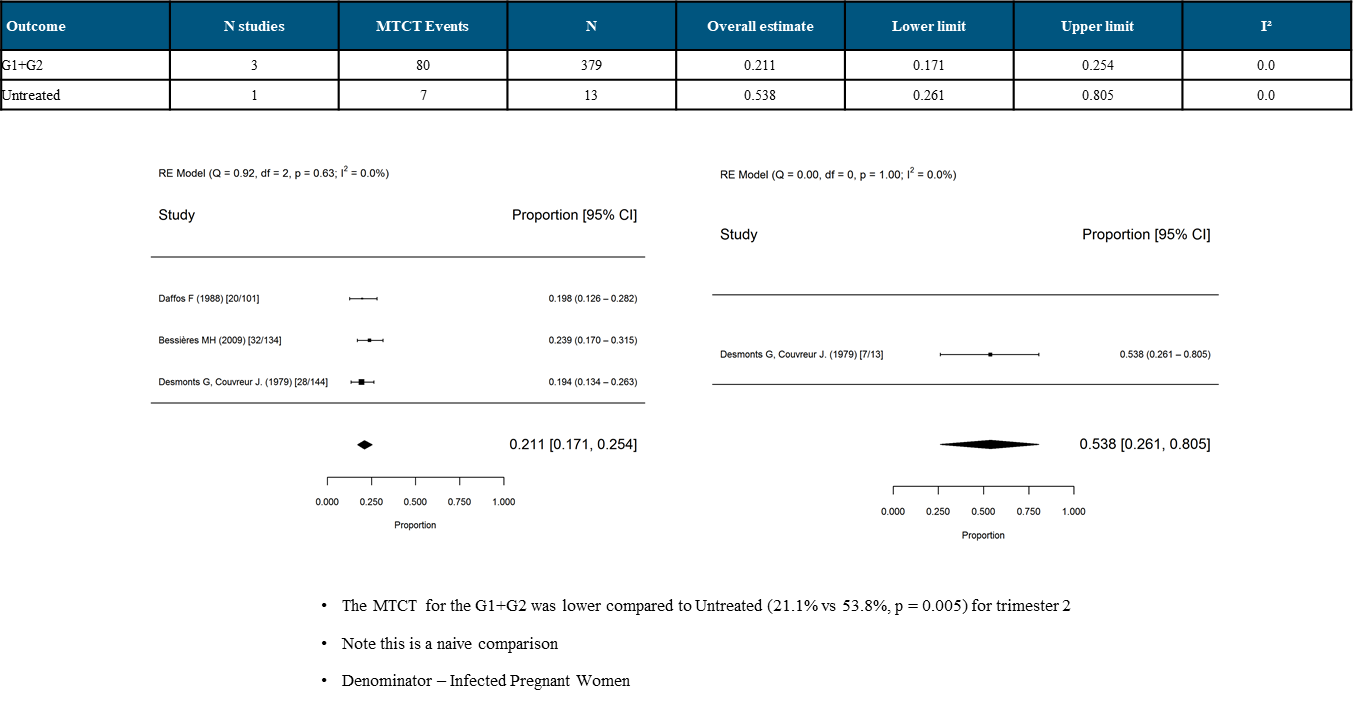


1. MTCT, G1 vs Untreated, 3^rd^ Trimester, Sensitivity Analysis


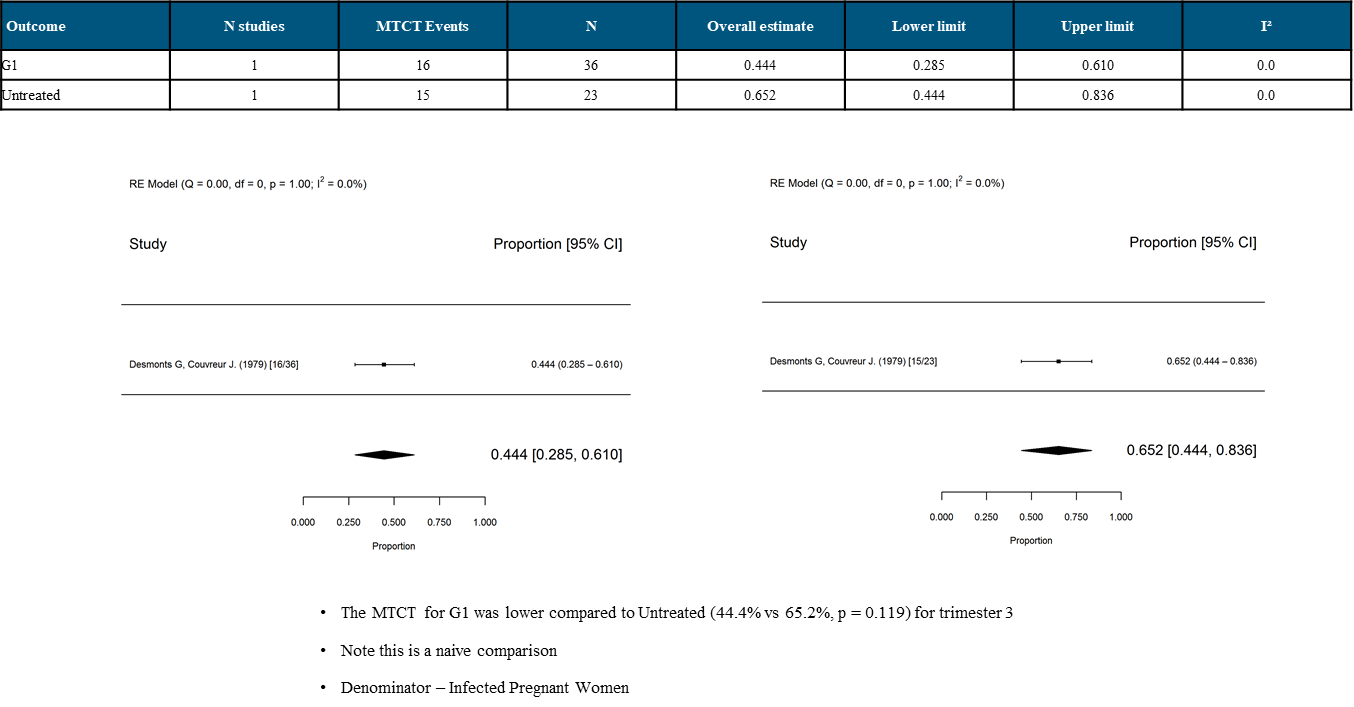


1. MTCT, G1+G2 vs Untreated, 3^rd^ Trimester, Sensitivity Analysis


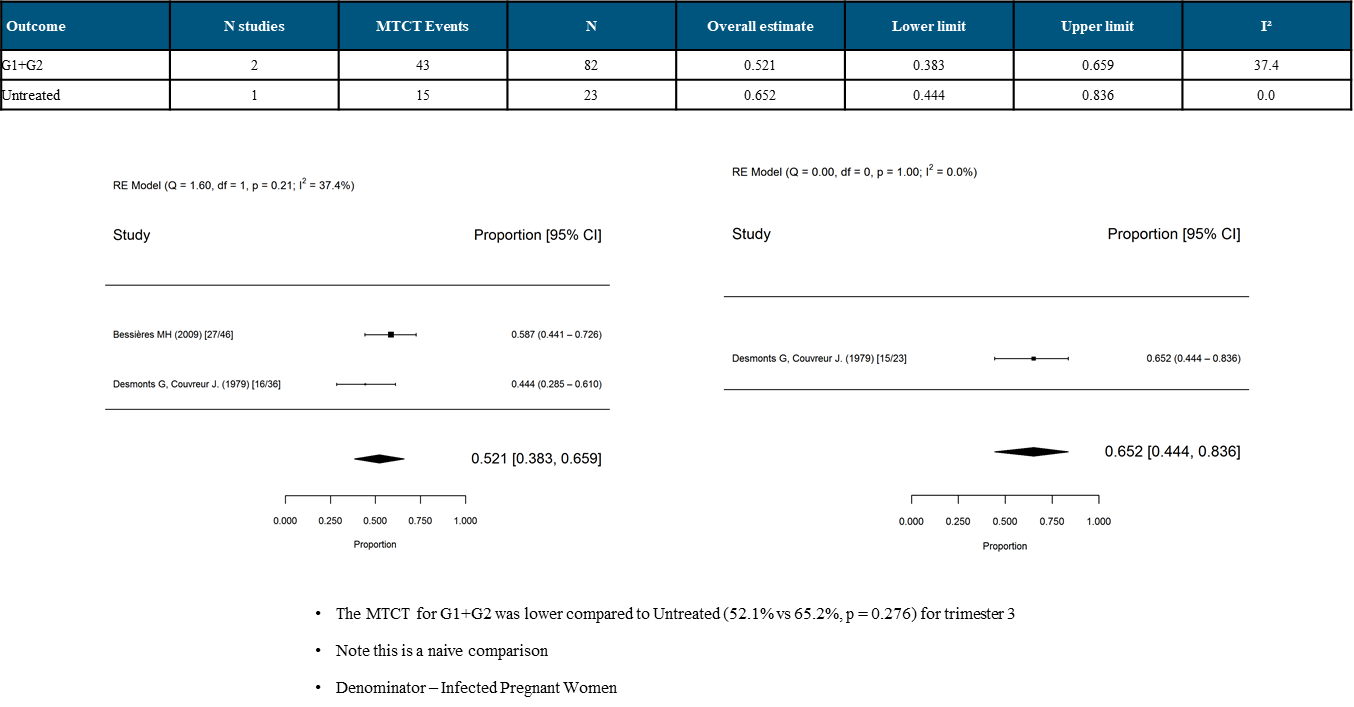


1. MTCT, G1 vs Untreated, 1^st^ and 2^nd^ Trimesters, Sensitivity Analysis


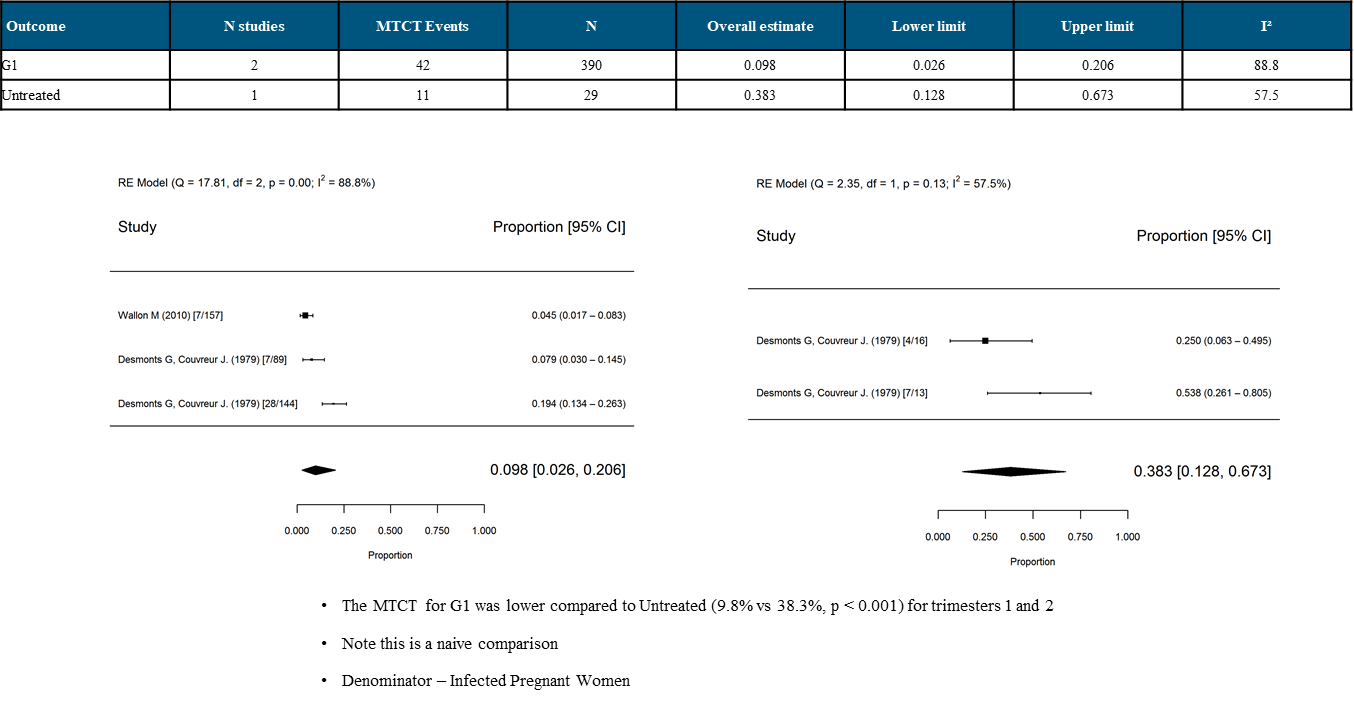


1. MTCT, G1+G2 vs Untreated, 1^st^ and 2^nd^ Trimesters, Sensitivity Analysis


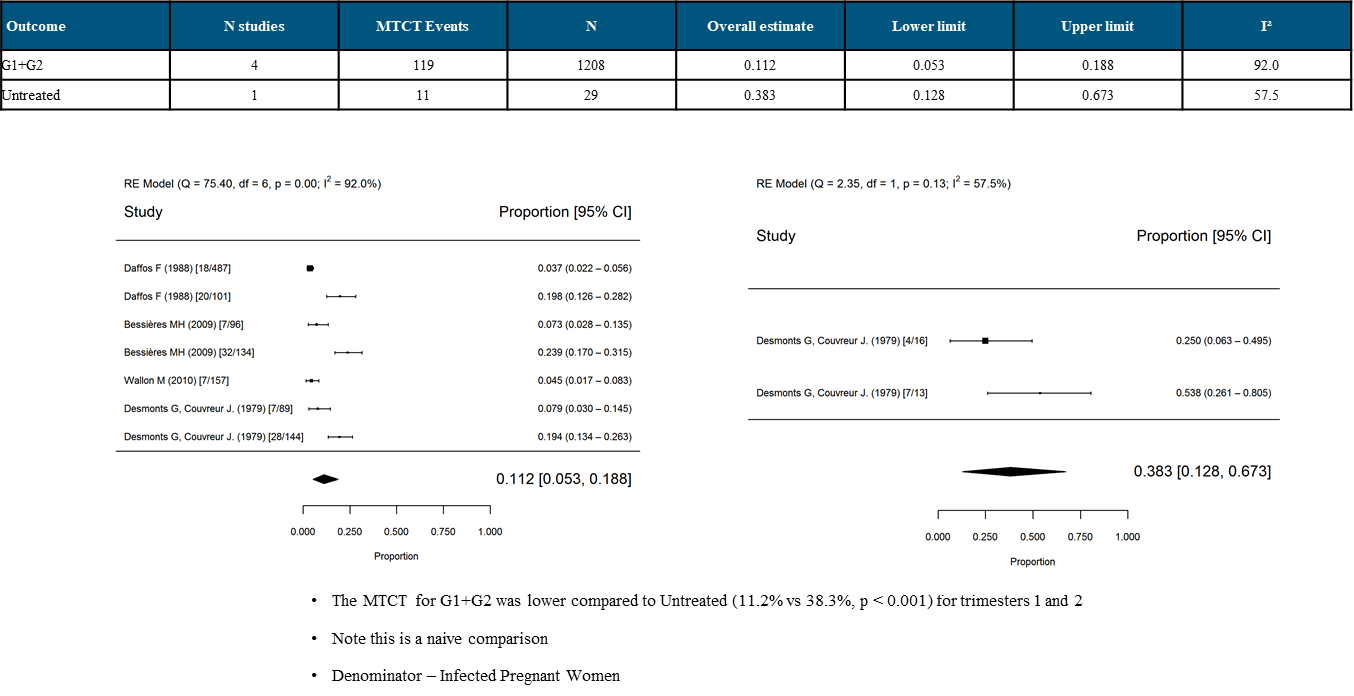


1. MTCT, G1 vs Untreated, Sensitivity Analysis, Excluding Avelino 2014


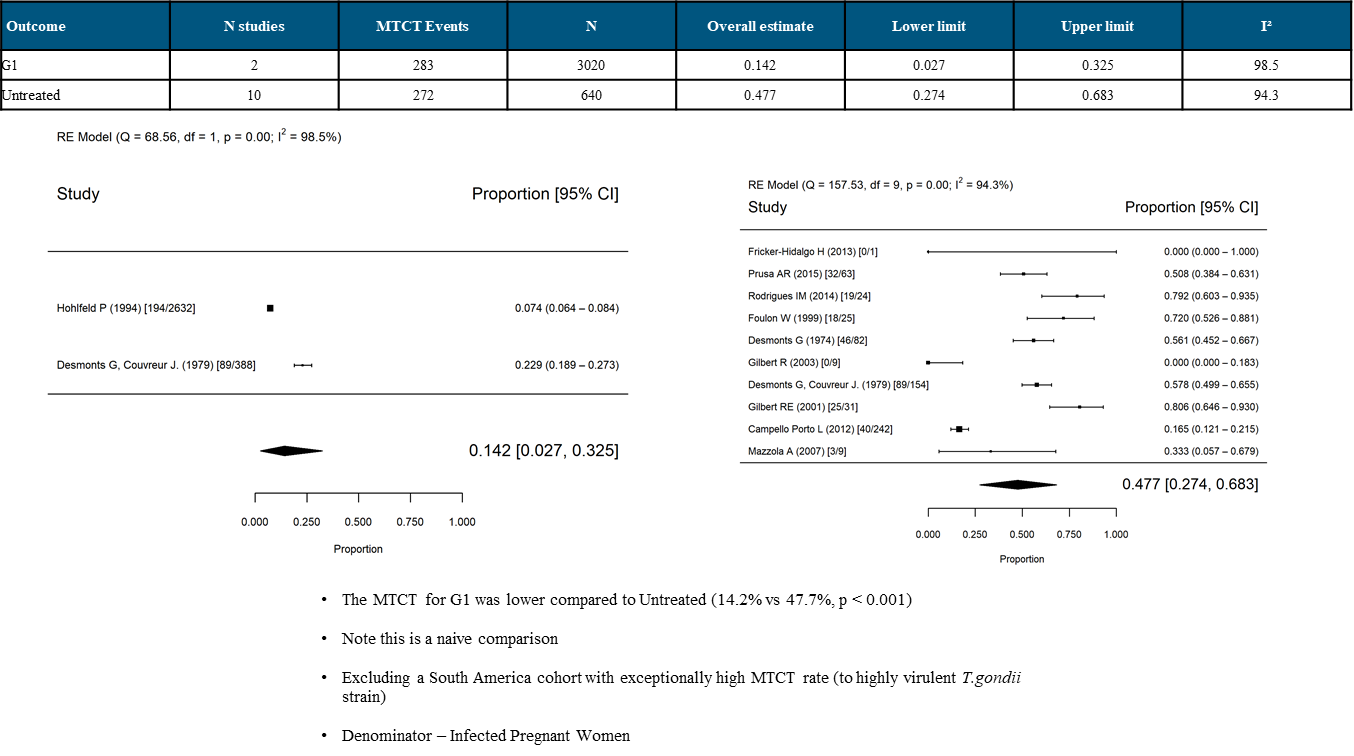


1. MTCT, G1+G2 vs Untreated, Sensitivity Analysis, Excluding Avelino 2014


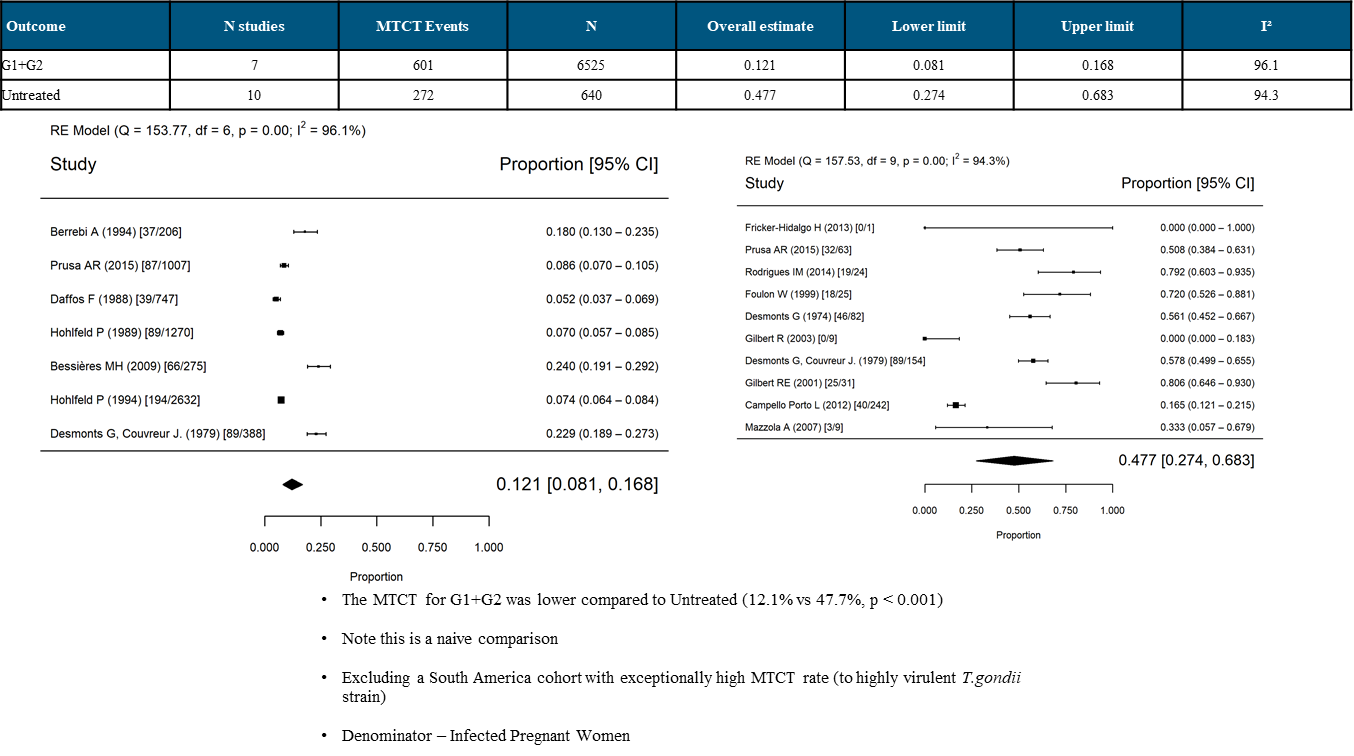


### Mortality due to CT Excluding *Elective* Terminations due to Fetal Infection - Sensitivity Analyses

1. Mortality, Excluding Elective Terminations, G1 vs Untreated, Up to 1 year, Sensitivity Analysis


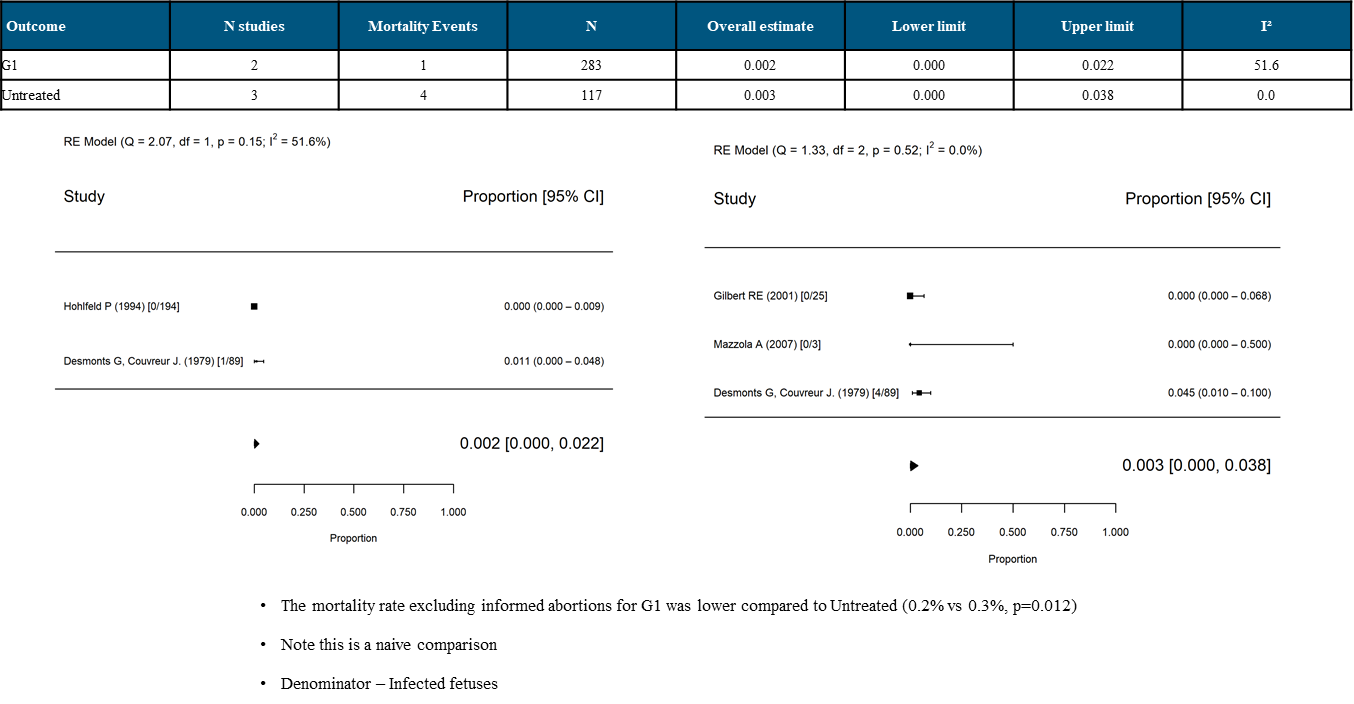


1. Mortality, Excluding Elective Terminations, G1+G2 vs Untreated, Up to 1 year, Sensitivity Analysis


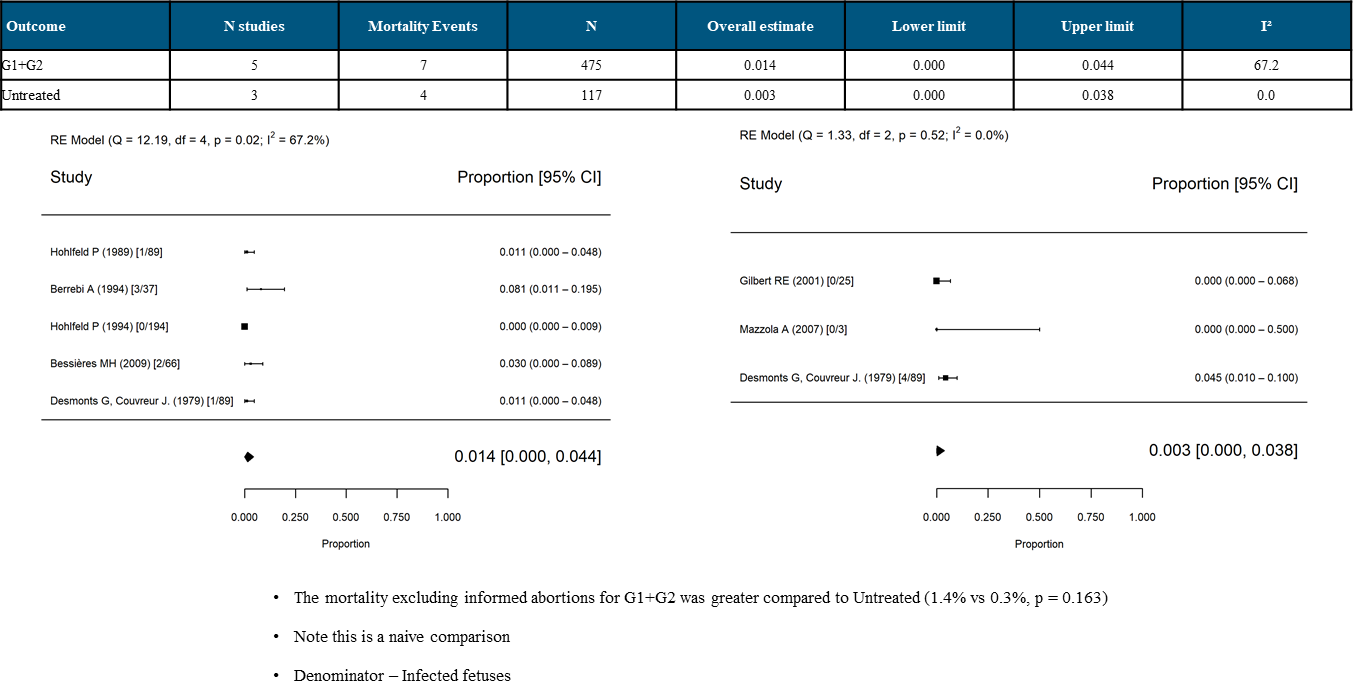


1. Mortality, Excluding Elective Terminations, G1+G2, Beyond 1 year, Sensitivity Analysis


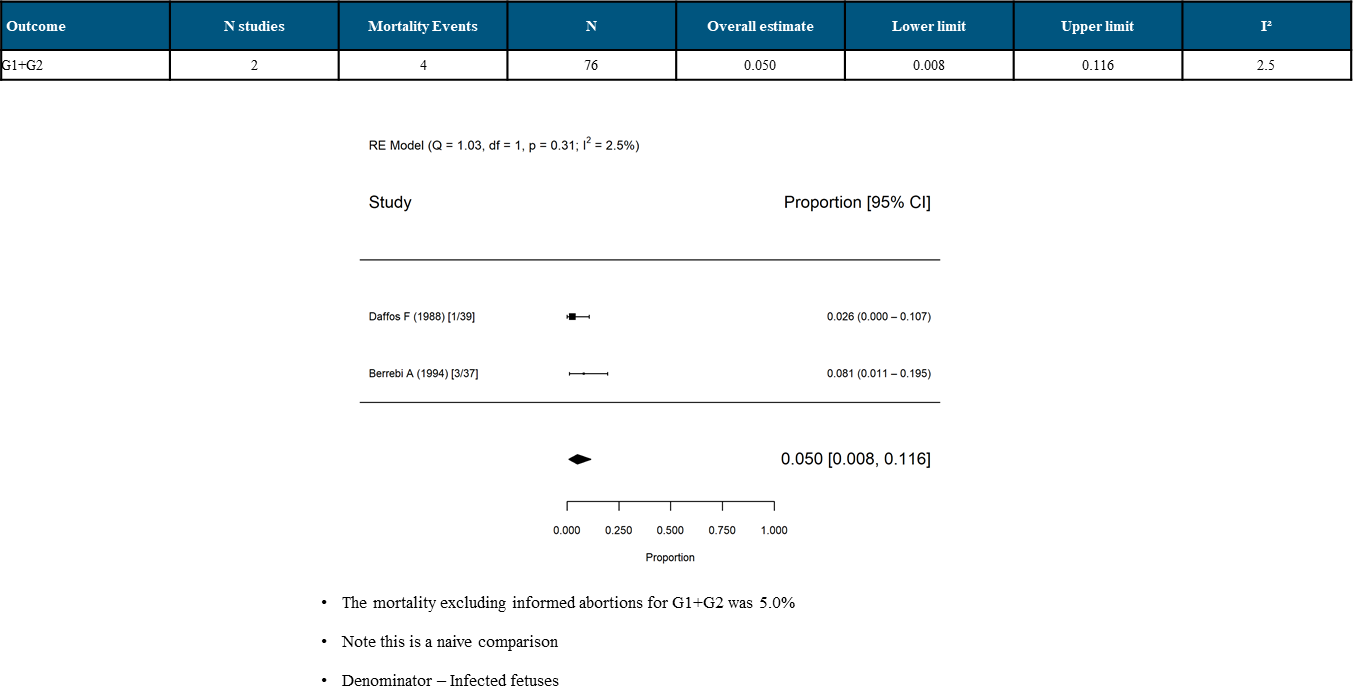


### Serious/Severe Sequelae and All Mortality: Infected Pregnant Women - Sensitivity Analyses

1. All Serious/Severe Sequelae and All Mortality, G1 vs Untreated, Up to 1 year, Sensitivity Analysis


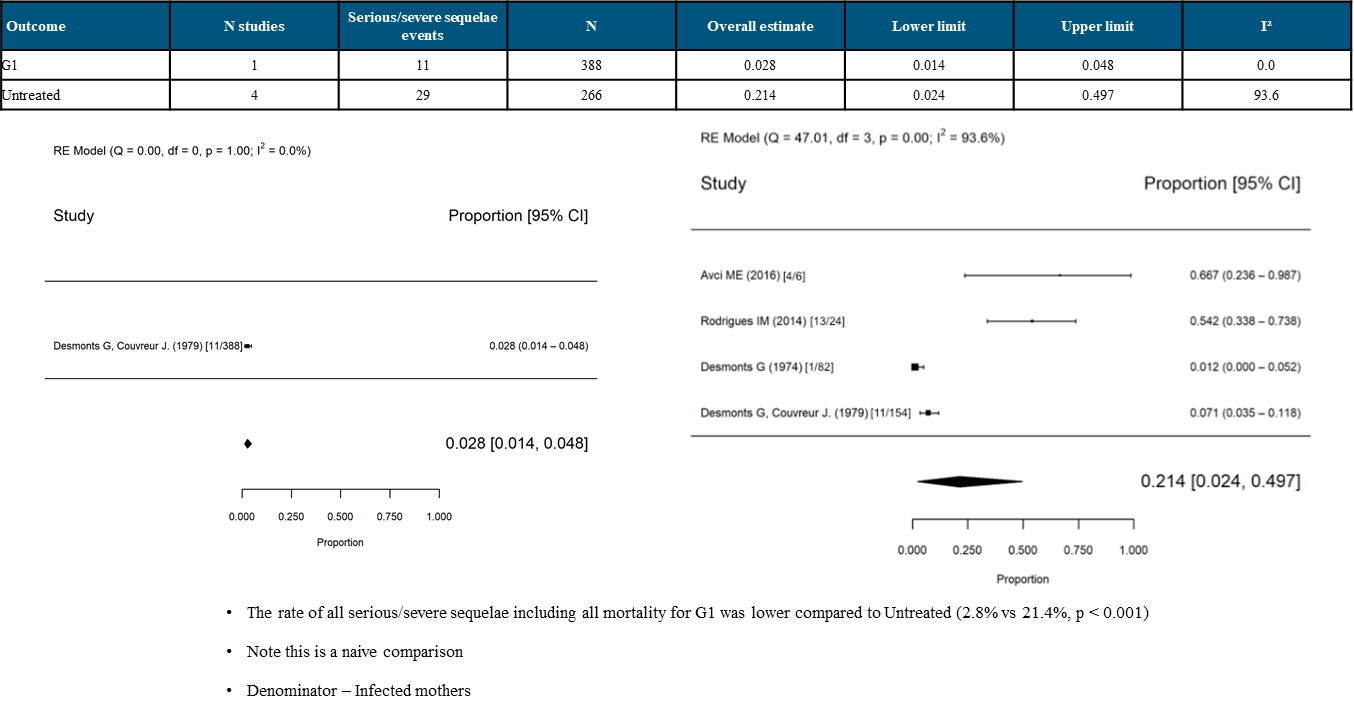


1. All Serious/Severe Sequelae and All Mortality, G1+G2 vs Untreated, Up to 1 year, Sensitivity Analysis


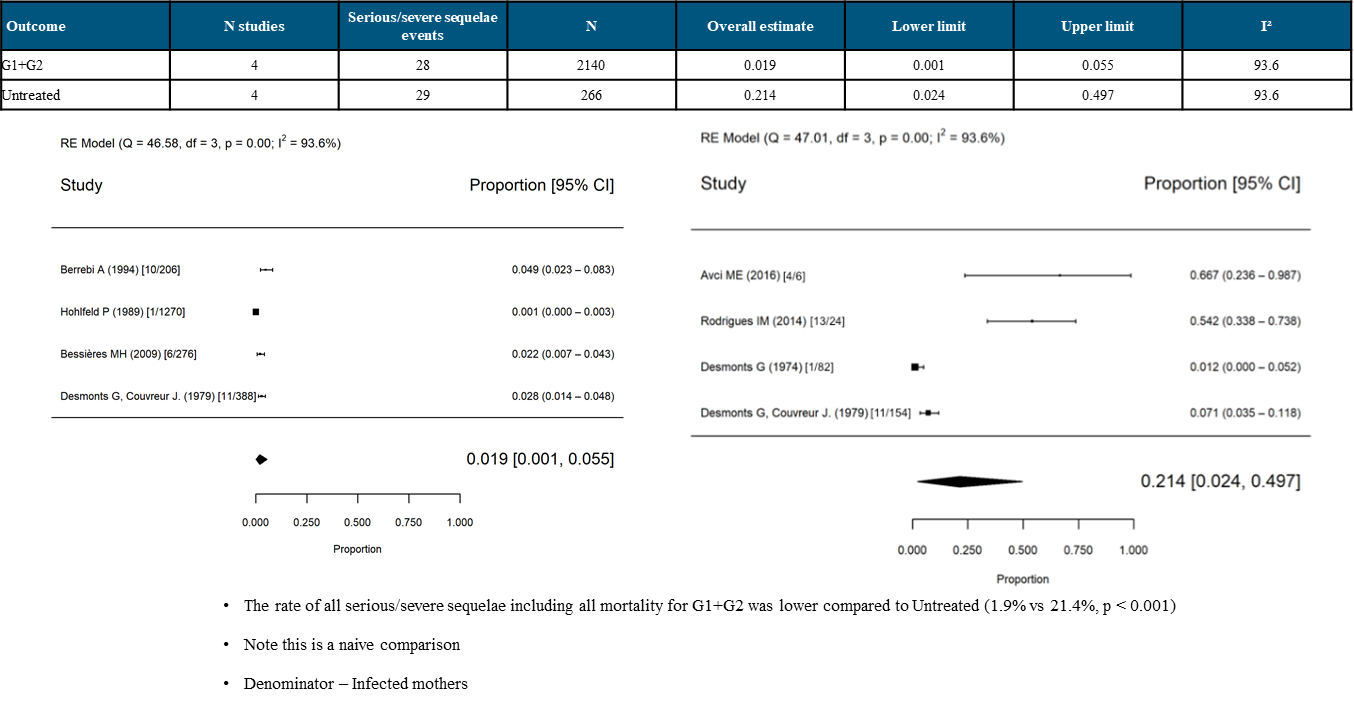


1. All Serious/Severe Sequelae and All Mortality, G1+G2, Beyond 1 year, Sensitivity Analysis


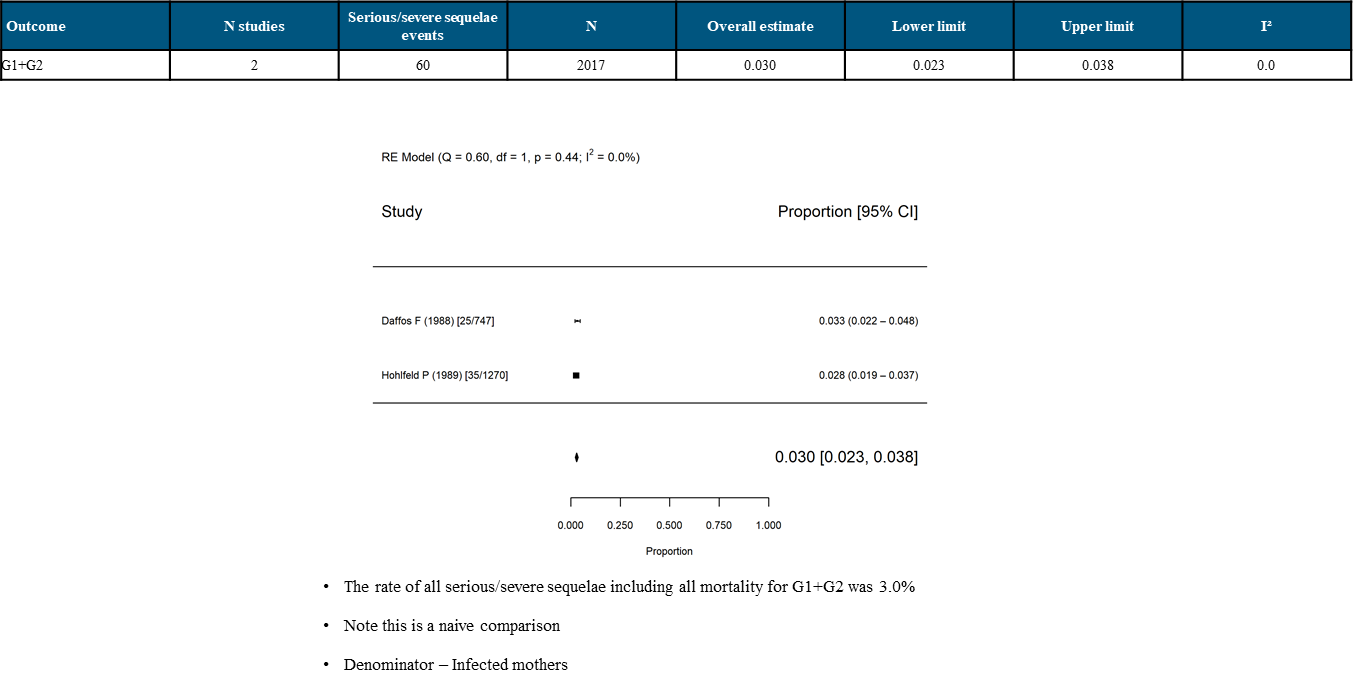


### Serious/Severe Sequelae and All Mortality: Infected Fetuses - Sensitivity Analyses

1. All Serious/Severe Sequelae and All Mortality, G1 vs Untreated, Up to 1 year, Sensitivity Analysis


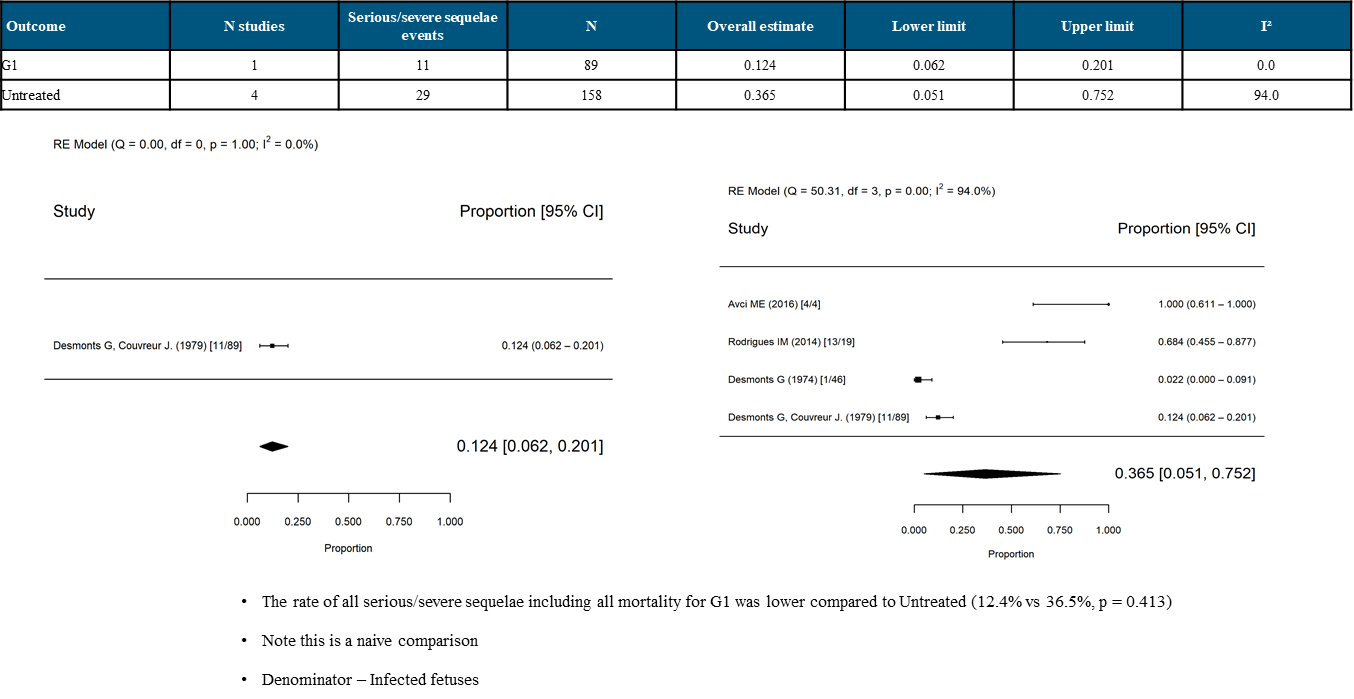


1. All Serious/Severe Sequelae and All Mortality, G1+G2 vs Untreated, Up to 1 year, Sensitivity Analysis


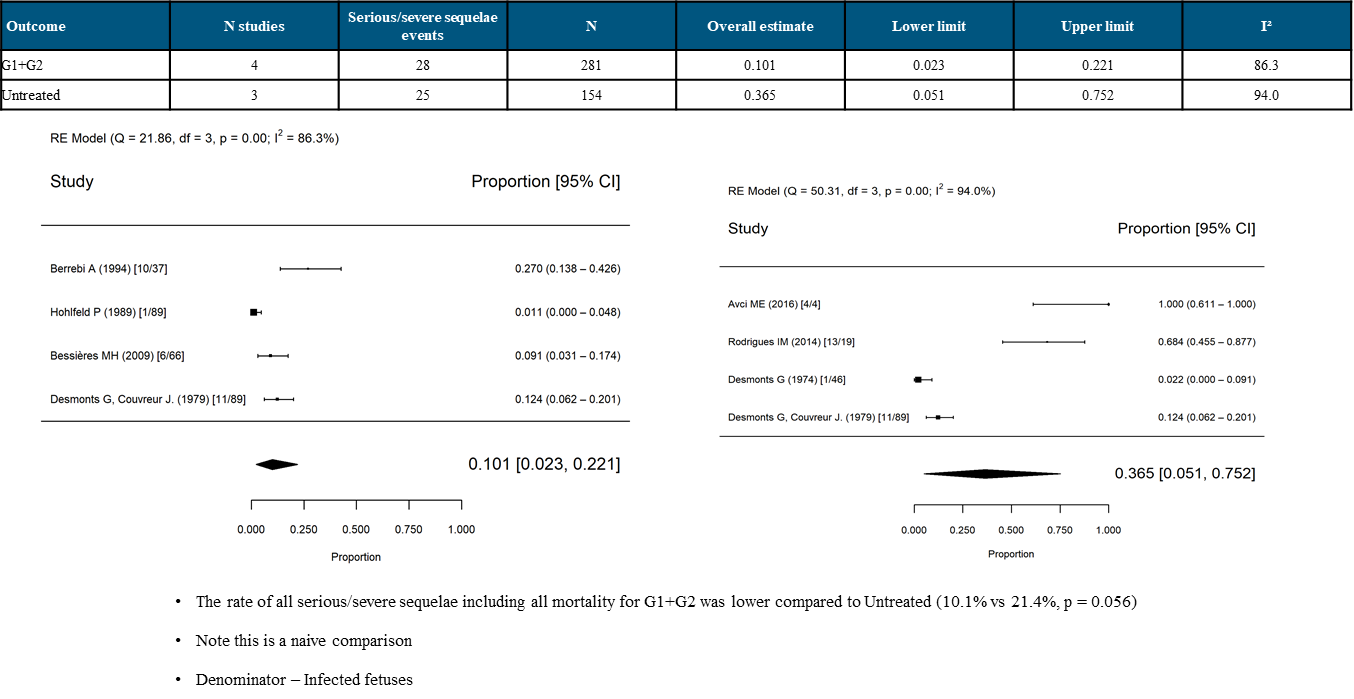


1. All Serious/Severe Sequelae and All Mortality, G1+G2, Beyond 1 year, Sensitivity Analysis


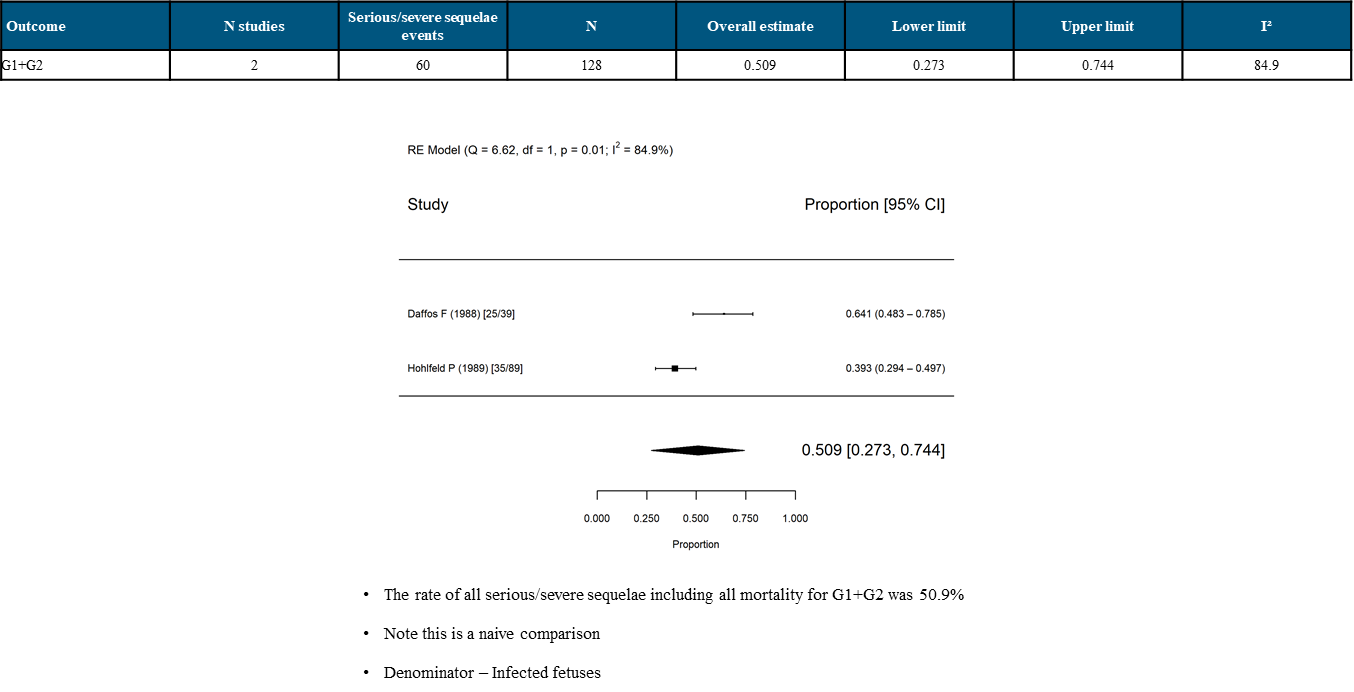


### Mild/Moderate/Severe Sequelae and Infant Mortality - Sensitivity Analyses

1. All Mild/Moderate/Severe Sequelae and Infant Mortality (Postnatal), G1 vs Untreated, Up to 1 year, Sensitivity Analysis


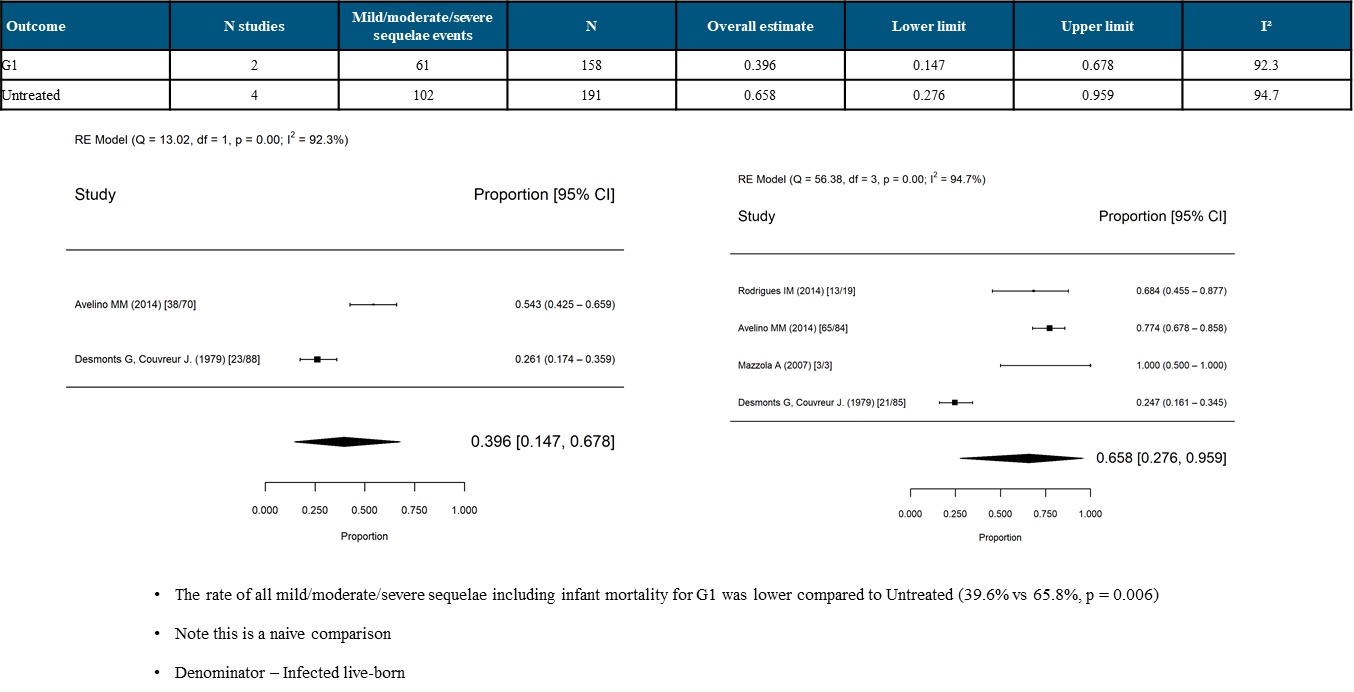


1. All Mild/Moderate/Severe Sequelae and Infant Mortality (Postnatal), G1+G2 vs Untreated, Up to 1 year, Sensitivity Analysis

1. All Mild/Moderate/Severe Sequelae and Infant Mortality (Postnatal), G1+G2, Beyond 1 year, Sensitivity Analysis

### Subclinical Disease - Sensitivity Analyses

1. Subclinical Disease, G1 vs Untreated, Up to 1 year, Sensitivity Analysis

1. Subclinical Disease, G1+G2 vs Untreated, Up to 1 year, Sensitivity Analysis

1. Subclinical Disease, G1+G2, Beyond 1 year, Sensitivity Analysis

### Chorioretinitis - Sensitivity Analyses

1. Chorioretinitis, G1+G2 vs Untreated, Up to 1 year, Sensitivity Analysis

1. Chorioretinitis, G1 vs Untreated, Beyond 1 year, Sensitivity Analysis

1. Chorioretinitis, G1+G2 vs Untreated, Beyond 1 year, Sensitivity Analysis

# Tables

1. Outcome Definition and Denominators

| Outcome | Definition | Denominators |
| --- | --- | --- |
| Mother-to-child transmission (MTCT) | Transmission of toxoplasma gondii from mother to child | All infected mothers |
| Mortality (pre- and postnatal) excluding elective terminations* | Includes toxoplasmosis-associated spontaneous abortions, stillbirths, and postnatal infant deaths | All infected fetuses/children |
| All serious/severe sequelae and all mortality (pre- and postnatal) | Includes serious/severe sequelae and all mortality (all toxoplasmosis-associated terminations, miscarriages, stillbirths, and infant deaths within the first year of life) | All infected mothers  All infected fetuses/children |
| All mild/moderate/severe sequelae and infant mortality (postnatal) | Includes postnatal mild/moderate/severe sequelae and toxoplasmosis associated infant mortality; excludes terminations and stillbirths | All infected live-born children |
| Subclinical disease | Newborns with confirmed infection based on serology and/or neonatal parasite examination, but with no other clinical signs/symptoms | All infected live-born children |
| Chorioretinitis | Chorioretinitis of the infected live-born child | All infected live-born children |

* Elective terminations are terminations of pregnancy that have been decided upon after suspected or confirmed diagnosis of toxoplasmosis

1. Qualifications for Inclusion and Denominator Calculations

| Serious/Severe Sequelae Inclusion |
| --- |
| If a child had any of the following findings, his or her sequelae was considered to be in the “severe” stratum:   1. ≥3 punctate focal calcifications or abnormal density of white matter in initial CT scan findings 2. seizures 3. motor abnormalities 4. hydrocephalus 5. microcephalus 6. cerebral palsy 7. unilateral or bilateral dilation of the ventricles 8. elevated CSF protein levels 9. low CSF glucose levels 10. CSF T. gondii – speciﬁc antibody production ratio >1 11. macular scarring that threatens vision 12. optic atrophy 13. severe visual impairment 14. microphthalmia 15. sensorineural hearing loss 16. stillbirth 17. death from any cause related to toxoplasmosis or its complications including termination of pregnancy, excluding accident, before the age of 1 year |
| Mild/Moderate Sequelae Inclusion |
| Mild/moderate sequelae was defined as any sequelae that are not serious/severe or subclinical. |
| Denominator Calculations |
| All infected mothers:  Rate of a specific outcome endpoint = (number of infected fetuses or children meeting a specific outcome criteria / number of the mothers infected with T. gondii during pregnancy) x 100%. |
| All infected fetuses/children:  Rate of a specific outcome endpoint = (number of infected fetuses or children meeting a specific outcome criteria / number of all infected fetuses or children) x 100% |
| Infected live-born children:  Rate of a specific outcome endpoint = (number of infected live-born children meeting a specific outcome criteria / number of all infected live-born children) x 100% |

1. Study and Patient Characteristics

| Author & Year | Study Design | Country | Treatment Groups | Study Population (Mothers, Offspring) | Age | Key Study |
| --- | --- | --- | --- | --- | --- | --- |
| Avci 2016[[19](#_ENREF_19)] | Cohort | NR | Spiramycin NR (G1)  Untreated NR (G3) | 61, 61 | 27.2 yr (± 6.1) | N; screening, spiramycin dose, and sample size |
| Avelino 2014[[20](#_ENREF_20)] | Cohort | Brazil | Spiramycin 3 g qd / Untreated Until Delivery (Newborn) (pooled)  Spiramycin 3 g qd Until Delivery (Newborn) (G1)  Untreated Until Delivery (G3) | 246, 246 | NR | Y |
| Berrebi 1994[[6](#_ENREF_6)] | Cohort | France | Spiramycin 3 g qd Until Delivery (+prn) (G1 + G2) | 206, 162 | NR | Y |
| Berrebi 2010[[21](#_ENREF_21)] | Cohort | France | Spiramycin 3 g qd Until Delivery (G1)  Spiramycin 3 g qd NR > Pyrimethamine 50 mg qw + Sulfadoxine 1000 mg qw + Folinic acid 50 mg qw Until Delivery (G2)  Spiramycin 3 g qd NR / (Spiramycin 3 g qd NR > Pyrimethamine 50 mg qw + Sulfadoxine 1000 mg qw + Folinic acid 50 mg qw) Until Delivery (G1 + G2) | 676, 681 | NR | Y |
| Bessières 2001[[10](#_ENREF_10)] | Cohort | France | Spiramycin 3 g qd NR >(+) Pyrimethamine 50 mg biw + Sulfadoxine 1000 mg biw + Folic Acid NR >(-) Pyrimethamine 50 mg biw + Sulfadoxine 1000 mg biw Until Delivery (G1 + G2) | 165, 165 | NR | N; published between 1999-2006 |
| Bessières 2009[[7](#_ENREF_7)] | Cohort | France | Spiramycin NR > Spiramycin / (Pyrimethamine + Sulfadoxine) Until Delivery (G1 + G2) | 325, 276 | NR | Y |
| Brezin 2003[[22](#_ENREF_22)] | Cohort | France | Spiramycin NR > (Pyrimethamine 50 mg qd + Sulfadiazine 3 g qd + Folinic Acid 4wk > Spiramycin 3 g qd 6wk) cyc Until Delivery (G2) | NR, 18 | 4.5 yr (Total: 0.83 – 11.5) | N; published between 1999-2006 |
| Campello Porto 2012[[17](#_ENREF_17)] | Cohort | Brazil | Spiramycin bid 21d (G1)  (Spiramycin bid q21d > Pyrimethamine qd + Sulfadiazine qid+ Folinic Acid tiw q21d) cyc Until Delivery (G2)  Untreated Until Delivery (G3)  Pyrimethamine qd + Sulfadiazine qid + Folinic Acid tiw 21d  Spiramycin bid 21d / (Pyrimethamine qd + Sulfadiazine qid + Folinic Acid tiw Until Delivery) / (Spiramycin bid q21d > Pyrimethamine qd + Sulfadiazine qid + Folinic Acid tiw q21d) cyc Until Delivery (pooled) | 519, 524 | NR | N; screening, spiramycin dose |
| Cortina-Borja 2010[[23](#_ENREF_23)] | Cohort | France, Austria, Italy, Sweden, Poland, Denmark | Spiramycin NR (G1)  Untreated NR (G3)  Untreated NR (pooled) (G3)  Pyrimethamine + Sulphonamide / Untreated NR  Spiramycin / Untreated NR | NR, 293 | NR | Y |
| Daffos 1988[[8](#_ENREF_8)] | Cohort | France | Spiramycin 3 g qd > Spiramycin / (Pyrimethamine + Sulfonamides + Folinic Acid) Until Delivery (+prn) (G1 + G2) | 746, 747 | NR | Y |
| Desmonts 1974[[11](#_ENREF_11)] | Cohort | France | Spiramycin 2-3 g qd cyc Until Delivery (G1)  Untreated Until Delivery (G3)  Spiramycin 2-3 g qd cyc / Untreated Until Delivery | 378, 378 | NR | N; screening and spiramycin dose |
| Desmonts 1979[[15](#_ENREF_15)] | Cohort | France | Spiramycin NR (G1)  Untreated NR (G3)  Spiramycin / Untreated NR (pooled) | 542, 542 | NR | Y |
| Faucher 2012[[24](#_ENREF_24)] | Cohort | France | Spiramycin 3 g qd Until Delivery (G1)  Spiramycin 3 g qd NR> Pyrimethamine 1 g q10d + Sulfadoxine 50 mg q10d + Folinic Acid 50 mg q10d Until Delivery (G2)  Spiramycin 3 g qd / Spiramycin 3 g qd NR> Pyrimethamine 1 g q10d + Sulfadoxine 50 mg q10d + Folinic Acid 50 mg q10d Until Delivery (pooled) (G1 + G2)  Untreated Until Delivery (G3)  Pyrimethamine 1 g q10d + Sulfadoxine 50 mg q10d + Folinic Acid 50 mg q10d Until Delivery | 127, 127 | NR | Y |
| Foulon 1999[[2](#_ENREF_2)] | Cohort | Belgium, Finland, France, Norway | Spiramycin 1 g / 3 g qd > Spiramycin / (Pyrimethamine 25 mg qd + Sulfadiazine 3 g qd + Folinic Acid bid 3wk > Spiramycin 3 g qd 3wk) cyc / (Pyrimethamine 25 mg q2w + Sulfadoxine 500 mg q2w + Folinic Acid 50 mg q2w) Until Delivery (G1 + G2)  Untreated Until Delivery (G3)  (Spiramycin 1 g / 3 g qd > Spiramycin / (Pyrimethamine 25 mg qd + Sulfadiazine 3 g qd + Folinic Acid bid q3w > Spiramycin 3 g qd q3w) cyc / (Pyrimethamine 25 mg q2w + Sulfadoxine 500 mg q2w + Folinic Acid 50 mg q2w)) / (Pyrimethamine 25 mg qd + Sulfadiazine 3 g qd + Folinic Acid 5 mg biw q3w > Spiramycin 3 g qd q3w) cyc / Azithromycin 500 mg qd Until Delivery (pooled)  (Pyrimethamine 25 mg qd + Sulfadiazine 3 g qd + Folinic Acid 5 mg biw 3wk > Spiramycin 3 g qd q6w) cyc Until Delivery | 144, 144 | NR | N; screening and published between 1999-2006 |
| Fricker-Hidalgo 2013[[4](#_ENREF_4)] | Cohort | France | Spiramycin 3 g qd Until Delivery (G1)  Untreated Until Delivery (G3) | 26, NR | NR | N; sample size |
| Galanakis 2007[[25](#_ENREF_25)] | Cohort | Greece | Spiramycin 3 g qd Until Delivery / (Spiramycin 3 g qd > Pyrimethamine + Sulfadiazine) / (Spiramycin 3 g qd > Roxithromycin) | 35, 35 | NR | N; screening, transmission rate was not evaluated, and sample size |
| Gilbert 2001[[26](#_ENREF_26)] | Cohort | France | Untreated Until Delivery (G3)  Spiramycin 3 g qd / (Pyrimethamine 50 mg qd + Sulfadiazine 3 g qd q3w > Spiramycin q3w) cyc / (Spiramycin 3 g qd > (Pyrimethamine 50 mg qd + Sulfadiazine 3 g qd q3w > Spiramycin qd q3w) cyc) Until Delivery (pooled) | 554, 554 | NR | N; published between 1999-2006 |
| Gilbert 2003[[3](#_ENREF_3)] | Cohort | Austria, France, Italy, Sweden | Spiramycin 3 g qd Until Delivery (G1)  Spiramycin 3 g qd NR > Pyrimethamine 25-50 mg + Sulphonamide 0.75-3 g qd Until Delivery (G2)  Spiramycin / ((Spiramycin > Pyrimethamine + Sulphonamide + Folinic Acid) +/- cyc) Until Delivery (Pooled) (G1 + G2)  Untreated Until Delivery (G3)  Pyrimethamine 25 mg qd (50 mg induction) + Sulphonamide 0.75 g qd (1.5 g induction) Until Delivery  Spiramycin / ((Spiramycin > Pyrimethamine + Sulphonamide + Folinic Acid) +/- cyc) / (Pyrimethamine 25 mg qd (50 mg induction) + Sulphonamide 0.75 g qd (1.5 g induction)) Until Delivery (Pooled) | 1208, 1208 | 28.4 yr (± 4.9) | N; published between 1999-2006 |
| Gras 2001[[27](#_ENREF_27)] | Cohort | France | Spiramycin 3 g qd Until Delivery (G1)  (Pyrimethamine 50 mg qd + Sulfadiazine 3 g qd 3wk > Spiramycin 3 g qd 6wk) cyc Until Delivery  Untreated Until Delivery (G3) | 181, 181 | NR | N; published between 1999-2006 |
| Gras 2005[[28](#_ENREF_28)] | Cohort | France, Austria, Italy, Sweden, Poland, Denmark | Untreated Until Delivery (G3)  Spiramycin / (Spiramycin > Pyrimethamine + Sulphonamide) / (Pyrimethamine + Sulphonamide) Until Delivery | 255, 255 | NR | N; published between 1999-2006 |
| Gratzl 1998[[5](#_ENREF_5)] | Cohort | Austria | (Untreated / Spiramycin 3 g qd NR) > Spiramycin 3 g qd Until Delivery (G1)  Spiramycin 3 g qd 16wk > (Sulfadiazine 3 g qd + Pyrimethamine 25 mg qd + Folinic Acid 5 mg biw q4w > Spiramycin 3 g qd q4w) cyc Until Delivery (G2)  Untreated NR > Spiramycin 3 g qd 16wk > (Sulfadiazine 3 g qd + Pyrimethamine 25 mg qd + Folinic Acid 5 mg biw q4w > Spiramycin 3 g qd q4w) cyc Until Delivery (G2)  [Spiramycin 3 g qd 16wk > (Sulfadiazine 3 g qd + Pyrimethamine 25 mg qd + Folinic Acid 5 mg biw q4w > Spiramycin 3 g qd q4w) cyc Until Delivery] / [(Untreated / Spiramycin 3 g qd NR) > Spiramycin 3 g qd Until Delivery] (pooled) (G1 + G2) | 48, 49 | NR | N; screening and sample size |
| Hohlfeld 1989[[12](#_ENREF_12)] | Cohort | France | Spiramycin 3 g qd NR > Spiramycin / (Pyrimethamine 50 mg + Sulfadiazine 3 g qd 3wk > Spiramycin qd 3wk) cyc / (Sulfadoxine 1500 mg + Pyrimethamine 75 mg q10d) Until Delivery (G1 + G2) | 1270, 1270 | NR | Y |
| Hohlfeld 1994[[13](#_ENREF_13)] | Cohort | France | Spiramycin 3 g qd Until Delivery (G1) | 2632, 2632 | NR | Y |
| Hotop 2012[[29](#_ENREF_29)] | Cohort | Germany | Spiramycin 3 g qd 16wk > Pyrimethamine 25-50 mg qd + Sulfadiazine 3-4 g qd + Folinic Acid 10-15 mg qw Until Delivery (G2)  (Spiramycin 3 g qd 16wk > Pyrimethamine 25-50 mg qd + Sulfadiazine 3-4 g qd + Folinic Acid 10-15 mg qw Until Delivery) / (Pyrimethamine 25-50 mg qd + Sulfadiazine 3-4 g qd + Folinic Acid 10-15 mg qw Until Delivery)  Pyrimethamine 25-50 mg qd + Sulfadiazine 3-4 g qd + Folinic Acid 10-15 mg qw Until Delivery | 685, 685 | NR | Y |
| Kieffer 2008[[30](#_ENREF_30)] | Cohort | France | (Spiramycin 3 g qd NR > Spiramycin 3 g qd / (Pyrimethamine 50 mg qd + Sulfadiazine 3 g qd) / (Pyrimethamine 50 mg qd q10d + Sulfadoxine 1 g qd q10d)) / Untreated Until Delivery | 300, 300 | NR | Y |
| Mazzola 2007[[18](#_ENREF_18)] | Cohort | NR | Spiramycin Until Delivery (G1)  Untreated Until Delivery (G3)  (Spiramycin NR > Pyrimethamine + Sulphadiazine NR) cyc Until Delivery | 58, 59 | NR | N; screening, spiramycin dose, and sample size |
| Prusa 2015[[1](#_ENREF_1)] | Cohort | Austria | Spiramycin 2.3 g 16wk > Spiramycin Until Delivery / (Pyrimethamine 25-50 mg qd + Sulfadiazine 0.75-1.5 g qd + Folinic Acid 15 mg tiw 4wk > Spiramycin 4wk) cyc Until Delivery (G1 + G2)  Untreated Until Delivery (G3)  Other Treatment Regimens Until Delivery | 1173, 1189 | NR | Y |
| Rodrigues 2014[[9](#_ENREF_9)] | Cross-Sectional | Brazil | Spiramycin 3 g qd Until Delivery (G1)  Untreated Until Delivery (G3) | 68, 68 | NR | N; screening and sample size |
| Valentini 2009[[31](#_ENREF_31)] | Cohort | Italy | Spiramycin 870 mg qid + (Cotrimoxazole 960 mg bid + Folinic Acid 4 mg qd wk14 to 2 weeks before delivery) Until Delivery | 76, 76 | NR | N; screening, spiramycin dose |
| Valentini 2015[[16](#_ENREF_16)] | Cohort | Italy | Spiramycin 870 mg qid Until Delivery (G1)  Spiramycin 16 weeks Gestation > Pyrimethamine 25 mg qd + Sulfadiazine 0.75 g qd Until Delivery (G2)  Spiramycin 870 mg qid Until Delivery + (Cotrimoxazole 960 mg bid + Folinic Acid 4 mg qd 14 week Gestation to 1 week Before Delivery) | 123, 123 | NR | N; screening and spiramycin dose |
| Vergani 1998[[14](#_ENREF_14)] | Cohort | Italy | Spiramycin 3 g qd Until Delivery (G1) | 154, 154 | NR | N; screening |
| Wallon 2010[[32](#_ENREF_32)] | Cohort | France | Spiramycin Median 6.7 wk (G1)  Spiramycin / (Spiramycin > Pyrimethamine + Sulfonamide) / (Primethamine + Sulfonamides) / Untreated Median 2.6 wk | 377, NR | NR | Y |
| Wallon 2013[[33](#_ENREF_33)] | Cohort | France | Untreated / Spiramycin / Pyrimethamine + Sulfadiazine NR (1987-1991)  Untreated / Spiramycin / Pyrimethamine + Sulfadiazine NR (1992-2008) | 2048, 2048 | 29 yr (± 5, Total: 16 – 48) | Y |
| NR=not reported, Y=yes, N=no | | | | | |  |

1. Risk of Bias Results

| Study | Study Design | Selection | Comparability | Outcome | Final Score |
| --- | --- | --- | --- | --- | --- |
| Avci 2016[[19](#_ENREF_19)] | Cohort | **** | -- | ** | 6 |
| Avelino 2014[[20](#_ENREF_20)] | Cohort | **** | -- | ** | 6 |
| Berrebi 1994[[6](#_ENREF_6)] | Cohort | ** | -- | * | 3 |
| Berrebi 2010[[21](#_ENREF_21)] | Cohort | **** | -- | *** | 7 |
| Bessières 2001[[10](#_ENREF_10)] | Cohort | ** | -- | *** | 5 |
| Bessières 2009[[7](#_ENREF_7)] | Cohort | *** | -- | ** | 5 |
| Brezin 2003[[22](#_ENREF_22)] | Cohort | *** | -- | ** | 5 |
| Campello Porto 2012[[17](#_ENREF_17)] | Cohort | *** | ** | ** | 7 |
| Cortina-Borja 2010[[23](#_ENREF_23)] | Cohort | **** | * | ** | 7 |
| Daffos 1988[[8](#_ENREF_8)] | Cohort | *** | * | *** | 7 |
| Desmonts 1974[[11](#_ENREF_11)] | Cohort | * | * | -- | 2 |
| Desmonts 1979[[15](#_ENREF_15)] | Cohort | * | * | -- | 2 |
| Faucher 2012[[24](#_ENREF_24)] | Cohort | ** | ** | * | 5 |
| Foulon 1999[[2](#_ENREF_2)] | Cohort | **** | ** | *** | 9 |
| Fricker-Hidalgo 2013[[4](#_ENREF_4)] | Cohort | *** | -- | *** | 6 |
| Galanakis 2007[[25](#_ENREF_25)] | Cohort | ** | -- | ** | 4 |
| Gilbert 2001[[26](#_ENREF_26)] | Cohort | *** | ** | ** | 7 |
| Gilbert 2003[[3](#_ENREF_3)] | Cohort | *** | ** | *** | 8 |
| Gras 2001[[27](#_ENREF_27)] | Cohort | **** | ** | * | 7 |
| Gras 2005[[28](#_ENREF_28)] | Cohort | *** | * | ** | 6 |
| Gratzl 1998[[5](#_ENREF_5)] | Cohort | *** | -- | * | 4 |
| Hohfeld 1989[[12](#_ENREF_12)] | Cohort | * | * | * | 3 |
| Hohlfeld 1994[[13](#_ENREF_13)] | Cohort | *** | -- | *** | 6 |
| Hotop 2012[[29](#_ENREF_29)] | Cohort | **** | ** | *** | 9 |
| Kieffer 2008[[30](#_ENREF_30)] | Cohort | *** | -- | *** | 6 |
| Mazzola 2007[[18](#_ENREF_18)] | Cohort | ** | ** | * | 5 |
| Prusa 2015[[1](#_ENREF_1)] | Cohort | **** | ** | ** | 8 |
| Rodrigues 2014[[9](#_ENREF_9)] | Cross-Sectional | **** | -- | ** | 6 |
| Valentini 2009[[31](#_ENREF_31)] | Cohort | **** | * | *** | 8 |
| Valentini 2015[[16](#_ENREF_16)] | Cohort | *** | * | ** | 6 |
| Vergani 1998[[14](#_ENREF_14)] | Cohort | *** | * | *** | 7 |
| Wallon 2010[[32](#_ENREF_32)] | Cohort | *** | -- | *** | 6 |
| Wallon 2013[[33](#_ENREF_33)] | Cohort | *** | * | *** | 7 |

## Assessment of Risk of Bias (methodology)

The Newcastle-Ottawa Scale [[34](#_ENREF_34)] was used to assess the quality of the included observational studies, with the cohort evaluation tool used for cohort and cross-sectional studies. The ratings consisted of evaluation of the representativeness of the exposed cohort; selection of the non-exposed cohort; ascertainment of exposure; demonstration that the outcome of interest was not present at the start of the study; control for gestational age or different trimesters at diagnosis/treatment start; control for additional factors such as treatment duration, treatment delay, or any factors associated with congenital toxoplasmosis; assessment of the outcome; follow-up length; and adequacy of follow-up. The relevant articles were independently rated on each of the items by eight assessors (SY, JH, BB, EA, EM, RM, AC, TS); with each study being evaluated by two assessors. Any disagreements were discussed and resolved by consensus. The rating scale ranges from 0-9, with 9 as the highest quality rating. Results of the quality assessment are presented in **Table S4**.

## Assessment of Risk of Bias (interpretation)

Newcastle-Ottawa scale was used to assess the risk of bias and qualitative heterogeneity across the included studies. Twenty-five studies had a low risk of selection bias [[1-5](#_ENREF_1), [7-9](#_ENREF_7), [13](#_ENREF_13), [14](#_ENREF_14), [16](#_ENREF_16), [17](#_ENREF_17), [19-23](#_ENREF_19), [26-33](#_ENREF_26)]. In contrast, only ten studies had a low risk of comparability bias [[1-3](#_ENREF_1), [17](#_ENREF_17), [18](#_ENREF_18), [24](#_ENREF_24), [26-29](#_ENREF_26)]. Eleven studies had low risk of outcome bias [[2-4](#_ENREF_2), [7](#_ENREF_7), [8](#_ENREF_8), [10](#_ENREF_10), [13](#_ENREF_13), [14](#_ENREF_14), [29-33](#_ENREF_29)]. The country-specific medical practices, such as not treating women who seroconverted later in pregnancy, also led to methodological heterogeneity in the reviewed studies.

1. Summary of meta-analyses results - Mother-to-Child Transmission Rate

| Endpoints (dataset) | Trimester (onset of maternal TP) | Denominator  Rate (95% CI)  p-value | MTCT Rate by treatment group | | | Comments |
| --- | --- | --- | --- | --- | --- | --- |
|  |  |  | Untreated | Treated w/  Spr monotherapy | Treated w/ Spr and/or PSF |  |
| **Results from All 34 studies** | | | | | | |
| **MTCT rate** (All 34) | 1-3 | **#** of **mothers** w/ TP | 356/755 (11 studies) | 470/4312 (12 studies) | 1053/8855 (19 studies) |  |
| Year 1974 - 2016 |  | Rate % (95% CI) | **50.7%** (31.2-70%) | **17.6%** (9.9-26.8%) | **19.5%** (14-25.5%) |  |
|  |  | p-value | -- | <0.001 | <0.001 |  |
| **MTCT rate** (All 34) | 1-3 | **#** of **mothers** w/ TP | 356/755 (11 studies) | 320/3284 (5 studies) | 496/5556 (8 studies) |  |
| Year before 1999 |  | Rate % (95% CI) | **50.7%** (31.2-70%) | **13.6%** (5.7-23.9%) | **12.3%** (8.2-17%) |  |
|  |  | p-value | -- | <0.001 | <0.001 |  |
| **MTCT rate** (All 34) | 1-3 | **#** of **mothers** w/ TP | 356/755 (11 studies) | 19/553 (1 study) | 272/1514 (3 studies) |  |
| Year 1999-2006 |  | Rate % (95% CI) | **50.7%** (31.2-70%) | **3.4%** (2.1-5.1%) | **35.4%** (12.1-63.2%) |  |
|  |  | p-value | -- | <0.001 | <0.001 |  |
| **MTCT rate** (All 34) | 1-3 | **#** of **mothers** w/ TP | 356/755 (11 studies) | 131/475 (6 studies) | 285/1785 (8 studies) |  |
| Year after 2006 |  | Rate % (95% CI) | **50.7%** (31.2-70%) | **24.8%** (6.1-49.9%) | **18.4%** (8.2-31.3%) |  |
|  |  | p-value | -- | <0.001 | <0.001 |  |
| **MTCT rate** (All 34) | 1 | **#** of **mothers** w/ TP | 4/16 (1 study*) | 16/270 (3 studies) | 45/914 (6 studies) | * Desmonts 1979 |
| 1^st^ Trimester |  | Rate % (95% CI) | **25.0%** (6.3-49.5%) | **5.4%** (2.8-8.7%) | **3.2%** (1.9-4.8%) |  |
|  |  | p-value | -- | 0.004 | <0.001 |  |
| **MTCT rate** (All 34)  2^nd^ Trimester | 2 | **#** of **mothers** w/ TP | 7/13 (1 study*) | 33/160 (2 studies) | 123/484 (5 studies) | * Desmonts 1979 |
|  |  | Rate % (95% CI) | **53.8%** (26.1-80.5%) | **20.8%** (12.2-30.9%) | **25.3%** (16.9-34.6%) |  |
|  |  | p-value | -- | 0.006 | 0.021 |  |
| **MTCT rate** (All 34)  3^rd^ Trimester | **3** | **#** of **mothers** w/ TP | 15/23 (1 study*) | 18/39 (2 studies) | 61/110 (4 studies) | * Desmonts 1979 |
|  |  | Rate % (95% CI) | **65.2%** (44.4-83.6%) | **45.6%** (28.1-63.5%) | **54.0%** (35.5-72.1%) |  |
|  |  | p-value | -- | 0.146 | 0.390 |  |
| **MTCT rate** (All 34)  1^st^ & 2^nd^ Trimesters | 1+2 | **#** of **mothers** w/ TP | 11/29 (1 study*) | 49/430 (3 studies) | 168/1398 (6 studies) | * Desmonts 1979 |
|  |  | Rate % (95% CI) | **38.3%** (12.8-67.3%) | **11.5%** (4.4-21.1%) | **12.6%** (6-20.8%) |  |
|  |  | p-value | -- | <0.001 | <0.001 |  |
| **MTCT rate** (33)  excluding Avelino 2014*  Sensitivity analysis | 1-3 | **#** of **mothers** w/ TP | 272/640 (10 studies) | 400/4192 (11 studies) | 983/8735 (18 studies) | * Excluding a South America cohort with exceptionally high MTCT rate (due to highly virulent *T.gondii* strain) |
|  |  | Rate % (95% CI) | **47.7%** (27.4-68.3%) | **14.3%** (8.4-21.4%) | **17.9%** (12.9-23.6%) |  |
|  |  | p-value | -- | <0.001 | <0.001 |  |
| **Results from Key 15 studies** | | | | | | |
| **MTCT rate** (Key 15) | 1-3 | **#** of **mothers** w/ TP | 356/755 (11 studies) | 353/3140 (3 studies) | 671/6645 (8 studies) |  |
|  |  | Rate % (95% CI) | **50.7%** (31.2-70%) | **26.8%** (6.6-54%) | **16.3%** (10.5-23.2%) |  |
|  |  | p-value | -- | <0.001 | <0.001 |  |
| **MTCT rate** (Key 15) | 1 | **#** of **mothers** w/ TP | 4/16 (1 study*) | 14/246 (2 studies) | 39/829 (4 studies) | * Desmonts 1979 |
| 1^st^ Trimester |  | Rate % (95% CI) | **25.0%** (6.3-49.5%) | **5.6%** (2.7-9.4%) | **4.9%** (3-7.2%) |  |
|  |  | p-value | -- | 0.003 | <0.001 |  |
| **MTCT rate** (Key 15) | 2 | **#** of **mothers** w/ TP | 7/13 (1 study*) | 28/144 (1 study) | 80/379 (3 studies) | * Desmonts 1979 |
| 2^nd^ Trimester |  | Rate % (95% CI) | **53.8%** (26.1-80.5%) | **19.4%** (13.4-26.3%) | **21.1%** (17.1-25.4%) |  |
|  |  | p-value | -- | 0.004 | 0.005 |  |
| **MTCT rate** (Key 15) | 3 | **#** of **mothers** w/ TP | 15/23 (1 study*) | 16/36 (1 study) | 43/82 (2 studies) | * Desmonts 1979 |
| 3^rd^ Trimester |  | Rate % (95% CI) | **65.2%** (44.4-83.6%) | **44.4%** (28.5-61%) | **52.1%** (38.3-65.9%) |  |
|  |  | p-value | -- | 0.119 | 0.276 |  |
| **MTCT rate** (Key 15) | 1+2 | **#** of **mothers** w/ TP | 11/29 (1 study*) | 42/390 (2 studies) | 119/1208 (4 studies) | * Desmonts 1979 |
| 1^st^ & 2^nd^ Trimesters |  | Rate % (95% CI) | **38.3%** (12.8-67.3%) | **9.8%** (2.6-20.6%) | **11.2%** (5.3-18.8%) |  |
|  |  | p-value | -- | <0.001 | <0.001 |  |
| **MTCT rate** (14)  Excluding Avelino 2014*  Sensitivity analysis | 1-3 | **#** of **mothers** w/ TP | 272/640 (10 studies) | 283/3020 (2 studies) | 601/6525 (7 studies) | * Excluding a South America cohort with exceptionally high MTCT rate (due to highly virulent *T.gondii* strain) |
|  |  | Rate % (95% CI) | **47.7%** (27.4-68.3%) | **14.2%** (2.7-32.5%) | **12.1%** (8.1-16.8%) |  |
|  |  | p-value | -- | <0.001 | <0.001 |  |

Note: Spr = spiramycin; PSF = pyrimethamine + sulfadiazine or sulfadoxine + folinic acid; MTCT = mother-to-child transmission.

1. Trimester during which the mother acquired acute maternal infection of *T. gondii*.
2. Spr monotherapy = treated with spiramycin until delivery.
3. Spr and/or PSF = (1) treated with spiramycin monotherapy throughout; *or* (2) treated with spiramycin first until diagnosis of fetal infection or until Week 16 to 18 of gestation followed by PSF or PSF alternating with spiramycin.
4. Summary of meta-analysis results – Post-MTCT Outcomes

| Endpoints (dataset) | Trimester (onset of maternal TP) | Denominator  Rate (95% CI)  p-value | Post-MTCT Outcome by treatment group | | |
| --- | --- | --- | --- | --- | --- |
|  |  |  | Untreated | Treated w/  Spr monotherapy | Treated w/ Spr and/or PSF |
| **Mortality** excluding elective terminations *(pre- and postnatal)*  (1974-2006 - Up to 1 year) | 1-3 | **#** of infected **fetuses** | 4/117 (3 studies) | 3/303 (4 studies) | 9/495 (7 studies) |
|  |  | Rate % (95% CI) | **0.1%** (0-3.8%) | **0.3%** (0-5.4%) | **1.3%** (0-4.7%) |
|  |  | p-value | -- | 0.081 | 0.280 |
| **Mortality** excluding elective terminations *(pre- and postnatal)*  (Before 1999 - Up to 1 year) | 1-3 | **#** of infected **fetuses** | 4/117 (3 studies) | 3/295 (3 studies) | 7/421 (5 studies) |
|  |  | Rate % (95% CI) | **0.1%** (0-3.8%) | **0.9%** (0-7.5%) | **1.7%** (0-6.3%) |
|  |  | p-value | -- | 0.099 | 0.259 |
| **Mortality** excluding elective terminations *(pre- and postnatal)*  (After 2006 - Up to 1 year) | 1-3 | **#** of infected **fetuses** | 4/117 (3 studies) | 0/8 (1 studies) | 2/74 (2 studies) |
|  |  | Rate % (95% CI) | **0.1%** (0-3.8%) | **0.0%** (0-20.4%) | **1.1%** (0-6.4%) |
|  |  | p-value | -- | 0.601 | 0.813 |
| **Mortality** excluding elective terminations *(pre- and postnatal)*  (1974-2006 - Beyond 1 year) | 1-3 | **#** of infected **fetuses** | -- | -- | 4/76 (2 studies) |
|  |  | Rate % (95% CI) | -- | -- | **5.0%** (0.8-11.6%) |
|  |  | p-value | -- | -- | -- |
| **Serious/severe sequelae and mortality** including elective terminations *(pre- and postnatal)*  (1974-2006 – Up to 1 year) | 1-3 | **#** of **mothers** w/ TP | 29/266 (4 studies) | 11/487 (3 study) | 28/2239 (6 studies) |
|  |  | Rate % (95% CI) | **21.4%** (2.4-49.7%) | **1.0%** (0-3.5%) | **1.2%** (0-3.7%) |
|  |  | p-value | -- | <0.001 | <0.001 |
|  | 1-3 | **#** of infected **fetuses** | 29/158 (4 studies) | 11/116 (2 study) | 28/308 (5 studies) |
|  |  | Rate % (95% CI) | **36.5%** (5.1-75.2%) | **4.7%** (0-22.9%) | **7.5%** (1.2-17.5%) |
|  |  | p-value | -- | 0.040 | 0.004 |
| **Serious/severe sequelae and mortality** including elective terminations *(pre- and postnatal)*  (Before 1999 – Up to 1 year) | 1-3 | **#** of **mothers** w/ TP | 29/266 (4 studies) | 11/388 (1 studies) | 22/1864 (3 studies) |
|  |  | Rate % (95% CI) | **21.4%** (2.4-49.7%) | **2.8**% (1.4-4.8%) | **1.9**% (0-6.7%) |
|  |  | p-value | -- | <0.001 | <0.001 |
|  | 1-3 | **#** of infected **fetuses** | 29/158 (4 studies) | 11/89 (1 studies) | 22/215 (3 studies) |
|  |  | Rate % (95% CI) | **36.5%** (5.1-75.2%) | **12.4**% (6.2-20.1%) | **10.7**% (0.6-28.8%) |
|  |  | p-value | -- | 0.220 | 0.024 |
| **Serious/severe sequelae and mortality** including elective terminations *(pre- and postnatal)*  (After 2006 – Up to 1 year) | 1-3 | **#** of **mothers** w/ TP | 29/266 (4 studies) | 0/99 (2 study) | 6/375 (3 studies) |
|  |  | % (95% CI) | **21.4%** (2.4-49.7%) | **0.0**% (0-1.9%) | **1.1**% (0.1-2.6%) |
|  |  | p-value | -- | <0.001 | <0.001 |
|  | 1-3 | **#** of infected **fetuses** | 29/158 (4 studies) | 0/27 (1 study) | 6/93 (2 studies) |
|  |  | Rate % (95% CI) | **36.5%** (5.1-75.2%) | **0.0**% (0-6.3%) | **3.6**% (0-16.8%) |
|  |  | p-value | -- | 0.015 | 0.008 |
| **Serious/severe sequelae and mortality** including elective terminations *(pre- and postnatal)*  (1974-2006 - Beyond 1 year) | 1-3 | **#** of **mothers** w/ TP | -- | 3/154 (1 study) | 63/2171 (3 studies) |
|  |  | Rate % (95% CI) | -- | **1.9**% (0.2-4.9%) | **2.8**% (2.2-3.6%) |
|  |  | p-value | -- | -- | -- |
|  | 1-3 | **#** of infected **fetuses** | -- | 3/12 (1 study) | 64/158 (4 studies) |
|  |  | Rate % (95% CI) | -- | **25**% (3.9-53.9%) | **33.2**% (12.5-57.8%) |
|  |  | p-value | -- | -- | -- |
| **Serious/severe sequelae and mortality** including elective terminations *(pre- and postnatal)*  (Before 1999 - Beyond 1 year) | 1-3 | **#** of **mothers** w/ TP | -- | 3/154 (1 study) | 63/2171 (3 studies) |
|  |  | Rate % (95% CI) | -- | **1.9**% (0.2-4.9%) | **2.8**% (2.2-3.6%) |
|  |  | p-value | -- | -- | -- |
|  | 1-3 | **#** of infected **fetuses** | -- | 3/12 (1 study) | 63/140 (3 studies) |
|  |  | Rate % (95% CI) | -- | **25**% (3.9-53.9%) | **44.6**% (25-65.1%) |
|  |  | p-value | -- | -- | -- |
| **Serious/severe sequelae and mortality** including elective terminations *(pre- and postnatal)*  (1999-2006 - Beyond 1 year) | 1-3 | **#** of infected **fetuses** | -- | -- | 1/18 (1 study) |
|  |  | Rate % (95% CI) | -- | -- | **5.6**% (0-22.3%) |
|  |  | p-value | -- | -- | -- |
| **Mild/moderate/severe sequelae and infant mortality** *(postnatal only)*  (1974-2016 – Up to 1 year) | 1-3 | **#** of **children** w/ CT | 102/191 (4 studies) | 70/193 (4 studies) | 90/331 (8 studies) |
|  |  | Rate % (95% CI) | **65.8%** (27.6-95.9%) | **32.6%** (16.4-51.1%) | **21.6%** (10-35.6%) |
|  |  | p-value | -- | <0.001 | <0.001 |
| **Mild/moderate/severe sequelae and infant mortality** *(postnatal only)*  (Before 1999 – Up to 1 year) | 1-3 | **#** of **children** w/ CT | 102/191 (4 studies) | 23/88 (1 study) | 25/97 (2 studies) |
|  |  | Rate % (95% CI) | **65.8%** (27.6-95.9%) | **26.1%** (17.4-35.9%) | **24.8%** (16.2-34.5%) |
|  |  | p-value | -- | <0.001 | <0.001 |
| **Mild/moderate/severe sequelae and infant mortality** *(postnatal only)*  (1999-2006 – Up to 1 year) | 1-3 | **#** of **children** w/ CT | 102/191 (4 studies) | -- | 1/18 (1 study) |
|  |  | Rate % (95% CI) | **65.8%** (27.6-95.9%) | -- | **5.6%** (0-22.3%) |
|  |  | p-value | -- | -- | <0.001 |
| **Mild/moderate/severe sequelae and infant mortality** *(postnatal only)*  (After 2006 – Up to 1 year) | 1-3 | **#** of **children** w/ CT | 102/191 (4 studies) | 47/105 (3 studies) | 64/216 (5 studies) |
|  |  | Rate % (95% CI) | **65.8%** (27.6-95.9%) | **35.3%** (14.1-59.7%) | **23.5%** (5.9-46.6%) |
|  |  | p-value | -- | 0.155 | <0.001 |
| **Mild/moderate/severe sequelae and infant mortality** (postnatal only)  (1974-2016 – Beyond 1 year) | 1-3 | **#** of **children** w/ CT | -- | 1/10 (1 study) | 40/128 (6 studies) |
|  |  | Rate % (95% CI) | -- | **10%** (0-38.1%) | **31.5%** (20.8-43.1%) |
|  |  | p-value | -- | -- | -- |
| **Mild/moderate/severe sequelae and infant mortality** (postnatal only)  (Before 1999 – Beyond 1 year) | 1-3 | **#** of **children** w/ CT | -- | 1/10 (1 study) | 33/110 (4 studies) |
|  |  | Rate % (95% CI) | -- | **10%** (0-38.1%) | **29.9%** (17.3-44%) |
|  |  | p-value | -- | -- | -- |
| **Mild/moderate/severe sequelae and infant mortality** (postnatal only)  (1999-2006 – Beyond 1 year) | 1-3 | **#** of **children** w/ CT | -- | -- | 7/18 (1 study) |
|  |  | Rate % (95% CI) | -- | -- | **38.9%** (17.4-62.7%) |
|  |  | p-value | -- | -- | -- |
| **Mild/moderate/severe sequelae and infant mortality** *(postnatal only)*  (After 2006 – Beyond 1 year) | 1-3 | **#** of **children** w/ CT | -- | -- | 11/107 (1 study) |
|  |  | Rate % (95% CI) | -- | -- | **10.3%** (5.1-16.8%) |
|  |  | p-value | -- | -- | -- |
| **Subclinical disease** *(in live‑born children)*  (1974-2016 – Up to 1 year) | 1-3 | **#** of **children** w/ CT | 89/188 (3 studies) | 116/185 (3 studies) | 172/258 (5 studies) |
|  |  | Rate % (95% CI) | **43.4%** (9.4-81.3%) | **63.4%** (43.3-81.3%) | **67.5%** (52.9-80.6%) |
|  |  | p-value | -- | 0.003 | <0.001 |
| **Subclinical disease** *(in live‑born children)*  (Before 1999 – Up to 1 year) | 1-3 | **#** of **children** w/ CT | 89/188 (3 studies) | 65/88 (1 study) | 121/161 (3 studies) |
|  |  | Rate % (95% CI) | **43.4%** (9.4-81.3%) | **73.9%** (64.1-82.6%) | **75.4%** (66.7-83.1%) |
|  |  | p-value | -- | <0.001 | <0.001 |
| **Subclinical disease** *(in live‑born children)*  (After 2006 – Up to 1 year) | 1-3 | **#** of **children** w/ CT | 89/188 (3 studies) | 51/97 (2 studies) | 51/97 (2 studies) |
|  |  | Rate % (95% CI) | **43.4%** (9.4-81.3%) | **57.0%** (32.9-79.5%) | **57.0%** (32.9-79.5%) |
|  |  | p-value | -- | 0.402 | 0.402 |
| **Subclinical disease** *(in live‑born children)*  (1974-2006 – Beyond 1 year) | 1-3 | **#** of **children** w/ CT | -- | 9/10 (1 study) | 160/221 (6 studies) |
|  |  | Rate % (95% CI) | -- | **90%** (61.9-100%) | **73.6%** (63.9-82.4%) |
|  |  | p-value | -- | -- | -- |
| **Subclinical disease** *(in live‑born children)*  (Before 1999 – Beyond 1 year) | 1-3 | **#** of **children** w/ CT | -- | 9/10 (1 study) | 77/110 (4 studies) |
|  |  | Rate % (95% CI) | -- | 90% (61.9-100%) | 70.1% (56-82.7%) |
|  |  | p-value | -- | -- | -- |
| **Subclinical disease** *(in live‑born children)*  (After 2006 – Beyond 1 year) | 1-3 | **#** of **children** w/ CT | -- | -- | 83/111 (2 studies) |
|  |  | Rate % (95% CI) | -- | -- | 82.8% (57.2-99.1%) |
|  |  | p-value | -- | -- | -- |
| **Chorioretinitis**  (1974-2016 – Up to 1 year) | 1-3 | **#** of **children** w/ CT | 3/19 (1 study) | 1/27 (1 study) | 18/134 (2 studies) |
|  |  | Rate % (95% CI) | **15.8%** (2.3-36.2%) | **3.7%** (0-15.2%) | **10.3%** (1.7-23.9%) |
|  |  | p-value | -- | 0.152 | 0.780 |
| **Chorioretinitis**  (After 2006 – Up to 1 year) | 1-3 | **#** of **children** w/ CT | 3/19 (1 study) | 1/27 (1 study) | 18/134 (2 studies) |
|  |  | Rate % (95% CI) | **15.8%** (2.3-36.2%) | **3.7%** (0-15.2%) | **10.3%** (1.7-23.9%) |
|  |  | p-value | -- | 0.152 | 0.780 |
| **Chorioretinitis**  (1974-2006 – Beyond 1 year) | 1-3 | **#** of **children** w/ CT | 4/25 (1 study) | 11/52 (1 study) | 97/512 (6 studies) |
|  |  | Rate % (95% CI) | **16%** (3.8-33.4%) | **21.2%** (11-33.4%) | **17.7%** (13.5-22.3%) |
|  |  | p-value | -- | 0.593 | 0.713 |
| **Chorioretinitis**  (Before 1999 – Beyond 1 year) | 1-3 | **#** of **children** w/ CT | 4/25 (1 study) | -- | 18/100 (3 studies) |
|  |  | Rate % (95% CI) | **16%** (3.8-33.4%) | **--** | **17.7%** (10.5-26.2%) |
|  |  | p-value | -- | -- | 0.814 |
| **Chorioretinitis**  (After 2006 – Beyond 1 year) | 1-3 | **#** of **children** w/ CT | 4/25 (1 study) | 11/52 (1 study) | 79/412 (3 studies) |
|  |  | Rate % (95% CI) | **16%** (3.8-33.4%) | **21.2%** (11-33.4%) | **17.5%** (11.7-24.2%) |
|  |  | p-value | -- | 0.593 | 0.694 |
| **Mortality** excluding elective terminations *(pre- and postnatal)*  (Up to 1 year) | 1-3 | **#** of infected **fetuses** | 4/117 (3 studies) | 1/283 (2 studies) | 7/475 (5 studies) |
|  |  | Rate % (95% CI) | **0.3**% (0-3.8%) | **0.2%** (0-2.2%) | **1.4%** (0-4.4%) |
|  |  | p-value | -- | 0.014 | 0.163 |
| **Mortality** excluding elective terminations *(pre- and postnatal)*  (Beyond 1 year) | 1-3 | **#** of infected **fetuses** | -- | -- | 4/76 (2 studies) |
|  |  | Rate % (95% CI) | -- | -- | 5% (0.8-11.6%) |
|  |  | p-value | -- | -- | -- |
| **Serious/severe sequelae and mortality** including elective terminations *(pre- and postnatal)*  (Up to 1 year) | 1-3 | **#** of **mothers** w/ TP | 29/266 (4 studies) | 11/388 (1 study) | 28/2140 (4 studies) |
|  |  | Rate % (95% CI) | **21.4%** (2.4-49.7%) | **2.8%** (1.4-4.8%) | **1.9%** (0.1-5.5%) |
|  |  | p-value | -- | <0.001 | <0.001 |
|  | 1-3 | **#** of infected **fetuses** | 29/158 (4 studies) | 11/89 (1 study) | 28/281 (4 studies) |
|  |  | Rate % (95% CI) | **36.5%** (5.1-75.2%) | **12.4%** (6.2-20.1%) | **10.1%** (2.3-22.1%) |
|  |  | p-value | -- | 0.220 | 0.012 |
| **Serious/severe sequelae and mortality** including elective terminations *(pre- and postnatal)*  (Beyond 1 year) | 1-3 | **#** of **mothers** w/ TP | -- | -- | 60/2017 (2 studies) |
|  |  | Rate % (95% CI) | -- | -- | **3%** (2.3-3.8%) |
|  |  | p-value | -- | -- | -- |
|  | 1-3 | **#** of infected **fetuses** | -- | -- | 60/128 (2 studies) |
|  |  | Rate % (95% CI) | -- | -- | **50.9%** (27.3-74.4%) |
|  |  | p-value | -- | -- | -- |
| **Mild/moderate/severe sequelae and infant mortality** *(postnatal only)*  (Up to 1 year) | 1-3 | **#** of **children** w/ CT | 102/191 (4 studies) | 61/158 (2 studies) | 78/269 (4 studies) |
|  |  | % (95% CI) | **65.8%** (27.6-95.9%) | **39.6%** (14.7-67.8%) | **25.3%** (7.8-47.5%) |
|  |  | p-value | -- | 0.006 | <0.001 |
| **Mild/moderate/severe sequelae and infant mortality** *(postnatal only)*  (Beyond 1 year) | 1-3 | **#** of **children** w/ CT | -- | -- | 32/100 (4 studies) |
|  |  | % (95% CI) | -- | -- | **33.7%** (20.7-48%) |
|  |  | p-value | -- | -- | -- |
| **Subclinical Disease** *(in live‑born children)*  (Up to 1 year) | 1-3 | **#** of **children** w/ CT | 89/188 (3 studies) | 97/158 (2 studies) | 153/231 (4 studies) |
|  |  | % (95% CI) | **43.4%** (9.4-81.3%) | **60.4%** (32.2-85.3%) | **66.8%** (49-82.5%) |
|  |  | p-value | -- | 0.009 | <0.001 |
| **Subclinical Disease** (In live-born children)  (Beyond 1 year) | 1-3 | **#** of **children** w/ CT | -- | -- | 151/211 (5 studies) |
|  |  | % (95% CI) | -- | -- | **71.9%** (61.9-81.1%) |
|  |  | p-value | -- | -- | -- |
| **Chorioretinitis**  (Up to 1 year) | 1-3 | **#** of **children** w/ CT | 3/19 (1 study) | -- | 17/107 (1 study) |
|  |  | % (95% CI) | **15.8%** (2.3-36.2%) | -- | **15.9%** (9.5-23.5%) |
|  |  | p-value | -- | -- | 0.991 |
| **Chorioretinitis**  (Beyond 1 year) | 1-3 | **#** of **children** w/ CT | 4/25 (1 study) | 11/52 (1 study) | 97/512 (6 studies) |

Note: Spr = spiramycin; PSF = pyrimethamine + sulfadiazine or sulfadoxine + folinic acid

a Trimester during which the mother acquired acute maternal infection of *T. gondii*.

b Spr monotherapy = treated with spiramycin until delivery.

c Spr and/or PSF = (1) treated with spiramycin monotherapy throughout; *or* (2) treated with spiramycin first until diagnosis of fetal infection or until Week 16 to 18 of gestation followed by PSF or PSF alternating with spiramycin.

# References

[1] Prusa AR, Kasper DC, Pollak A, Gleiss A, Waldhoer T, Hayde M (2015) The Austrian Toxoplasmosis Register, 1992-2008. Clin Infect Dis 60 (2):e4-e10

[2] Foulon W, Villena I, Stray-Pedersen B, Decoster A, Lappalainen M, Pinon JM, Jenum PA, Hedman K, Naessens A (1999) Treatment of toxoplasmosis during pregnancy: a multicenter study of impact on fetal transmission and children's sequelae at age 1 year. Am J Obstet Gynecol 180 (2 Pt 1):410-415

[3] Gilbert R, Gras L (2003) Effect of timing and type of treatment on the risk of mother to child transmission of Toxoplasma gondii. BJOG 110 (2):112-120

[4] Fricker-Hidalgo H, Cimon B, Chemla C, Darde ML, Delhaes L, L'Ollivier C, Godineau N, Houze S, Paris L, Quinio D, Robert-Gangneux F, Villard O, Villena I, Candolfi E, Pelloux H (2013) Toxoplasma seroconversion with negative or transient immunoglobulin M in pregnant women: myth or reality? A French multicenter retrospective study. J Clin Microbiol 51 (7):2103-2111

[5] Gratzl R, Hayde M, Kohlhauser C, Hermon M, Burda G, Strobl W, Pollak A (1998) Follow-up of infants with congenital toxoplasmosis detected by polymerase chain reaction analysis of amniotic fluid. European journal of clinical microbiology & infectious diseases : official publication of the European Society of Clinical Microbiology 17 (12):853-858

[6] Berrebi A, Kobuch WE, Bessieres MH, Bloom MC, Rolland M, Sarramon MF, Roques C, Fournié A (1994) Termination of pregnancy for maternal toxoplasmosis. Lancet (London, England) 344 (8914):36-39

[7] Bessières MH, Berrebi A, Cassaing S, Fillaux J, Cambus JP, Berry A, Assouline C, Ayoubi JM, Magnaval JF (2009) Diagnosis of congenital toxoplasmosis: prenatal and neonatal evaluation of methods used in Toulouse University Hospital and incidence of congenital toxoplasmosis. Memorias do Instituto Oswaldo Cruz 104 (2):389-392

[8] Daffos F, Forestier F, Capella-Pavlovsky M, Thulliez P, Aufrant C, Valenti D, Cox WL (1988) Prenatal management of 746 pregnancies at risk for congenital toxoplasmosis. The New England journal of medicine 318 (5):271-275

[9] Rodrigues IM, Costa TL, Avelar JB, Amaral WN, Castro AM, Avelino MM (2014) Assessment of laboratory methods used in the diagnosis of congenital toxoplasmosis after maternal treatment with spiramycin in pregnancy. BMC infectious diseases 14:349

[10] Bessières MH, Berrebi A, Rolland M, Bloom MC, Roques C, Cassaing S, Courjault C, Séguéla JP (2001) Neonatal screening for congenital toxoplasmosis in a cohort of 165 women infected during pregnancy and influence of in utero treatment on the results of neonatal tests. European journal of obstetrics, gynecology, and reproductive biology 94 (1):37-45

[11] Desmonts G, Couvreur J (1974) Congenital toxoplasmosis. A prospective study of 378 pregnancies. The New England journal of medicine 290 (20):1110-1116

[12] Hohlfeld P, Daffos F, Thulliez P, Aufrant C, Couvreur J, MacAleese J, Descombey D, Forestier F (1989) Fetal toxoplasmosis: outcome of pregnancy and infant follow-up after in utero treatment. The Journal of pediatrics 115 (5 Pt 1):765-769

[13] Hohlfeld P, Daffos F, Costa JM, Thulliez P, Forestier F, Vidaud M (1994) Prenatal diagnosis of congenital toxoplasmosis with a polymerase-chain-reaction test on amniotic fluid. The New England journal of medicine 331 (11):695-699

[14] Vergani P, Ghidini A, Ceruti P, Strobelt N, Spelta A, Zapparoli B, Rescaldani R (1998) Congenital toxoplasmosis: efficacy of maternal treatment with spiramycin alone. American journal of reproductive immunology (New York, NY : 1989) 39 (5):335-340

[15] Desmonts G CJ (1979) Congenital toxoplasmosis: A prospective study of the offspring of 542 women who acquired toxoplasmosis during pregnancy. Pathophysiology of congenital disease. Thalhammer O, Baumgarten K, Pollack A (eds): Perinatal Medicine, Sixth European Congress

[16] Valentini P, Buonsenso D, Barone G, Serranti D, Calzedda R, Ceccarelli M, Speziale D, Ricci R, Masini L (2015) Spiramycin/cotrimoxazole versus pyrimethamine/sulfonamide and spiramycin alone for the treatment of toxoplasmosis in pregnancy. Journal of perinatology : official journal of the California Perinatal Association 35 (2):90-94

[17] Campello Porto L, Duarte EC (2012) Association between the risk of congenital toxoplasmosis and the classification of toxoplasmosis in pregnant women and prenatal treatment in Brazil, 1994-2009. International journal of infectious diseases : IJID : official publication of the International Society for Infectious Diseases 16 (7):e480-e486

[18] Mazzola A, Casuccio A, Romano A, Schimmenti MG, Titone L, Di Carlo P (2007) Diagnostic problems and postnatal follow-up in congenital toxoplasmosis. Minerva pediatrica 59 (3):207-213

[19] Avci ME, Arslan F, Çiftçi S, Ekiz A, Tüten A, Yildirim G, Madazli R (2016) Role of spiramycin in prevention of fetal toxoplasmosis. The journal of maternal-fetal & neonatal medicine : the official journal of the European Association of Perinatal Medicine, the Federation of Asia and Oceania Perinatal Societies, the International Society of Perinatal Obstetricians 29 (13):2073-2076

[20] Avelino MM, Amaral WN, Rodrigues IM, Rassi AR, Gomes MB, Costa TL, Castro AM (2014) Congenital toxoplasmosis and prenatal care state programs. BMC Infect Dis 14:33

[21] Berrébi A, Assouline C, Bessières MH, Lathière M, Cassaing S, Minville V, Ayoubi JM (2010) Long-term outcome of children with congenital toxoplasmosis. American journal of obstetrics and gynecology 203 (6):552.e551-552.e556

[22] Brézin AP, Thulliez P, Couvreur J, Nobré R, McLeod R, Mets MB (2003) Ophthalmic outcomes after prenatal and postnatal treatment of congenital toxoplasmosis. Am J Ophthalmol 135 (6):779-784

[23] Cortina-Borja M, Tan HK, Wallon M, Paul M, Prusa A, Buffolano W, Malm G, Salt A, Freeman K, Petersen E, Gilbert RE (2010) Prenatal treatment for serious neurological sequelae of congenital toxoplasmosis: an observational prospective cohort study. PLoS Med 7 (10):e1000351

[24] Faucher B, Garcia-Meric P, Franck J, Minodier P, Francois P, Gonnet S, L'Ollivier C, Piarroux R (2012) Long-term ocular outcome in congenital toxoplasmosis: a prospective cohort of treated children. The Journal of infection 64 (1):104-109

[25] Galanakis E, Manoura A, Antoniou M, Sifakis S, Korakaki E, Hatzidaki E, Lambraki D, Tselentis Y, Giannakopoulou C (2007) Outcome of toxoplasmosis acquired during pregnancy following treatment in both pregnancy and early infancy. Fetal diagnosis and therapy 22 (6):444-448

[26] Gilbert RE, Gras L, Wallon M, Peyron F, Ades AE, Dunn DT (2001) Effect of prenatal treatment on mother to child transmission of Toxoplasma gondii: retrospective cohort study of 554 mother-child pairs in Lyon, France. Int J Epidemiol 30 (6):1303-1308

[27] Gras L, Gilbert RE, Ades AE, Dunn DT (2001) Effect of prenatal treatment on the risk of intracranial and ocular lesions in children with congenital toxoplasmosis. Int J Epidemiol 30 (6):1309-1313

[28] Gras L, Wallon M, Pollak A, Cortina-Borja M, Evengard B, Hayde M, Petersen E, Gilbert R (2005) Association between prenatal treatment and clinical manifestations of congenital toxoplasmosis in infancy: a cohort study in 13 European centres. Acta Paediatr 94 (12):1721-1731

[29] Hotop A, Hlobil H, Gross U (2012) Efficacy of rapid treatment initiation following primary Toxoplasma gondii infection during pregnancy. Clin Infect Dis 54 (11):1545-1552

[30] Kieffer F, Wallon M, Garcia P, Thulliez P, Peyron F, Franck J (2008) Risk factors for retinochoroiditis during the first 2 years of life in infants with treated congenital toxoplasmosis. The Pediatric infectious disease journal 27 (1):27-32

[31] Valentini P, Annunziata ML, Angelone DF, Masini L, De Santis M, Testa A, Grillo RL, Speziale D, Ranno O (2009) Role of spiramycin/cotrimoxazole association in the mother-to-child transmission of toxoplasmosis infection in pregnancy. Eur J Clin Microbiol Infect Dis 28 (3):297-300

[32] Wallon M, Franck J, Thulliez P, Huissoud C, Peyron F, Garcia-Meric P, Kieffer F (2010) Accuracy of real-time polymerase chain reaction for Toxoplasma gondii in amniotic fluid. Obstet Gynecol 115 (4):727-733

[33] Wallon M, Peyron F, Cornu C, Vinault S, Abrahamowicz M, Kopp CB, Binquet C (2013) Congenital toxoplasma infection: monthly prenatal screening decreases transmission rate and improves clinical outcome at age 3 years. Clinical infectious diseases : an official publication of the Infectious Diseases Society of America 56 (9):1223-1231

[34] Wells GA, Shea B, O'Connell D, Peterson J, Welch V, Losos M, Tugwell R (2019) The Newcastle-Ottawa Scale (NOS) for assessing the quality of nonrandomised studies in meta-analyses, <http://www.ohri.ca/programs/clinical_epidemiology/oxford.asp>
